# Enantioselective total synthesis of the unnatural enantiomer of quinine†
†Electronic supplementary information (ESI) available. See DOI: 10.1039/c9sc03879e


**DOI:** 10.1039/c9sc03879e

**Published:** 2019-09-27

**Authors:** Shinya Shiomi, Remi Misaka, Mayu Kaneko, Hayato Ishikawa

**Affiliations:** a Department of Chemistry , Graduate School of Science and Technology , Kumamoto University , 2-39-1, Kurokami, Chuo-ku , Kumamoto 860-8555 , Japan . Email: h_ishikawa@kumamoto-u.ac.jp; b Faculty of Advanced Science and Technology , Kumamoto University , 2-39-1, Kurokami, Chuo-ku , Kumamoto 860-8555 , Japan

## Abstract

A practical enantioselective total synthesis of the unnatural (+)-quinine and (–)-9-*epi*-quinine enantiomers, which are important organocatalysts, is reported. The key transformation is a successive organocatalytic formal aza [3 + 3] cycloaddition/Strecker-type cyanation reaction to form an optically active tetrasubstituted piperidine derivative. This organocatalytic reaction proceeded in high yield and gave excellent enantiomeric excess with only 0.5 mol% catalyst loading. In addition, an imidate group, derived from a cyano group, was incorporated in the strategy for site-selective modification of the C4-alkyl chiral piperidine ring of quinine. Furthermore, an efficient coupling between the quinuclidine precursor and dihydroquinoline unit was achieved on a gram scale. The 15-step (LLS) synthetic protocol provided both (+)-quinine and (–)-9-*epi*-quinine, each with 16% overall yield.

## Abstract 2

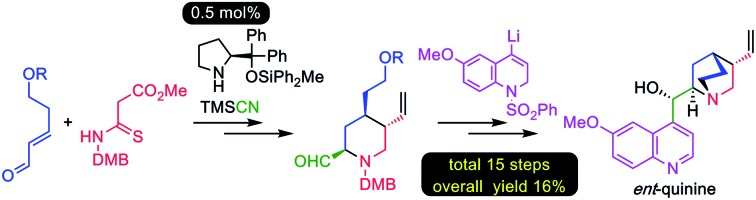
A practical enantioselective total synthesis of the unnatural (+)-quinine and (–)-9-*epi*-quinine enantiomers, which are important organocatalysts, is reported.

## Introduction

**Fig. 1:**
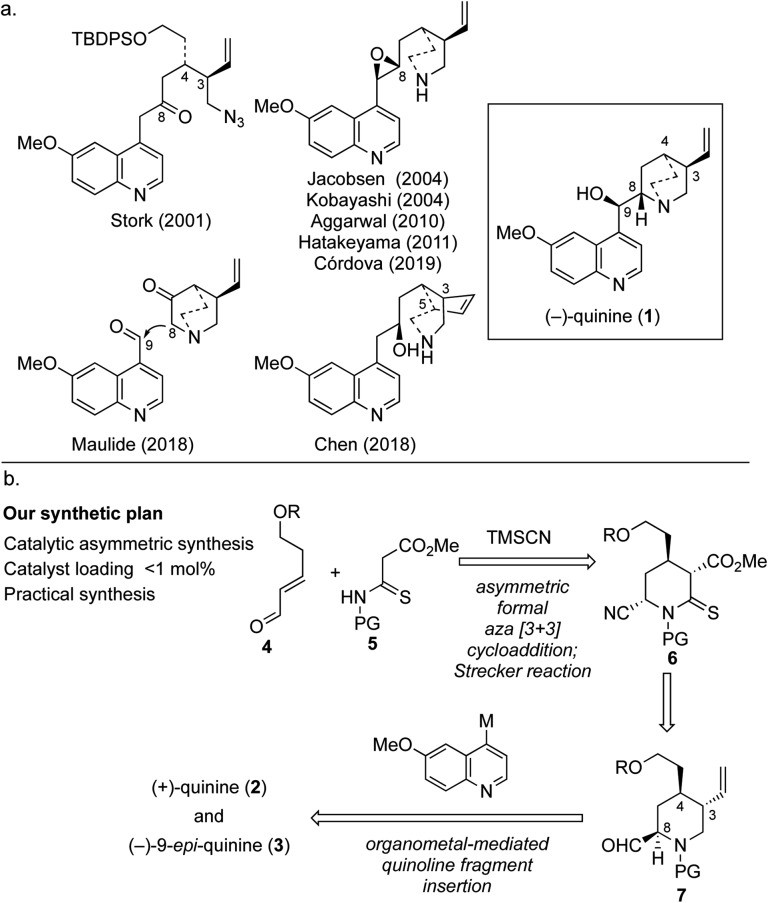
(a) An overview of a previous synthetic intermediate of quinine. (b) Our synthetic plan.

For more than a century, (–)-quinine (**1**), which is widely known as an antimalarial agent,^1^ has attracted intense interest from both academia and industry. By the end of the 20th century, cinchona alkaloids, which include **1**, became game-changing materials in the field of catalytic enantioselective synthesis because these alkaloids and their derivatives work as irreplaceable chiral ligands and organocatalysts.^2^ There is no doubt that the usefulness of these compounds has been boosted by the ongoing efforts of synthetic chemists. However, the chiral source material is derived from cinchona alkaloids, and the latter are only available in economically viable quantities from natural sources. Although quinine and quinidine are diastereomers with the same stereochemical configuration at C3 and C4, fortunately, they have been found to be pseudoenantiomers with respect to chiral ligands and catalysts.^3^ However, given that neither the reactivity nor enantioselectivity of the compounds is identical, with both depending on the reaction conditions, an efficient and scalable synthesis of the unnatural enantiomer (+)-quinine (**2**) has been in high demand. The synthesis of cinchona alkaloids has advanced since the first (formal) total synthesis of quinine (**1**) was achieved by Woodward and Doering.^4^ The first asymmetric total synthesis of quinine was reported by Stork in 2001; this approach featured construction of C3 and C4 contiguous chiral centers and quinuclidine synthesis using reductive amination followed by S_N_2 cyclization (Fig. 1a).^5^ Subsequently, the late-stage construction of the quinuclidine scaffold through N1–C8 bond formation was developed by Jacobsen, and this represents one of the most powerful synthetic strategies for the synthesis of cinchona alkaloids.^6^ This strategy has the clear advantage that it can be used to obtain either quinine or quinidine from the same intermediate. Furthermore, two elegant syntheses were recently reported in 2018.^7^,^8^ Maulide's synthesis features incorporation of the vinyl group through C–H activation and an innovative C8–C9 bond disconnection.^7^ In the same year, a stereoselective divergent synthesis of quinine and quinidine based on local desymmetrization at the C3 and C5 positions was reported by Chen.^8^ Although several known synthetic pathways can be used for the preparation of (+)-quinine (**2**) on a reasonable scale, we envisioned a synthetic design that could allow a practical synthesis of **2** through direct coupling between a quinoline unit and quinuclidine precursor that would expand the diversity of the aromatic portion and facilitate the development of novel organocatalysts (Fig. 1b). In addition, this strategic bond disconnection also allows the generation of (–)-9-*epi*-quinine (**3**), which is a known precursor of urea, thiourea, and primary amine organocatalysts.^9^

The synthetic plan for our approach is shown in Fig. 1b. We envisioned that three chiral centers would be constructed by using our previously developed multisubstituted chiral piperidine synthesis based on a secondary amine organocatalyzed formal aza [3 + 3] cycloaddition reaction^10^ followed by Strecker cyanation. This key sequence allows highly functionalized C-4 alkyl piperidine derivative **6** to be obtained, which can be transformed into quinuclidine precursor **7**, a key unit of both **2** and **3**. Initially, we considered coupling the quinoline fragment with quinuclidine 2-carbaldehyde; however, difficulties associated with controlling the stereochemistry of the aldehyde moiety on the quinuclidine ring were anticipated. Thus, we expected to introduce the quinoline fragment by using the thermodynamically controlled aldehyde attached on the chiral piperidine. To complete the total synthesis, direct coupling of the quinoline unit through nucleophilic addition of the metal complex of quinoline derivative to the C9 position of **7** followed by quinuclidine formation was designed.

Herein we report a practical and enantioselective total synthesis of (+)-**2** and (–)-**3** using only 0.5 mol% chiral source over 15 steps in 16% overall yield.

## Results and discussion

**Scheme 1:**
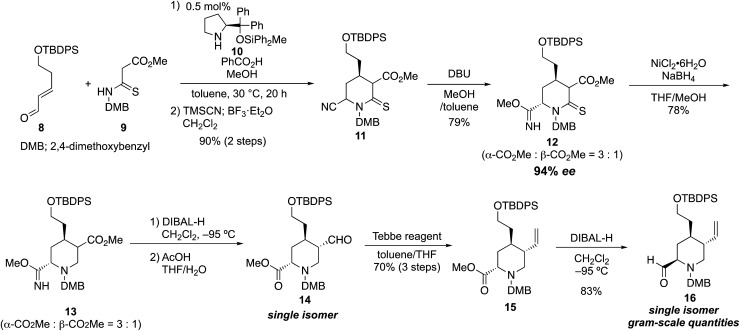
Preparation of the key piperidine compound **16**.

Our synthesis commenced with the enantioselective construction of the fully substituted piperidine **11** by using our reported asymmetric formal aza [3 + 3] cycloaddition reaction employing diphenylprolinol diphenylmethylsilyl ether catalyst **10** (ref. 11) (Scheme 1). The methodology required less than 1 mol% catalyst, and chiral 4-alkyl piperidine-2-ol derivatives were obtained in excellent yield and enantiomeric excess (up to 97% ee) within acceptable reaction time (<50 h).^10^ The reactivity of the nucleophile was increased by the ease of enolization of the thiocarbonyl group and by the addition of suitable additives (1.0 equiv. of benzoic acid and 3.0 equiv. of MeOH). Thus, treatment of known 5-hydroxypentenal derivative **8** (1.1 equiv.), which was prepared in two steps,^12^ and thiomalonamate **9** (1.0 equiv.), which was prepared in a one-pot operation from commercially available 1,3-dimethoxybenzylamine (see details in the ESI†) in the presence of 0.5 mol% catalyst **10**, benzoic acid (1.0 equiv.), and MeOH (3.0 equiv., toluene, 30 °C, 20 h), provided the corresponding chiral 4-alkyl piperidine-2-ol. Cyanation of the hemiaminal moiety of the crude diastereomeric product mixture formed from the formal aza [3 + 3] cycloaddition reaction was carried out directly in a Strecker-type cyanation reaction. Thus, 6.0 equiv. of TMSCN in the presence of 1.2 equiv. of BF_3_·Et_2_O was added to the reaction mixture, and the desired cyano δ-thiolactam **11** was obtained in 90% yield over two steps as a mixture of three diastereomers. Elaboration of **11** toward the tetrasubstituted piperidine intermediate **16** required site-selective reduction among the ester, nitrile, and thiolactam, followed by one-carbon elongation to form the terminal olefin. We initially tried several derivatizations with the nitrile group in place. However, most of the reaction conditions ultimately led to difficulties resulting from removal or reduction of the cyano group; therefore, we explored the derivatization of the cyano group first. After several experiments, we discovered the electron-deficient cyano group near the thiolactam was easily transformed into methyl imidate, which resists reduction conditions (see below). Thus, the diastereomer mixture of **11** was treated with DBU in the presence of MeOH to provide methyl imidate **12**. At this stage, the two major diastereomers were isolated (79%, dr = α-CO_2_Me/β-CO_2_Me = 3 : 1). The enantiomeric excess of each diastereomer was 94% ee. The stereochemistry of both diastereomers was determined by analysis of the coupling constants in ^1^H NMR spectra (see details in the ESI†). We then confirmed that the cyano anion was predominantly introduced from the axial site in the Strecker reaction. With imidate **12** as a mixture of two diastereomers, the thiolactam-selective reduction was achieved by treatment with nickel boride, generated *in situ* from NiCl_2_·6H_2_O (3.0 equiv.) and NaBH_4_ (12.0 equiv., THF, MeOH, –20 °C, 5 min, 78%)^13^ to provide piperidine derivative **13** as a mixture of two diastereomers. The subsequent ester-selective reduction with diisobutylaluminum hydride (DIBAL-H, 5.0 equiv., CH_2_Cl_2_, –95 °C, 2 h) succeeded and the desired aldehyde was obtained without reduction of the imidate moiety. Subsequently, the methyl imidate moiety was hydrolyzed under mild acidic conditions (AcOH/THF/H_2_O = 1 : 30 : 6, rt). At the same time, the aldehyde was also isomerized to the thermodynamically more stable *trans* form, and the desired methyl ester **14** was obtained as a single diastereomer. As a result, by using the protocol reported herein, the cyano group was easily transformed into the ester *via* the imidate, while not having effected by the reduction.

The introduction of the vinyl group by using a Wittig reagent with strong basicity was not suitable for **14**; the reaction afforded low yield (<30%) because of enol formation and an unexpected degradation *via* retro-Mannich reaction. On the other hand, the one-carbon elongation of **14** was achieved upon treatment with Lewis acidic Tebbe reagent (1.1 equiv., toluene/THF, 0 to 23 °C, 2 h) to provide **15** in high yield (70%, over three steps). The C6 ester was then reduced with DIBAL-H (3.0 equiv., CH_2_Cl_2_, –95 °C, 2.5 h) to provide tetrasubstituted piperidine derivative **16** in excellent yield (83%). As expected, after purification by silica gel column chromatography, the aldehyde in **16** spontaneously adopted the equatorial position in the six-membered ring system, which possesses the desired configuration for **2** and **3**. Chiral piperidine **16** constitutes a potential key intermediate to prepare novel organocatalysts, and it was obtained in gram-scale quantities in eight steps in 32% overall yield from thiomalonamate **9** using only 0.5 mol% chiral source. In our established protocol, the C4 chiral center that was constructed by organocatalytic asymmetric reaction was used to control the configuration at the other two stereocenters (C3 and C6) through thermodynamic isomerization reactions. As a result, we could employ any of the diastereomers to prepare **16**.

**Scheme 2:**
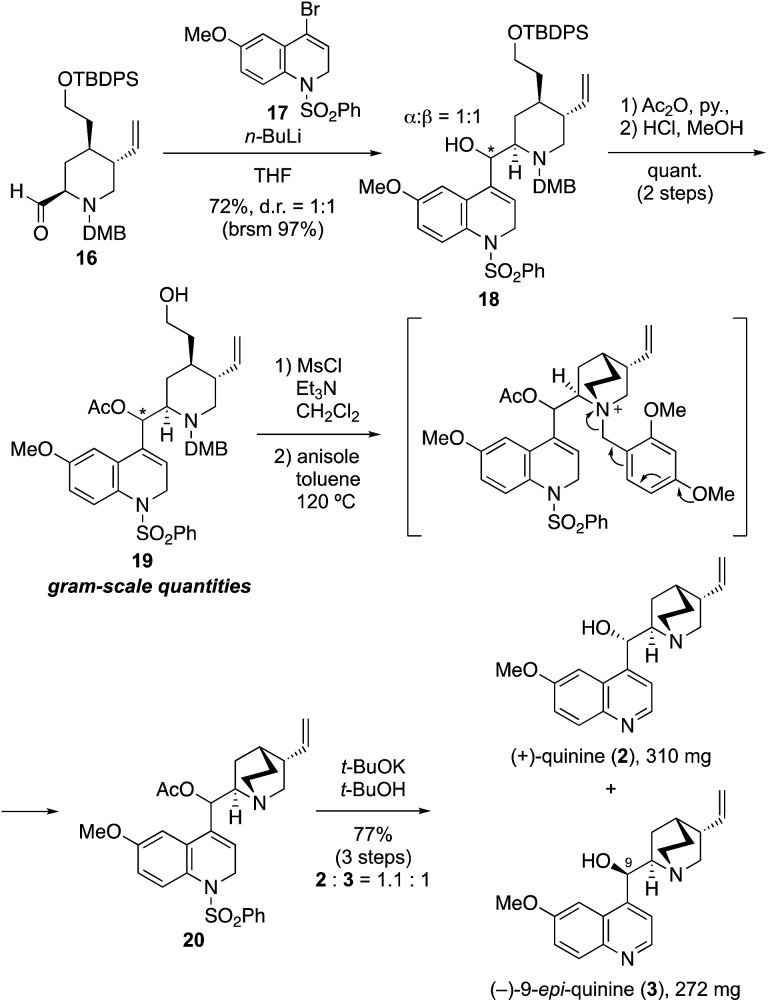
Total synthesis of (+)-quinine and (–)-9-*epi*-quinine.

Although we initially planned to introduce the quinoline derivative directly to **16** by using a quinoline metal complex (M = Li, MgBr, MgCl·LiCl, Me_2_Zn, Me_3_Al, LaCl_3_), all attempts failed to provide the coupling product with acceptable yield because of the unavoidable generation of side-products from the homo-coupling of quinoline.^14^ These results indicated that the electrophilicity of 4-bromoquinoline was much higher than that of piperidine-2-carbaldehyde. In addition, the C4 anion generated on the quinoline ring was stabilized by the π-orbitals of the incorporated nitrogen; thus, the nucleophilicity of the quinoline metal complex was not enough to facilitate reaction with aldehyde **16**. We then considered the use of **17** as an alternative nucleophile, given that deconjugation at the 1- and 2-positions on the quinoline ring of this compound was expected to both prevent the side reaction and increase the reactivity (Scheme 2). By using this approach, the desired coupling reaction between the quinuclidine fragment and dihydroquinoline derivative **17** as the deconjugated equivalent of quinoline was achieved (Scheme 2). Thus, the reaction of **17**, which was prepared in four steps from *p*-methoxyaniline,^15^ with *n*-BuLi (1.2 equiv., THF, –90 °C, 30 min) provided the corresponding 4-lithiated dihydroquinoline. The addition of the latter to **16** (THF, –80 °C, 22 h) gave the desired coupling product **18** in good yield (72%, brsm 97%) as a mixture of two diastereomers at the C-9 position (α/β = 1 : 1).

At this stage, with over 2 g of **18** in hand, the stereoisomers could be separated by silica gel column chromatography, and a full optimization of the synthesis of (+)-quinine using the C-9 α-hydroxy isomer was accomplished (see details in ESI†). Thereafter, the simultaneous synthesis of **2** and **3** using the mixture of C-9 stereoisomers was carried out, as shown in Scheme 2. Thus, acetylation of secondary alcohol **18** was accomplished by treatment with acetic anhydride in the presence of DMAP. Quantitative removal of the silyl protecting group with methanolic hydrochloric acid provided **19** in quantitative yield in gram-scale quantities, and subsequent mesylation of **19** with methanesulfonyl chloride (1.5 equiv.) in the presence of Et_3_N (5.0 equiv.) afforded the precursor of the quinuclidine ring formation reaction. The tertiary amine was then rapidly obtained by quinuclidine formation in a thermal and neutral intramolecular S_N_2 reaction in toluene at 120 °C by spontaneous removal of the DMB group of the resulting ammonium salt in the presence of anisole. In this spontaneous removal of the benzyl moiety, the dimethoxy group on the benzene ring was essential; monomethoxy derivatives such as the PMB group did not have sufficient electron density to enable spontaneous removal. In addition, the avoidance of redox processes such as CAN oxidation or hydrogenation to remove the benzyl moiety was crucial to complete the total synthesis. Finally, removal of the acetyl and phenyl sulfone groups of **20** using potassium *t*-butoxide (3.0 equiv., *t*-BuOH, 60 °C, 1.5 h), provided **2** and **3**, respectively, in excellent yield (77% over three steps, **2**/**3** = 1.1 : 1; 310 mg and 272 mg of **2** and **3** were obtained, respectively). The dihydroquinolines were aromatized *via* spontaneous aerobic oxidation or elimination of sulfinic acid under the reaction condition.

**Scheme 3:**
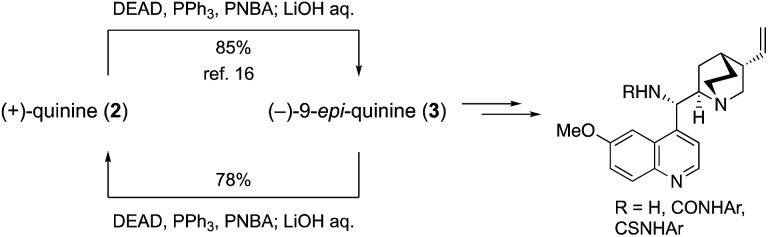
Mutual conversion of (+)-quinine and (–)-9-*epi*-quinine, and application for the synthesis of cinchona catalyst.

The efficient conversion of (+)-quinine (**2**) into (–)-9-*epi*-quinine (**3**) under Mitsunobu conditions was already established,^16^ because **3** was a key intermediate in the recent development of a wide variety of organocatalysts such as primary amine, amide, urea, and thiourea catalysts (Scheme 3).^9^ To establish a mutual conversion, (–)-9-*epi*-quinine (**3**) was then converted into (+)-quinine (**2**). Thus, **3** was treated with *p*-nitrobenzoic acid (PNBA, 1.1 equiv.), DEAD (1.1 equiv.), and PPh_3_ (1.3 equiv., THF, rt, 7 h) to provide the corresponding ester, which was then hydrolyzed with LiOH in one pot to provide **2** in 78% yield. Synthesized (+)-**2** was then recrystallized after treatment with aqueous H_2_SO_4_ solution to provide enantiopure unnatural quinine sulfate hydrate (see details in the ESI†).

## Conclusions

In conclusion, the synthesis of the title compound was achieved by using an enantioselective formal aza [3 + 3] cycloaddition/Strecker reaction sequence, followed by sequential chemoselective reduction/transformation of ester, thiolactam, and cyano groups *via* the transformation of cyano into imidate moieties. In addition, an organolithium-mediated coupling reaction between the dihydroquinoline derivative and piperidine-2-carbaldehyde, followed by construction of the quinuclidine ring with spontaneous removal of the DMB group under neutral conditions were established. The key enantioselective organocatalysis step provides multiply substituted thiolactam as an equivalent of a highly functionalized piperidine derivative and this allowed the straightforward synthesis without any oxidation except for the last step (autooxidation from dihydroquinoline to quinoline). The practical enantioselective total synthesis of (+)-**2** and (–)-**3** required only 0.5 mol% of chiral source (diphenylprolinol silyl ether catalyst **10**) and was achieved in 15 steps, including Mitsunobu conversion, both in 16% overall yield from thiomalonamate **9**. Our synthesis not only provides the unnatural enantiomer of quinine, which is required in many areas of current chemical endeavour, but also enables several aromatic groups to be introduced to key intermediate **16**. Thus, a wide range of new catalysts that were previously difficult to derive from naturally occurring cinchona alkaloids are now available by using our method. Indeed, further development of new classes of cinchona alkaloid-mimic catalysis is under way.

## Conflicts of interest

There are no conflicts to declare.

## Supplementary Material

Supplementary information
