# Correction: Enantioselective total synthesis of the unnatural enantiomer of quinine

**DOI:** 10.1039/c9sc90226k

**Published:** 2019-10-31

**Authors:** Shinya Shiomi, Remi Misaka, Mayu Kaneko, Hayato Ishikawa

**Affiliations:** a Department of Chemistry , Graduate School of Science and Technology , Kumamoto University , 2-39-1, Kurokami, Chuo-ku , Kumamoto 860-8555 , Japan . Email: h_ishikawa@kumamoto-u.ac.jp; b Faculty of Advanced Science and Technology , Kumamoto University , 2-39-1, Kurokami, Chuo-ku , Kumamoto 860-8555 , Japan

## Abstract

Correction for ‘Enantioselective total synthesis of the unnatural enantiomer of quinine’ by Shinya Shiomi *et al.*, *Chem. Sci.*, 2019, DOI: 10.1039/c9sc03879e.



## 

We apologise for an error in ref. 6*e*, which was given as “Y. Jiang, L. Diana, K. Zhang, S. Lin and A. Córdova, *Eur. J. Inorg. Chem.*, 2019, **2019**, 6016–6023”. The correct citation is “Y. Jiang, L. Deiana, K. Zhang, S. Lin and A. Córdova, *Eur. J. Org. Chem.*, 2019, 6016–6023”.

The Royal Society of Chemistry apologises for these errors and any consequent inconvenience to authors and readers.

