# Supplementary material for: Enantioselective total synthesis of the unnatural enantiomer of quinine
Source: Chem Sci. 2019 Sep 27;10(41):9433–7. doi: 10.1039/c9sc03879e (PMC7020653; doi:10.1039/c9sc03879e)
Supplement: Supplementary file 1 [file SC-010-C9SC03879E-s001.pdf]

Electronic Supplementary Material (ESI) for Chemical Science.
This journal is © The Royal Society of Chemistry 2019

Supporting Information

Enantioselective Total Synthesis of the Unnatural Enantiomer of Quinine

Shinya Shiomi,[a] Remi Misaka,[a] Mayu Kaneko,[a] Hayato Ishikawa*[a, b]

[a]Department of Chemistry, Graduate School of Science and Technology, Kumamoto University, 2-39-1, Kurokami, Chuo-ku, Kumamoto 860-8555, Japan

[b]Faculty of Advanced Science and Technology, Kumamoto University 2-39-1, Kurokami, Chuo-ku, Kumamoto 860-8555, Japan

E-mail: h_ishikawa@kumamoto-u.ac.jp

General Remarks: All reactions were monitored by thin-layer chromatography using Merck 60 F254 precoated silica gel plates (0.25 mm thickness). Specific optical rotations were measured using a JASCO P-1020 polarimeter. FT-IR spectra were recorded on a SHIMADZU IR Affinity-IS. ^{1}H and ^{13}C NMR spectra were recorded on a JEOL ECX 500 FT-NMR spectrometer (500 MHz for ^{1}H NMR, 125 MHz for ^{13}C NMR) instrument. Data for ^{1}H NMR are reported as chemical shift (δ ppm), multiplicity (s = singlet, d = doublet, t = triplet, dd = doubledoublet, ddd = doubledoubledoublet, dt = doubletriplet, q = quartet, quint. = quintet, m = multiplet, br = broad), coupling constant (Hz), integration, and assignment. Data for ^{13}C NMR are reported as chemical shift. The high-resolution mass spectra were recorded on a BRUKER impact II. Preparative thin layer chromatography was performed using Merck 60 F254 precoated silica gel plates (0.25 mm thickness). Flash chromatography was performed using silica gel 60N of Kanto Chemical Co. Int., Tokyo, Japan and amino silica gel (SiO_2–NH) of Fuji Silysia Co. Int., Japan. HPLC analysis was performed on a SHIMAZU Prominence series, UV detection monitored at appropriate wavelength respectively, using DAICEL Chiralpak IC (0.46 cm × 25 cm) and DAICEL Chiralpak AS-H (0.46 cm × 25 cm).

Preparation of thiomalonamate **9**

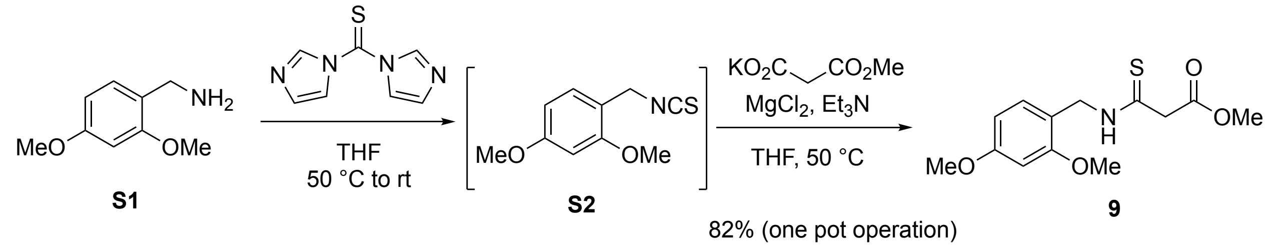

To a solution of 1,1-thiocarbonyldiimidazole (9.09 g, 0.051 mol) in dry THF (170 mL), 2,4-dimethoxybenzylamine (**S1**, 5.65 g, 0.034 mol) was added at 50 °C under Ar atmosphere. The reaction mixture was stirred for 2 h at room temperature to provide 1-(isothiocyanatomethyl)-2,4-dimethoxybenzene (**S2**) solution.

In another flask, to a solution of monomethyl potassium malonate (15.9 g, 0.102 mol) in dry THF (113 mL), $MgCl_2$ (12.3 g, 0.129 mol) was added at 0 °C under Ar atmosphere. Et_3N (22.7 mL, 0.163 mol) was slowly added to the resulting mixture at 0 °C. The resulting suspension mixture was stirred for 1 h at room temperature. The resulting solution of **S2** was added to the resulting monomethyl potassium malonate suspension. The reaction mixture was stirred for 120 h at 50 °C under Ar atmosphere. The resulting mixture was slowly quenched with excess amount of water at 0 °C. The yielded suspension was filtrated with Celite pad and the aqueous layer was extracted three times with EtOAc. The combined organic layer was washed with saturated aqueous NaCl solution, dried over $MgSO_4$, and concentrated under reduced pressure. Flash chromatography (SiO_2, 20% EtOAc / *n*-hexane) provided thiomalonamate **9** (7.87 g, 82%) as a pale yellow oil.

Thiomalonamate **9**

^{1}H NMR (500MHz, $CDCl_3$) δ 9.54 (br s, 1H), 7.25 (m, 1H, overlapped to $CDCl_3$), 6.48 (s, 1H), 4.45 (d, J = 8.0 Hz, 1H), 4.81 (d, J = 5.5 Hz, 2H), 3.86 (s, 3H), 3.86 (s, 2H), 3.80 (s, 3H), 3.71 (s, 3H); ^{13}C NMR (125MHz, $CDCl_3$) δ 192.3, 170.2, 161.1, 158.9, 131.3, 116.5, 104.1, 98.8, 55.5 (2C), 52.5, 48.4, 46.1; IR (neat) ν_{max} 3194, 2964, 1734, 1608, 1558, 1506, 1456, 1423, 1346, 1265, 1213, 1155, 1116, 1031, 1008, 842, 812, 786, 696 cm^{-1}; HRMS (ESI) $[M+H]^+$ calculated for $[C_{13}H_{18}NO_4S]^+$: 284.0951 found : 284.0950.

Organocatalytic formal aza [3+3] cycloaddition reaction and Strecker reaction

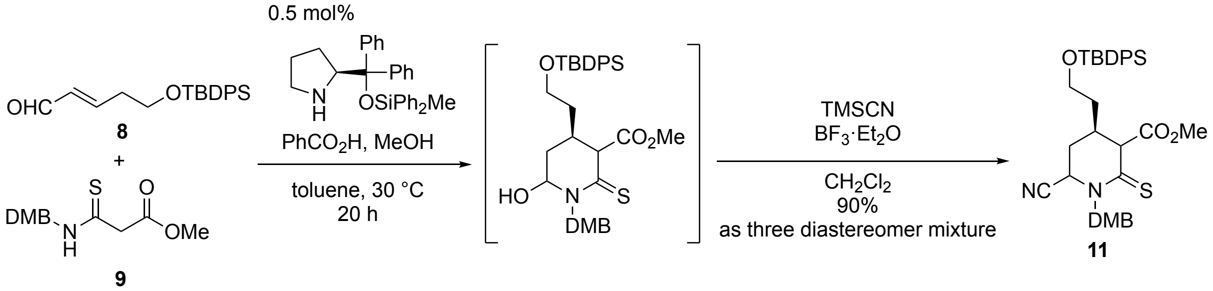

Diphenylprolinol diphenylmethyl silyl ether (**10**, 2.0 mg, 0.0044 mmol) was added to the solution of α, β-

unsaturated aldehyde **8**[s1)] (331 mg, 0.98 mmol), thiomalonamate **9** (252 mg, 0.89 mmol) and benzoic acid (108.6 mg, 0.89 mmol) in toluene (1 mL) at 30 °C in open flask. To the resulting mixture, MeOH (108 μL, 2.67 mmol) was added. The reaction mixture was stirred for 20 h at 30 °C. The resulting mixture was slowly quenched with saturated aqueous $NaHCO_3$ at 0 °C. The aqueous layer was extracted three times with $CHCl_3$. The combined organic layer was dried over $MgSO_4$, and concentrated under reduced pressure. To the crude mixture, TMSCN (533 μL, 5.34 mmol) was added at room temperature under Ar atmosphere. The reaction mixture was stirred for 30 min at room temperature. CH_2Cl_2 was added to the resulting mixture and cooled at –20 °C under Ar atmosphere. To the resulting mixture, $BF_3·Et_2O$ (135 μL, 1.07 mmol) was slowly added at – 20 °C. The reaction mixture was stirred for 1.5 h at – 20 °C under Ar atmosphere before being quenched with saturated aqueous $NaHCO_3$. The aqueous layer was extracted three times with EtOAc. The combined organic layer was washed with saturated aqueous NaCl solution, dried over $MgSO_4$, and concentrated under reduced pressure. Flash chromatography (SiO_2, 20% EtOAc / *n*-hexane) provided thiolactam **11** (508 mg, 90% as three diastereomers mixture; dr = 5 : 3 : 2) as a pale yellow oil.

Compound **11** (three diastereomer mixture)

^{1}H NMR (500MHz, $CDCl_3$) δ 7.58–7.67 (m), 7.34–7.46 (m), 7.29 (d, *J* = 9.0 Hz), 6.46–6.50 (m), 6.21 (d, *J* = 14.5 Hz), 6.11 (d, *J* = 14.5 Hz), 6.06 (d, *J* = 14.5 Hz), 4.69 (dd, *J* = 15.0, 1.5 Hz), 4.56–4.63 (m), 4.32 (t, *J* = 6.0 Hz), 4.26 (d, *J* = 6.0 Hz), 3.90 (d, *J* = 8.0 Hz), 3.85 (s), 3.84 (s), 3.82 (s), 3.82 (s), 3.81 (s), 3.78 (s), 3.77 (s), 3.70–3.80 (m), 3.70 (s), 2.68-2.81 (m), 2.39–2.50 (m), 2.22–2.28 (m), 2.12 (d, *J* = 13.5 Hz), 1.90 (dt, *J* = 13.5, 6.5 Hz), 1.73–1.79 (m), 1.62–1.72 (m), 1.36–1.43 (m), 1.03–1.06 (m); ^{13}C NMR (125MHz, $CDCl_3$) δ 197.7, 197.0 (2C), 191.8, 170.9, 170.6, 170.4, 161.7, 161.6, 161.5, 159.4, 159.2, 150.8, 135.9 (3C), 133.7 (2C), 133.6 (3C), 132.4, 131.8, 130.5, 130.2, 128.2, 128.1, 117.1, 116.9, 116.6, 114.5, 114.4, 114.3, 105.1, 105.0, 99.0 (2C), 91.8, 63.8, 62.3, 61.0, 60.9, 60.3, 55.9 (2C), 53.2, 53.0, 52.9, 51.0, 50.8, 49.8, 47.7, 36.3,36.2, 35.0, 31.8, 31.3, 31.0, 30.1, 30.0, 28.3, 27.3, 27.2 (2C), 19.6, 19.5 (2C); IR (neat) ν_{max} 3070, 2931, 2856, 1732, 1612, 1587, 1508, 1463, 1427, 1290, 1207, 1157, 1109, 1033, 937, 821, 736 cm^{-1}; HRMS (ESI) [M+H]$^+$ calculated for $[C_{35}H_{43}N_2O_5SSi]^+$: 631.2656 found : 631.2659.

Imidate formation

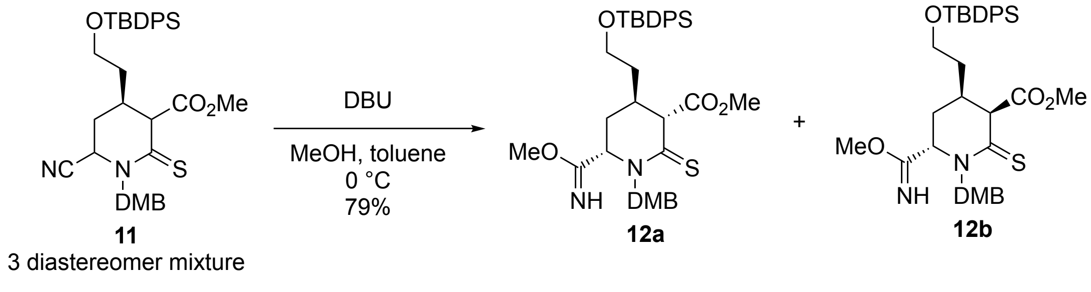

To a solution of diastereomer mixture of **11** (100 mg, dr = 5 : 3 : 2, 0.159 mmol) in toluene (159 μL) and MeOH (634 μL), 1,8-diazabicyclo[5.4.0]undec-7-ene (DBU, 47.4 mL, 0.317 mmol) was added at 0 °C under

Ar atmosphere. The reaction mixture was stirred for 22 h at 0 °C under Ar atmosphere before being quenched with aqueous NH_4Cl. The aqueous layer was extracted three times with EtOAc. The combined organic layer was washed with saturated aqueous NaCl solution, dried over $MgSO_4$, and concentrated under reduced pressure. Flash chromatography (SiO_2, 30–50% EtOAc / *n*-hexane gradient) provided imidate **12** (82.2 mg, 79% as two major diastereomers mixture; **12a** : **12b** = 3 : 1) as a pale yellow oil. The diastereomer mixture was partially separated by PTLC (35% EtOAc / *n*-hexane). The relative stereochemistry of partially isolated **12a** and **12b** were determined by coupling constant in ^{1}H-NMR. In addition, enantiomeric excess of **12a** and **12b** were determined by HPLC with DAICEL Chiralpak IC (each 94% *ee*). For **12a**: 10% *i*-PrOH/*n*-hexane, 0.5 mL/min; major enantiomer t_R = 53.2 min, minor enantiomer t_R = 43.9 min (see page S18); For **12b**: 20% *i*-PrOH/*n*-hexane, 0.75 mL/min; major enantiomer t_R = 13.7 min, minor enantiomer t_R = 36.0 min (see page S20).

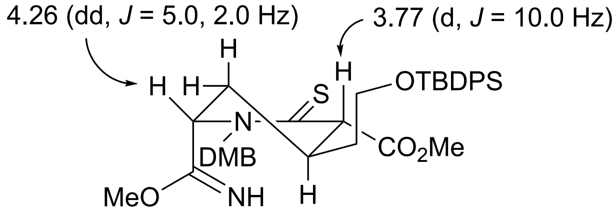

12a

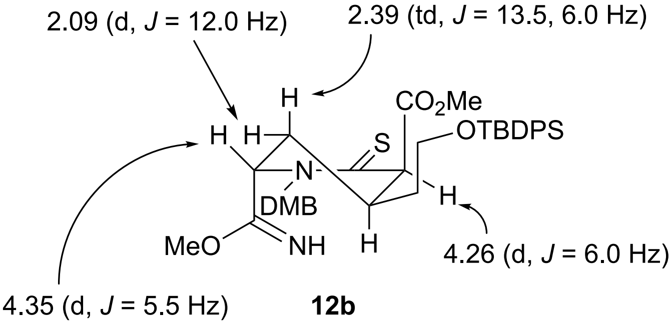

12b

<u>Imidate **12a**</u>

^{1}H NMR (500MHz, $CDCl_3$) δ 7.75 (br s, 1H), 7.59–7.61 (m, 4H), 7.36–7.44 (m, 6H), 7.28 (d, *J* = 9.5 Hz, 1H), 6.43–6.44 (m, 2H), 5.96 (d, *J* = 14.5 Hz, 1H), 4.47 (d, *J* = 14.5 Hz, 1H), 4.26 (dd, *J* = 5.0, 2.0 Hz, 1H), 3.80 (s, 3H), 3.79 (s, 3H), 3.77 (d, *J* = 10.0 Hz, 1H), 3.76 (s, 3H), 3.73 (s, 3H), 3.61–3.65 (m, 2H), 2.33–2.36 (m, 2H), 1.74–1.76 (m, 1H), 1.67 (br s, 1H), 1.59 (dt, *J* = 6.5, 13.0 Hz, 1H), 1.39–1.41 (m, 1H), 1.01 (s, 9H); ^{13}C NMR (125MHz, $CDCl_3$) δ 197.4, 171.3, 168.9, 161.0, 158.9, 135.6, 135.5, 133.6, 133.4, 129.8, 127.8, 114.9, 104.5, 98.5, 63.7, 61.0, 60.7, 55.5, 55.4, 53.9, 52.9, 51.1, 36.6, 30.6, 26.9, 19.2; IR (neat) ν_{max} 2949, 2856, 1737, 1662, 1612, 1587, 1508, 1427, 1207, 1109, 1033, 970, 821, 750 cm^{-1}; HRMS (ESI) $[M+H]^+$ calculated for $[C_{36}H_{47}N_2O_6SSi]^+$: 663.2919 found : 663.2902; $[\alpha]^{27}_D$ +103.5 (*c* 2.92, $CHCl_3$).

<u>Imidate **12b**</u>

^{1}H NMR (500MHz, $CDCl_3$) δ 7.64–7.67 (m, 4H), 7.40–7.49 (m, 6H), 7.31, (d, *J* = 8.5 Hz, 1H), 6.52 (dd, *J* = 8.5, 2.5 Hz, 1H), 6.49 (d, *J* = 2.0 Hz, 1H), 6.18 (d, *J* = 15.5 Hz, 1H), 4.35 (d, *J* = 5.5 Hz, 1H), 4.30 (d, *J* =15.5 Hz, 1H), 4.26 (d, *J* = 6.0 Hz, 1H), 3.83 (s, 3H), 3.74 (s, 3H), 3.68 (s, 3H), 3.60–3.80 (m, 5H), 2.46 (m, 1H), 2.39 (td, *J* = 13.5, 6.0 Hz, 1H), 2.09 (d, *J* = 12.0 Hz, 1H), 1.57–1.63 (m, 1H), 1.30–1.38 (m, 1H), 1.06 (s, 9H); ^{13}C NMR (125MHz, $CDCl_3$) δ 197.6, 170.8, 160.9, 161.0, 158.9, 135.9, 135.8, 133.9, 133.7, 130.2, 130.1, 129.3, 128.2, 128.1, 115.0,

104.7, 98.9, 62.5, 60.9, 60.5, 55.8, 55.7, 52.7, 51.6, 35.4, 28.5, 27.9, 27.2, 19.5; IR (neat) ν_{max} 2931, 2856, 1732, 1652, 1614, 1589, 1508, 1456, 1427, 1207, 1157, 1109, 1033, 821, 750 cm^{-1}; HRMS (ESI) $[M+H]^+$ calculated for $[C_{36}H_{47}N_2O_6SSi]^+$: 663.2919 found : 663.2908; $[\alpha]^{27}_{D}$ +92.3 (*c* 1.34, $CHCl_3$).

Reduction of thiocarbonyl group

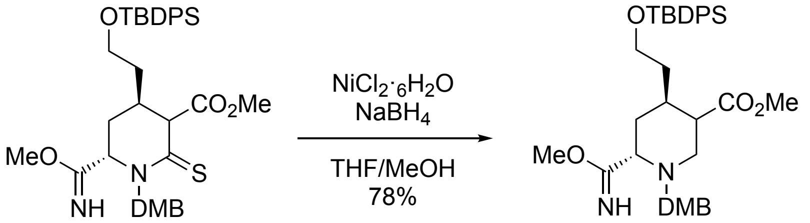

12 **13**

α-CO_2Me (**12a**) : β-CO_2Me (**12b**) = 3 : 1 α-CO_2Me (**13a**) : β-CO_2Me (**13b**) = 3 : 1

To a solution of **12** (**12a** : **12b** = 3 : 1, 211 mg, 0.318 mmol) in THF (5.3 mL) and MeOH (5.3 mL), $NaBH_4$ (144 mg, 3.82 mmol) was added at –20 °C under Ar atmosphere. To the resulting mixture, $NiCl_2$ (227 mg, 0.955 mmol) was added at –20 °C. The reaction mixture was stirred for 5 min before being quenched with saturated aqueous $NaHCO_3$. The yielded suspension was filtrated with Celite pad and the aqueous layer was extracted three times with $CHCl_3$. The combined organic layer was washed with saturated aqueous NaCl solution, dried over $MgSO_4$, and concentrated under reduced pressure. Flash chromatography (SiO_2, 30% EtOAc / *n*-hexane) provided compound **13** (157.1 mg, 78% as two diastereomers mixture; **13a** : **13b** = 3 : 1) as a pale yellow oil. The NMR chart of diastereomer mixture was shown in page S21.

Major piperidine **13a** which was partially isolated by PTLC

^{1}H NMR (500MHz, $CDCl_3$) δ 7.97 (br s, 1H), 7.65–7.67 (m, 4H), 7.36–7.43 (m, 6H), 7.20 (d, *J* = 8.0 Hz, 1H), 6.43–6.47 (m, 2H), 3.80 (s, 3H), 3.79 (s, 3H), 3.64–3.76 (m, 7H), 3.60 (s, 3H), 3.54 (d, *J* = 14.0 Hz, 1H), 3.21–3.23 (m, 1H), 2.99 (dd, *J* = 13.5, 8.5 Hz, 1H), 2.67 (dd, *J* = 13.5, 3.5 Hz, 1H), 2.43 (td, *J* = 4.0, 8.5 Hz, 1H), 20.7–2.11 (m, 1H), 1.42–1.53 (m, 2H), 1.04 (s, 9H); ^{13}C NMR (125MHz, $CDCl_3$, major diastereomer) δ 174.8, 173.9, 160.4, 159.1, 135.9 (2C), 134.2, 134.1, 130.9, 129.9 (2C), 128.0, 119.2, 104.3, 98.7, 61.7, 59.9, 55.7, 55.6, 53.6, 52.5, 51.8, 49.2, 44.3, 36.8, 31.2, 28.3, 27.1, 19.5; IR (neat) ν_{max} 2931, 2854, 1732, 1651, 1612, 1587, 1506, 1427, 1290, 1207, 1155, 1105, 1035, 821, 752 cm^{-1}; HRMS (ESI) $[M+H]^+$ calculated for $[C_{36}H_{49}N_2O_6Si]^+$: 633.3554 found : 633.3341; $[\alpha]^{26}_{D}$ –4.7 (*c* 5.35, $CHCl_3$).

DIBAL reduction, isomerization, hydrolysis of imidate

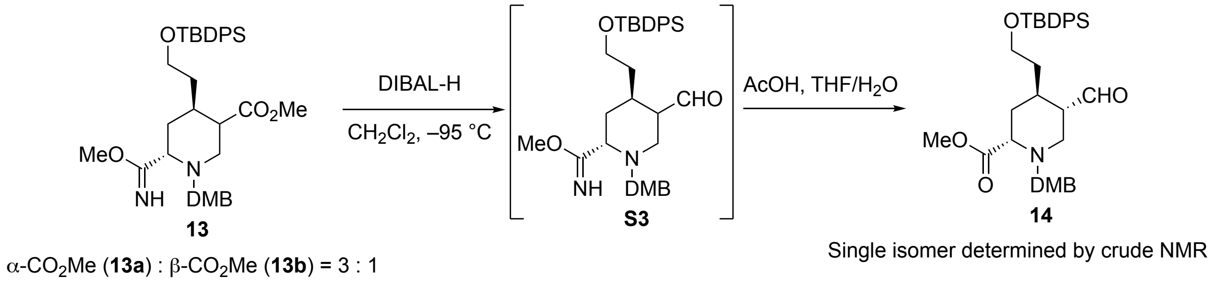

To a solution of **13** (265 mg, 0.419 mmol, **13a** : **13b** = 3 : 1) in CH_2Cl_2 (10 mL), DIBAL-H (1.0 M solution in *n*-hexane, 2.1 mL, 2.1 mmol) was added slowly at –95 °C under Ar atmosphere. The reaction mixture was stirred for 2 h before being quenched with dry MeOH (1.5 mL) at –95 °C. To the resulting mixture, saturated aqueous Rochelle's salt was added at room temperature. The aqueous layer was extracted six times with $CHCl_3$. The combined organic layer was washed with saturated aqueous NaCl solution, dried over $MgSO_4$, and concentrated under reduced pressure. The crude materials of **S3** was directly employed next hydrolysis.

To a solution of the crude materials of aldehyde **S3** in THF (4.2 mL), acetic acid (0.14 mL) and H_2O (0.84 mL) were added via syringe at room temperature under Ar atmosphere. The reaction mixture was stirred for 5 h at room temperature before being quenched with saturated aqueous $NaHCO_3$. The aqueous layer was extracted four times with EtOAc. The combined organic layer was washed with saturated aqueous NaCl solution, dried over $MgSO_4$, and concentrated under reduced pressure. Aldehyde **14** was unstable in silica gel column chromatography, thus, the crude materials of **14** was directly employed to next Tebbe olefination. The ^{1}H-NMR of the crude materials of **14** strongly indicated isomerization reaction on acid hydrolysis condition to thermodynamically stable **14** (see page S23).

Tebbe olefination

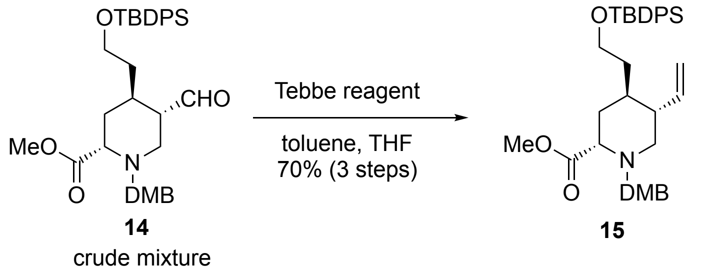

To a solution of the crude materials of **14** in dry toluene (4.2 mL) and dry THF (1.4 mL), μ-chlorobis(η^5-cyclopentadienyl)(dimethylaluminum)-μ-methylenetitanium (Tebbe reagent, 0.5 M solution in toluene, 0.85 mL, 0.461 mmol) was added at 0 °C under Ar atmosphere. The reaction mixture was stirred for 2 h at room temperature before being quenched with saturated aqueous Rochelle's salt at 0 °C. The aqueous layer was extracted three times with EtOAc. The combined organic layer was washed with saturated aqueous NaCl solution, dried over $MgSO_4$, and concentrated under reduced pressure. Flash chromatography (SiO_2, 10%

EtOAc / *n*-hexane) provided compound **15** (176 mg, 70%) as a pale yellow oil.

Piperidine **15** (single isomer)

^{1}H NMR (500MHz, $CDCl_3$) δ 7.64–7.66 (m, 4H), 7.36–7.42 (m, 6H), 7.22 (d, J = 8.5 Hz, 1H), 6.43–6.47 (m, 2H), 5.51–5.59 (m, 1H), 4.98–5.01 (m, 2H), 3.80 (s, 3H), 3.77 (s, 3H), 3.65–3.80 (m, 3H), 3.66 (s, 6H), 3.52–3.62 (m, 1H), 3.01(t, J = 11.5 Hz, 1H), 2.62 (dd, J = 12.0, 4.5 Hz, 1H), 2.13–2.15 (m, 1H), 1.93–2.00 (m, 1H), 1.84–1.90 (m, 1H), 1.48–1.51 (m, 2H), 1.15–1.21 (m, 1H), 1.02–1.04 (m, 10H); ^{13}C NMR (125MHz, $CDCl_3$) δ; 174.2, 159.9, 158.9, 40.4, 135.7, 134.1, 134.0, 130.1, 129.6, 119.9,116.2, 104.1, 98.5, 61.7, 59.9, 58.5, 55.5, 55.4, 52.9,52.8,51.0, 47.1, 36.3,33.2, 32.6, 26.9, 19.3, 18.5; IR (neat) ν_{max} 2929, 2856, 1734, 1612, 1587, 1506, 1563, 1427, 1292, 1207, 1155, 1109, 1037, 1004, 918, 821, 754 cm^{-1}; HRMS (ESI) $[M+H]^+$ calculated for $[C_{36}H_{48}NO_5Si]^+$: 602.3296 found : 602.3284; $[\alpha]^{27}_D$ –6.5 (*c* 1.29, $CHCl_3$).

DIBAL reduction and isomerization

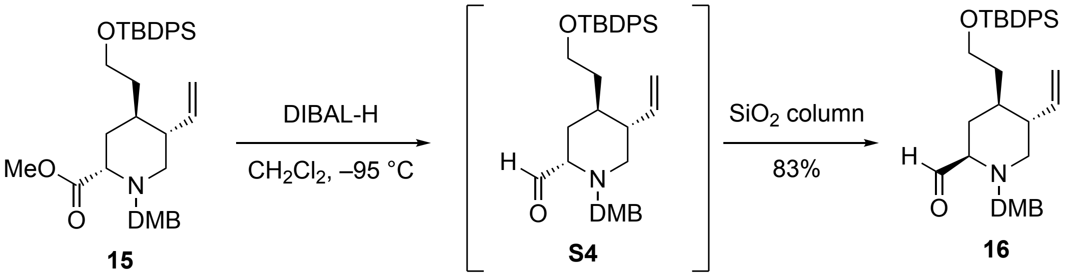

To a solution of ester **15** (1.05 g, 1.75 mmol) in CH_2Cl_2 (25 mL), DIBAL-H (1.0 M solution in *n*-hexane, 5.2 mL, 5.2 mmol) was added slowly at –95 °C under Ar atmosphere. The reaction mixture was stirred for 2.5 h before being quenched with EtOAc (3.6 mL) followed by addition of MeOH (3.6 mL) at –95 °C. To the resulting mixture, saturated aqueous Rochelle's salt was added at room temperature. The aqueous layer was extracted four times with $CHCl_3$. The combined organic layer was washed with saturated aqueous NaCl solution, dried over $MgSO_4$, and concentrated under reduced pressure. Flash chromatography (SiO_2, 1% Et_3N/ 20%EtOAc–*n*-hexane solution) provided compound **16** (826 mg, 83%) as a colorless oil. The ^{1}H-NMR of crude materials indicated **S4**. After SiO_2 column chromatography, C6 aldehyde was isomerized to thermodynamically stable form. As a result, **16** was obtained as single isomer. ^{1}H-NMR of the crude mixture; See page S25. ^{1}H-NMR of isolated **16**; see page S26.

Piperidine-2-carbaldehyde **16**

^{1}H NMR (500MHz, $CDCl_3$) δ 9.47 (s, 1H), 7.62–7.64 (m, 4H), 7.36–7.42 (m, 6H), 7.15 (d, J = 8.0 Hz, 1H), 6.43–6.46 (m, 2H), 5.41–5.48 (m, 1H), 4.98–5.02 (m, 2H), 3.80 (s, 3H), 3.77 (s, 3H), 3.61–3.68 (m, 3H), 3.58 (d, J =14.0 Hz, 1H), 3.45 (d, J = 13.5 Hz, H), 2.85 (dd, J = 11.5, 3.5 Hz, 1H), 2.68–2.72 (m, 1H), 1.83–1.97 (m, 3H), 1.64–1.68 (m, 1H), 1.27–1.36 (m, 2H), 1.14–1.21 (m, 1H); ^{13}C NMR (125MHz, $CDCl_3$) δ; 204.1, 159.1, 139.6, 135.8, 135.7,

134.0, 132.2, 130.9, 129.8, 127.9, 116.9, 104.0, 98.1, 70.7, 61.6, 57.2, 55.4, 54.6, 52.9, 46.5, 36.7, 36.2, 35.7, 31.9, 29.9, 27.0, 19.3; IR (neat) ν_{max} 2929, 2856, 1730, 1614, 1506, 1463, 1427, 1290, 1263, 1209, 1157, 1110, 1039, 920, 823, 738, 702 cm^{-1}; HRMS (ESI) $[M+H]^+$ calculated for $[C_{35}H_{46}NO_4Si]^+$: 572.3191 found : 572.3177; $[\alpha]^{26}_D$ –6.4 (*c* 0.93, $CHCl_3$).

Coupling reaction with dihydroquinoline derivative **17**

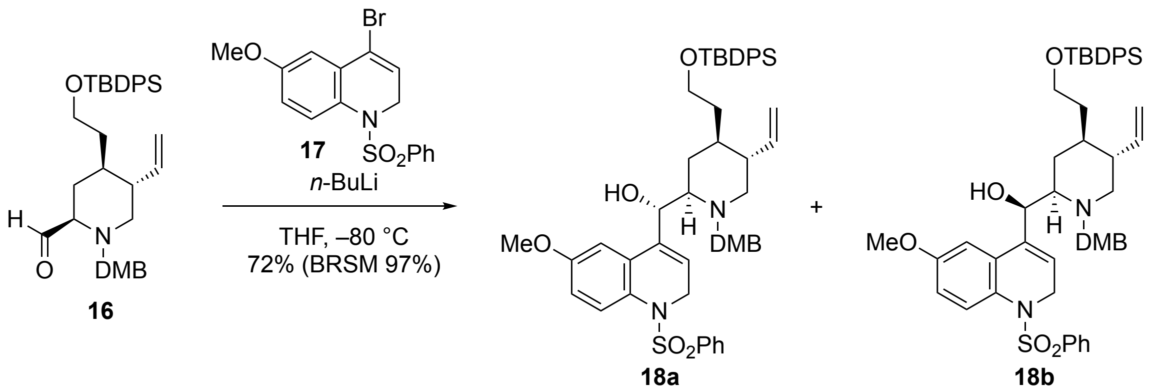

To a solution of dihydroquinoline **17**[S2)] (79.9 mg, 0.21 mmol) in dry THF (1 mL), *n*-BuLi (2.5M solution in *n*-hexane, 84 μL, 0.21 mmol) was slowly added at –90 °C under Ar atmosphere. The reaction mixture was stirred for 30 min at –90 °C. To the resulting mixture, a solution of aldehyde **16** (100 mg, 0.175 mmol) in dry THF (1 mL) was slowly added at –90 °C under Ar atmosphere. The reaction mixture was stirred for 22 h at –80 °C before being quenched with benzoic acid (26 mg, 0.21 mmol). To the resulting mixture, excess amount of 20% aqueous NH_3 solution was added at 0 °C. The aqueous layer was extracted four times with EtOAc. The combined organic layer was washed with H_2O, dried over $MgSO_4$, and concentrated under reduced pressure. Flash chromatography (SiO_2, 1% Et_3N/ 20%EtOAc–*n*-hexane solution to 1% Et_3N/ 90%EtOAc–*n*-hexane solution gradient) provided compound **19** (110.1 mg, 72%, **18a** : **18b** = 1 : 1) as a pale yellow oil and 25.8 mg of **16** was recovered (25%). The diastereomer mixture (**18a** and **18b**) was partially separated by PTLC (35% EtOAc / *n*-hexane).

Coupling product **18a**

^{1}H NMR (500MHz, C_6D_6, VT 70 °C) δ 7.85 (d, *J* = 9.0 Hz, 1H), 7.75–7.77 (m, 4H), 7.62 (d, *J* = 9.0 Hz, 1H), 7.23–7.27 (m, 6H), 6.95 (d, *J* = 7.5 Hz, 1H), 6.89 (t, *J* = 7.5 Hz, 1H), 6.63 (d, *J* = 9.5 Hz, 1H), 6.35–6.38 (m, 2H), 5.92 (br s, 1H), 5.26–5.35 (m, 1H), 4.85–4.89 (m, 2H), 4.35–4.39 (m, 2H), 4.24 (d, *J* = 18.0 Hz, 1H), 3.68–3.77 (m, 3H), 3.37–3.40 (m, 9H), 2.94 (d, *J* = 9.5 Hz, 1H), 2.05–2.18 (m, 2H),1.83–1.87 (m, 1H), 1.43 (d, *J* = 11.6 Hz, 1H), 1.26–1.30 (m, 3H), 1.18–1.25 (m, 2H), 1.15 (s, 9H); ^{13}C NMR (125MHz, C_6D_6, VT 70 °C) δ; 159.6, 158.6, 141.6, 136.2, 134.9, 132.4, 131.6, 130.2, 129.8, 122.8 (br), 116.5 (br), 112.9, 111.8, 105.0, 99.7, 70.8 (br), 65.8, 62.5, 55.4, 55.3, 55.2, 45.8, 42.3 (br), 37.9 (br), 37.6, 30.3 (br), 27.5, 19.7, several aromatic carbons were overlapped to C_6D_6; IR (neat) ν_{max} 2929, 2854, 1735, 1612, 1508, 1427, 1352, 1290, 1242, 1209, 1159, 1089, 1037, 918, 821, 727 cm^{-1};

HRMS (ESI) $[M+H]^+$ calculated for $[C_{51}H_{61}N_2O_7SSi]^+$: 873.3963 found : 873.3944; $[\alpha]^{26}_D$ –4.1 (*c* 0.76, $CHCl_3$).

Coupling product **18b**

^{1}H NMR (500MHz, $CDCl_3$) δ 7.51–7.53 (m, 4H), 7.39 (d, *J* = 7.5 Hz, 2H), 7.29–7.32 (m, 3H), 7.22–7.25 (m, 3H), 6.95 (br s, 1H), 6.82 (br s, 1H), 6.71 (dd, *J* = 9.5, 3.5 Hz, 1H), 6.33–6.36 (m, 2H), 5.83 (br s, 1H), 5.20–5.27 (m, 1H), 4.82–4.85 8m, 2H), 4.39 (d, *J* = 18.0 Hz, 1H), 4.17–4.22 (m, 2H), 3.71 (s, 6H), 3.64 (s, 3H), 3.48–3.58 (m, 4H), 2.65 (d, *J* = 14.5 Hz, 1H), 2.50 (br s, 1H), 1.92–1.97 (m, 1H), 1.11–1.24 (m, 5H), 0.91 (s, 9H); ^{13}C NMR (125MHz, $CDCl_3$) δ 158.8, 157.8, 140.1, 135.6, 134.0, 132.7, 131.9, 130.5, 129.9, 129.7, 129.1, 128.9, 128.2, 127.8, 127.4, 122.8 (br), 116.5 (br), 112.2, 110.7, 104.0, 98.8, 70.8 (br), 65.4, 64.9, 61.7, 55.5, 55.4, 46.1(br), 45.3, 41.0, 37.3, 36.8 (br), 29.8 (br), 27.0, 19.2; IR (neat) ν_{max} 2966, 2860, 1716, 1456, 1033, 871, 773 cm^{-1}; HRMS (ESI) $[M+H]^+$ calculated for $[C_{51}H_{61}N_2O_7SSi]^+$: 873.3963 found : 873.3970; $[\alpha]^{26}_D$ +7.6 (*c* 0.05, $CHCl_3$).

Optimization to complete total synthesis of (+)-quinine using isolated 19a

Acetylation and removal of TBDPS group

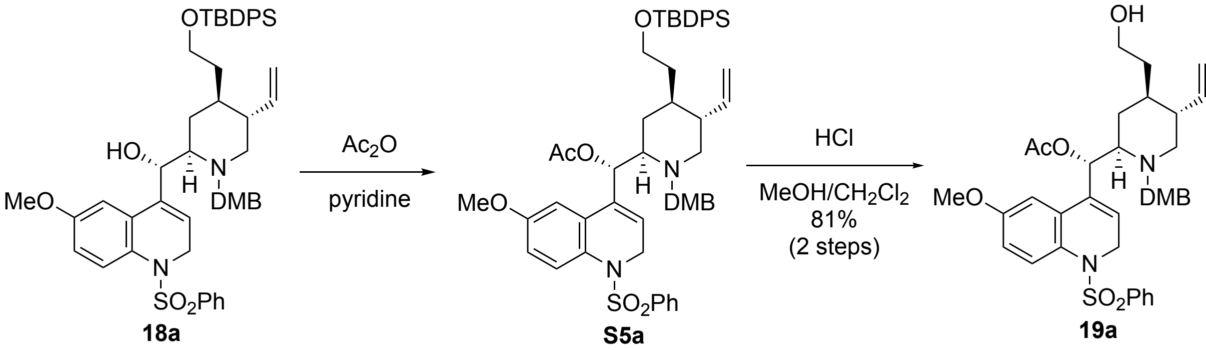

To a solution of **18a** (21.8 mg, 0.0259mmol) in pyridine (0.5 mL), acetic anhydride (5.5 µL, 0.059 mmol) and dimethylaminopyridine (DMAP, 0.2 mg, 0.00016 mmol) were added at 0 °C under Ar atmosphere. The reaction mixture was stirred for 68 h at room temperature before being quenched with saturated aqueous $NaHCO_3$. The aqueous layer was extracted three times with $CHCl_3$. The combined organic layer was washed with H_2O, dried over $MgSO_4$, and concentrated under reduced pressure. The obtained crude material was directly employed to next reaction.

To a solution of the crude mixture of **S5a** in CH_2Cl_2 (160 µL) and MeOH (160 µL), 2M HCl/MeOH (82 µL, 0.16 mmol) was slowly added at 0 °C under Ar atmosphere. The reaction mixture was stirred for 6 h at room temperature before being quenched with saturated aqueous $NaHCO_3$. The aqueous layer was extracted three times with $CHCl_3$. The combined organic layer was washed with saturated aqueous NaCl, dried over $MgSO_4$, and concentrated under reduced pressure. Flash chromatography (SiO_2, 40%EtOAc–*n*-hexane) provided compound **19a** (13.7 mg, 81%) as a pale yellow oil.

Compound **19a**

^{1}H NMR (500MHz, $CDCl_3$) δ 7.63 (d, *J* = 7.5 Hz, 2H), 7.53 (t, *J* = 7.0 Hz, 1H), 7.40 (t, *J* = 7.5 Hz, 2H), 7.24–7.27 (m, 1H, overlapped to $CDCl_3$), 7.20 (d, *J* = 8.0 Hz, 1H), 6.94 (s, 1H), 6.78 (dd, *J* = 8.5, 3.0 Hz, 1H), 6.43–6.45 (m, 2H), 6.25 (s, 1H), 5.93 (s, 1H), 5.40–5.47 (m, 1H), 4.96–4.99 (m, 2H), 4.50 (dd, *J* = 17.5, 4.5 Hz, 1H), 4.22 (d, *J* = 15.5 Hz, 1H), 4.14 (d, *J* = 14.0 Hz, 1H), 3.81 (s, 3H), 3.80 (s, 3H), 3.78 (s, 3H), 3.63–3.69 (m, 2H), 3.22 (d, *J* = 13.5 Hz, 1H), 2.82 (d, *J* = 9.0 Hz, 1H), 2.38 (d, *J* = 10.5 Hz, 1H), 2.05 (s, 3H), 1.76–1.91 (m, 3H), 1.14–1.25 (m, 3H); ^{13}C NMR (125MHz, $CDCl_3$) δ; 169.5, 159.9, 158.6, 158.3, 140.1, 139.7, 134.0, 133.1, 131.0, 130.1,.129.0 (2C), 128.2, 127.6, 127.5, 122.5, 119.4, 116.5, 113.4, 109.3, 104.3, 98.5, 77.4 (2C), 77.2, 76.9, 70.7, 69.8, 63.5, 60.0, 59.2, 55.6, 55.5, 50.4, 45.9, 45.0, 36.8, 36.4, 29.9, 29.8, 29.5, 29.4, 28.9, 21.3; IR (neat) ν_{max} 2922, 2845, 1743, 1508, 1456, 1163, 1033 cm^{-1}; HRMS (ESI) $[M+H]^+$ calculated for $[C_{37}H_{45}N_2O_8S]^+$: 677.2891 found : 677.2895; $[\alpha]^{27}_D$ +28.4(*c* 0.28, $CHCl_3$).

Quinuclidine formation and complete total synthesis of (+)-quinine

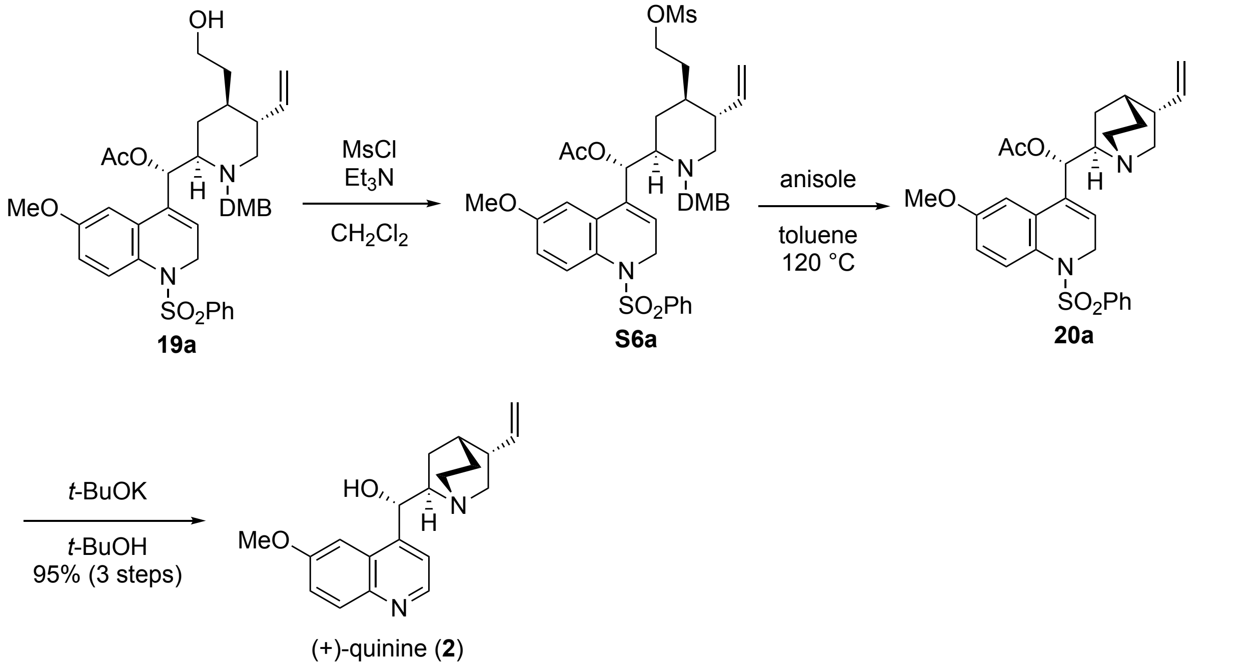

To a solution of **19a** (9.5 mg, 0.014 mmol) and Et_3N (20 μL, 0.14 mmol) in CH_2Cl_2 (0.3 mL), methanesulfonyl chloride (MsCl, 4.4 μL, 0.056 mmol) was added at 0 °C under Ar atmosphere. The reaction mixture was stirred for 20 min at 0 °C before being quenched with 20% aqueous NH_3 solution. The aqueous layer was extracted three times with EtOAc. The combined organic layer was washed with saturated aqueous NaCl, dried over $MgSO_4$, and concentrated under reduced pressure. The crude materials of **S6a** was directly employed to the next quinuclidine formation.

To a solution of the crude materials of **S6a** in toluene (0.4 mL), anisole (4.9 μL, 0.042 mmol) was added at room temperature under Ar atmosphere. The reaction mixture was refluxed for 21 h at 120 °C before being quenched with 20% aqueous NH_3 solution. The aqueous layer was extracted four times with EtOAc. The

combined organic layer was washed with saturated aqueous NaCl, dried over $MgSO_4$, and concentrated under reduced pressure. The crude materials of **20a** was directly employed to the next reaction.

To a solution of the crude materials of **20a** in *t*-BuOH (0.6 mL), *t*-BuOK (7.6 mg, 0.33 mmol) was added at room temperature under Ar atmosphere. The reaction mixture was stirred for 3 h at 60 °C before being quenched with 20% aqueous NH_3 solution. The aqueous layer was extracted four times with EtOAc. The combined organic layer was washed with H_2O, dried over $MgSO_4$, and concentrated under reduced pressure. Flash chromatography (SiO_2–NH, 1%MeOH–$CHCl_3$) provided (+)-quinine (**2**, 4.3 mg, 95%) as white amorphous powder. All spectral data of (+)-quinine (**2**) except sign of rotation were identified with authentic (–)-quinine; see page S31, 32.

Unnatural (+)-quinine (**2**)

^{1}H NMR (500MHz, $CDCl_3$) δ 8.58 (d, *J* = 5.0 Hz, 1H), 7.93 (d, *J* = 9.0 Hz, 1H), 7.49 (d, *J* = 4.0 Hz, 1H), 7.28 (dd, *J* = 9.3, 2.8 Hz, 1H), 7.19 (d, *J* = 2.5 Hz, 1H), 5.70 (ddd, *J* = 17.0, 10.5, 7.5 Hz, 1H), 5.54 (d, *J* = 3.5 Hz, 1H), 4.93 (dt, *J* = 16.5, 1.5 Hz, 1H), 4.90 (br d, *J* = 15.5 Hz, 1H), 3.85 (s, 3H), 3.45–3.51 (m, 1H), 3.02–3.07 (m, 2H), 2.59–2.64 (m, 2H), 2.25 (br s, 1H), 1.70–1.79 (m, 3H), 1.45–1.50 (m, 2H); ^{13}C NMR (125MHz, $CDCl_3$) δ; 158.2, 148.3, 147.9, 144.5, 142.3, 131.9, 126.7, 122.0, 118.9, 114.9, 101.7, 73.3, 60.3, 57.4, 56.1, 43.6, 40.4, 28.3, 28.0, 21.9; IR (neat) ν_{max} 2935, 2862, 1622, 1508, 1240, 1031, 717 cm^{-1}; HRMS (ESI) $[M+H]^+$ calculated for $[C_{20}H_{25}N_2O_2]^+$: 325.1911 found : 325.1902; $[\alpha]^{26}_D$ +142.2 (*c* 1.18, EtOH), [natural (–)-quinine $[\alpha]^{25}_D$ –150.0 (*c* 1.38, EtOH)].

Total syntheses of (+)-quinine and (–)-9-*epi*-quinine

Acetylation and removal of TBDPS group

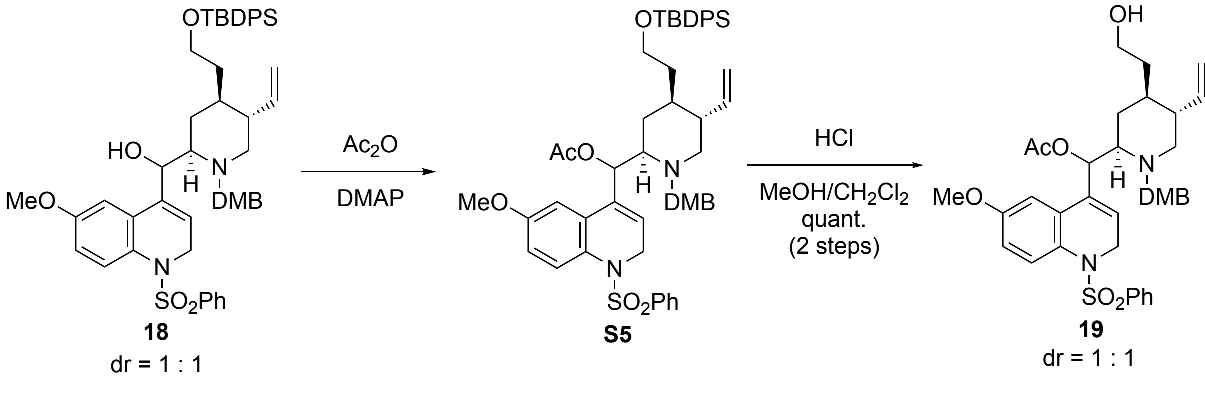

To a solution of **18** (9α-OH : 9β-OH = 1 : 1, 2.10 g, 2.04 mmol) in acetic anhydride (24 mL), dimethylaminopyridine (DMAP, 58.8 mg, 0.48 mmol) was added at 0 °C under Ar atmosphere. The reaction mixture was stirred for 30 min at room temperature before being quenched with 15% aqueous NaOH solution at 0 °C. The aqueous layer was extracted three times with $CHCl_3$. The combined organic layer was washed with H_2O, dried over $MgSO_4$, and concentrated under reduced pressure. The crude materials of **S5** was directly employed to next removal of TBDPS group.

To a solution of the crude mixture of **S5** in CH_2Cl_2 (24 mL) and MeOH (24 mL), 2M HCl/MeOH (6.0 mL, 10.2 mmol) was slowly added at 0 °C under Ar atmosphere. The reaction mixture was stirred for 12 h at room temperature before being quenched with saturated aqueous $NaHCO_3$ at 0 °C. The aqueous layer was extracted three times with $CHCl_3$. The combined organic layer was washed with saturated aqueous NaCl, dried over $MgSO_4$, and concentrated under reduced pressure. Flash chromatography (SiO_2, 40%EtOAc–*n*-hexane) provided compound **19** (9α-OH : 9β-OH = 1 : 1, 1.67 g, quant.) as a pale yellow oil. The NMR chart of diastereomer mixture **19** was shown page S30.

<u>Quinuclidine formation and complete total synthesis of (+)-quinine and (–)-9-*epi*-quinine</u>

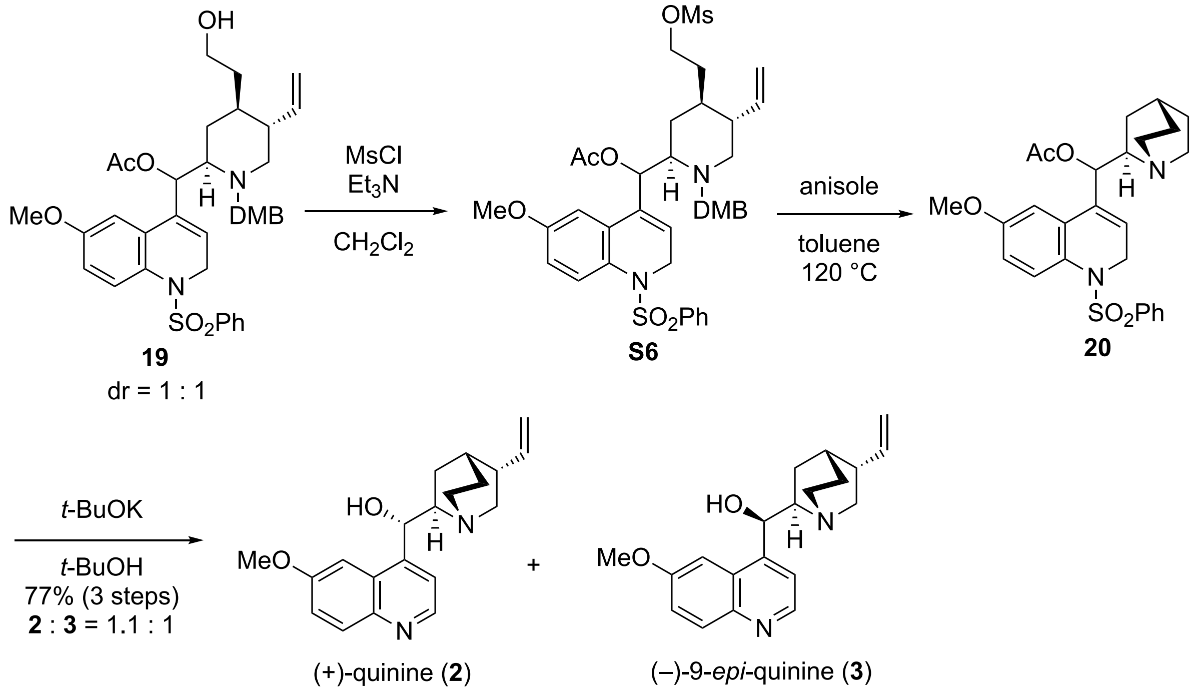

To a solution of **19** (1.59 g, 2.35 mmol) and Et_3N (1.85 mL, 11.75 mmol) in CH_2Cl_2 (29 mL), methanesulfonyl chloride (MsCl, 273 μL, 3.53 mmol) was added at 0 °C under Ar atmosphere. The reaction mixture was stirred for 5 min at 0 °C before being quenched with 20% aqueous NH_3 solution. The aqueous layer was extracted three times with EtOAc. The combined organic layer was washed with saturated aqueous NaCl, dried over $MgSO_4$, and concentrated under reduced pressure. The crude materials of **S6** was directly employed to the next quinuclidine formation.

To a solution of the crude materials of **S6** in toluene (34 mL), anisole (798 μL, 7.05 mmol) was added at room temperature under Ar atmosphere. The reaction mixture was refluxed for 7 h at 120 °C before being quenched with 20% aqueous NH_3 solution. The aqueous layer was extracted four times with EtOAc. The combined organic layer was washed with H_2O, dried over $MgSO_4$, and concentrated under reduced pressure. The crude materials of **20** was directly employed to the next reaction.

To a solution of the crude materials of **20** in *t*-BuOH (34 mL), *t*-BuOK (791 mg, 7.05 mmol) was added at room temperature under Ar atmosphere. The reaction mixture was stirred for 1.5 h at 60 °C before being

quenched with 20% aqueous NH_3 solution. The aqueous layer was extracted four times with EtOAc. The combined organic layer was washed with H_2O, dried over $MgSO_4$, and concentrated under reduced pressure. Flash chromatography (SiO_2–NH, 1–10%MeOH–$CHCl_3$) provided (+)-quinine (**2**, 310 mg, 41%) as a white amorphous powder and (–)-9-*epi*-quinine (**3**, 272 mg, 36%) as a white amorphous powder. All spectral data of (–)-9-*epi*-quinine (**3**) except sign of rotation were identified with reported data[S3)].

(–)-9-*epi*-quinine

^{1}H NMR (500MHz, $CDCl_3$) δ 8.71 (d, *J* = 4.0 Hz, 1H), 8.00 (d, *J* = 8.5 Hz, 1H), 7.63 (s, 1H), 7.35–7.39 (m, 2H), 5.72 (dt, *J* = 17.0, 9.0 Hz, 1H), 4.91–5.01 (m, 3H), 3.91 (s, 3H), 3.24 (dd, *J* = 13.0, 10.0 Hz, 1H), 3.16 (dt, *J* = 14.0, 7.5 Hz, 1H), 3.09 (q, *J* = 8.5, 1H), 2.75–2.77 (m, 2H), 2.29 (br s, 1H), 1.58–1.69 (m, 3H), 1.43 (t, *J* = 5.5, 1H), 0.93 (dd, *J* = 12.5, 7.5 Hz, 1H); ^{13}C NMR (125MHz, $CDCl_3$) δ; 157.4, 147.5, 144.7, 144.4, 141.4, 131.5, 128.1, 121.3, 120.1, 114.6, 102.5, 71.3, 61.5, 55.9, 55.4, 40.7, 39.9, 27.9, 27.2, 25.1; IR (neat) ν_{max} 2933, 2864, 1620, 1506, 1240, 1028, 852,715 cm^{-1}; HRMS (ESI) $[M+H]^+$ calculated for $[C_{20}H_{25}N_2O_2]^+$: 325.1911 found : 325.1903; $[\alpha]^{28}_D$ –29.3 (*c* 3.60, EtOH); [for (+)-9-*epi*-quinine: lit[S3b)] $[\alpha]^{22}_D$ +23 (*c* 1.0, EtOH)]

Synthetic (–)-9-*epi*-quinine<br>^{13}C-NMR<br>(125 MHz, $CDCl_3$)	Reported (+)-9-*epi*-quinine [S3b)]<br>^{13}C-NMR<br>(100 MHz, $CDCl_3$)
157.4	157.6
147.5	147.5
144.7	144.8
144.4	143.9
141.4	140.3
131.5	131.6
128.1	128.0
121.3	121.5
120.1	120.1
114.6	115.3
102.5	102.5
71.3	70.7
61.5	61.6
55.9	55.7
55.4	55.3
40.7	40.9
39.9	39.1
27.9	27.1
27.2	27.0
25.1	24.7

Mitsunobu conversion from (–)-9-*epi*-quinine to (+)-quinine

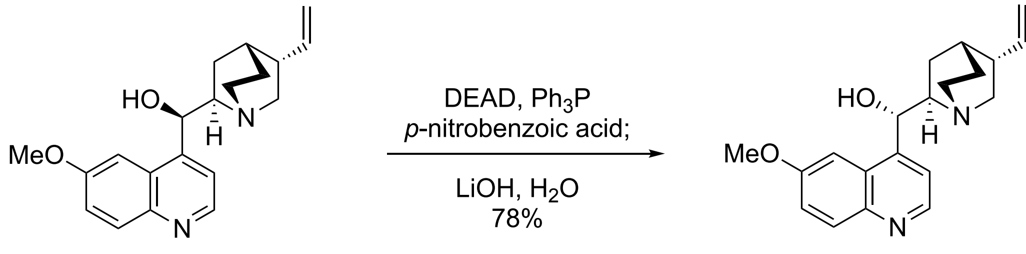

(–)-9-*epi*-quinine (**3**) (+)-quinine (**2**)

To a solution of (–)-9-*epi*-quinine (**3**, 25.5 mg, 0.079 mmol), triphenylphosphine (26.8 mg, 0.10 mmol) and *p*-nitrobenzoic acid (PNBA, 14.4 mg, 0.087 mmol) in dry THF (786 μL), diethyl azodicarboxylate (DEAD, 39.3 μL, 0.087 mmol) at 0 °C under Ar atmosphere. The reaction mixture was stirred for 7 h at room temperature. The resulting mixture was cooled at 0 °C, then aqueous 1M LiOH solution (393 μL) and MeOH (79 μL) were added to the reaction mixture. The reaction mixture was stirred for 17 h at room temperature under Ar atmosphere. The resulting mixture was extracted four times with $CHCl_3$. The combined organic layer was washed with H_2O, dried over $MgSO_4$, and concentrated under reduced pressure. Flash chromatography (SiO_2–NH, 1–10%MeOH–$CHCl_3$) provided (+)-quinine (**2**, 19.9 mg, 78%) as white amorphous powder.

Recrystallization of (+)-quinine

100 mg of (+)-quinine was dissolved to a solution of H_2SO_4 (14.8 mL, 0.28 mmol) in H_2O (5 mL). After colorless crystals were formed, the suspension was filtrated. As a result, (+)-quinine sulfate hydrate was obtained in 76% yield (92.1 mg) as colorless crystals. The part of crystals was added to 20% aqueous NH_3 solution and the resulting mixture was extracted with $CHCl_3$. The organic layer was concentrated and enantiomeric excess was determined using the crude materials. The enantiomeric excess was over 99% *ee*. DAICEL ChiralPak AS-H column; 10% *i*-PrOH/*n*-hexane, 0.25 mL/min; synthetic unnatural (+)-quinine t_R = 32.7 min, natural (–)-quinine t_R = 19.8 min (see page S34).

Reference;

S1) a) S. BouzBouz, C. Roche, J. Cossy, *Synlett* **2009**, *5*, 803–807; b) H. Zhang, X. Ma, H. Kang, R. Wang, *Chem. Asian. J.* **2013**, *8*, 542–545.

S2) a) H. Murase, K. Senda, M. Senoo, T. Hata, H. Urabe, *Chem. Eur. J.* **2014**, *20*, 317–322; b) L. Alonso-Marañón, L. A. Sarandeses, M. M. Martínez, J. P. Sestelo, *Org. Chem. Front.* **2017**, *4*, 500–505.

S3) a) T. M. Lipińska, K. Piechocka, M. Denisiuk, B. Chmiel, A. Skórska-Stania, *ARKIVOC* **2012**, 264–280; b) A. P. Gorka, K. S. Sherlach, A. C. de Dios, P. D. Roepe, *Antimicrob. Agents Chemother.* **2013***, 57, 365–364.*

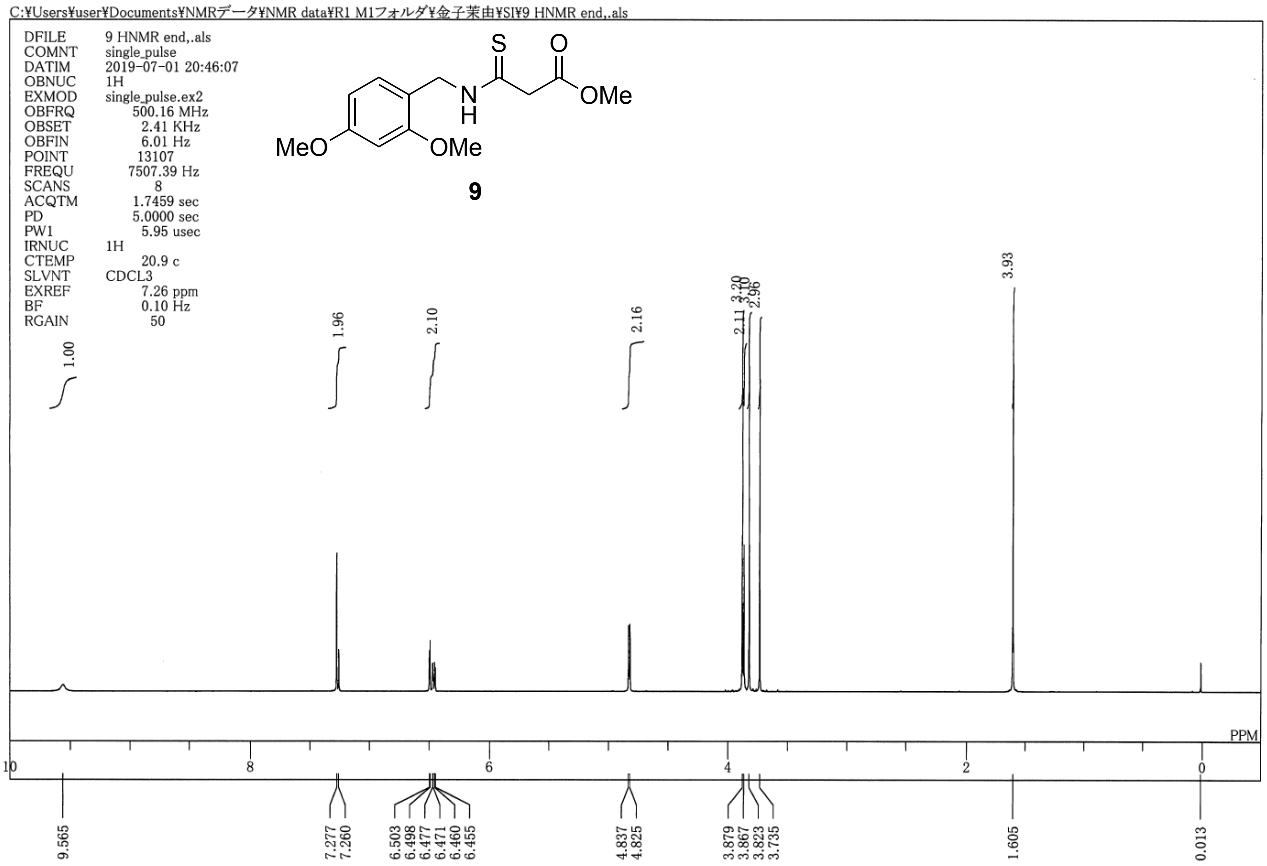
C:¥Users¥user¥Documents¥NMRデータ¥NMR data¥R1 M1フォルダ¥金子茉由¥SI¥9 HNMR end,.als
DFILE 9 HNMR end,.als
COMNT single_pulse
DATIM 2019-07-01 20:46:07
OBNUC 1H
EXMOD single_pulse.ex2
OBFRQ 500.16 MHz
OBSET 2.41 KHz
OBFIN 6.01 Hz
POINT 13107
FREQU 7507.39 Hz
SCANS 8
ACQTM 1.7459 sec
PD 5.0000 sec
PW1 5.95 usec
IRNUC 1H
CTEMP 20.9 c
SLVNT CDCL3
EXREF 7.26 ppm
BF 0.10 Hz
RGAIN 50
S
O
N
H
OMe
MeO
OMe
9
1.00
1.96
2.10
2.16
2.11
3.20
3.10
2.96
3.93
PPM
10
8
6
4
2
0
9.565
7.277
7.260
6.503
6.498
6.477
6.471
6.460
6.455
4.837
4.825
3.879
3.867
3.823
3.735
1.605
0.013

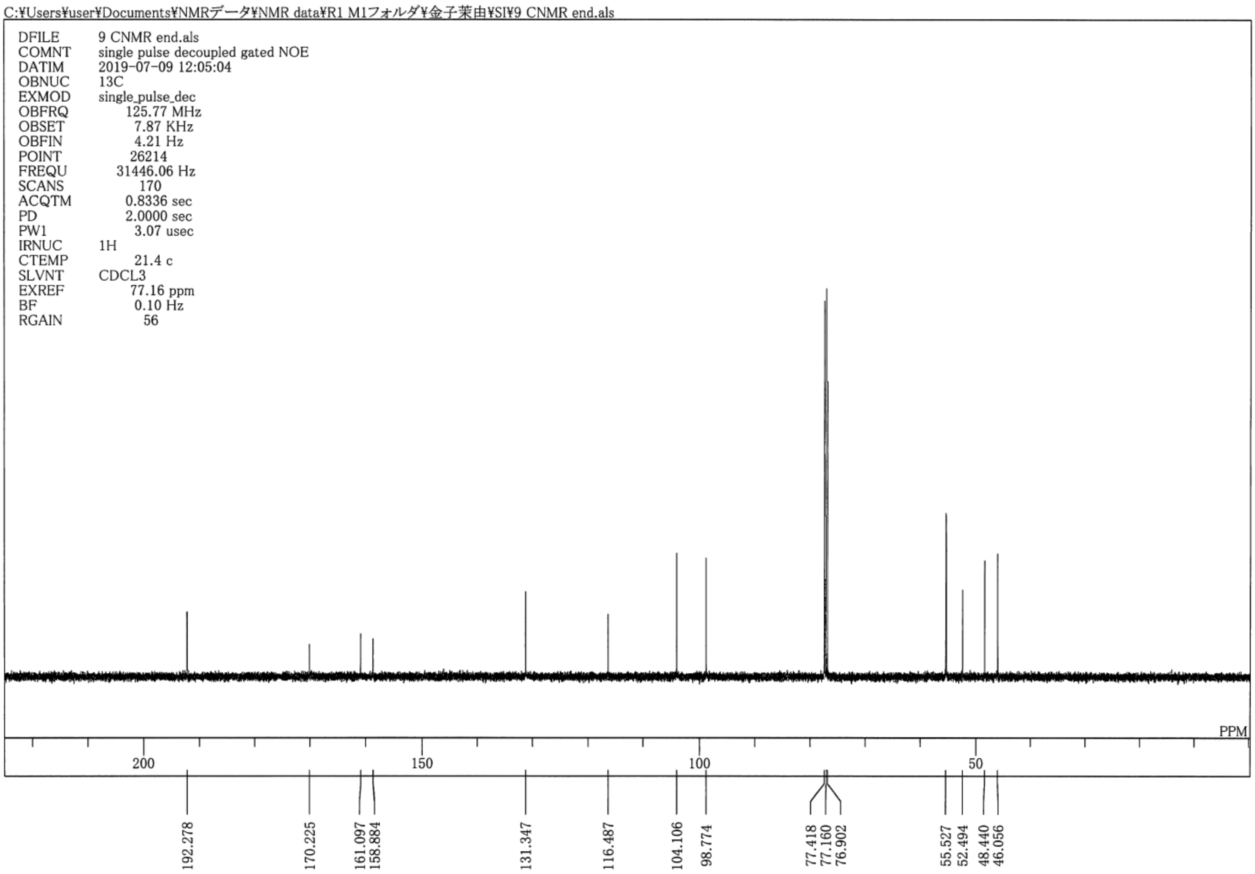
C:¥Users¥user¥Documents¥NMRデータ¥NMR data¥R1 M1フォルダ¥金子茉由¥SI¥9 CNMR end.als
DFILE 9 CNMR end.als
COMNT single pulse decoupled gated NOE
DATIM 2019-07-09 12:05:04
OBNUC 13C
EXMOD single_pulse_dec
OBFRQ 125.77 MHz
OBSET 7.87 KHz
OBFIN 4.21 Hz
POINT 26214
FREQU 31446.06 Hz
SCANS 170
ACQTM 0.8336 sec
PD 2.0000 sec
PW1 3.07 usec
IRNUC 1H
CTEMP 21.4 c
SLVNT CDCL3
EXREF 77.16 ppm
BF 0.10 Hz
RGAIN 56
PPM
200
150
100
50
192.278
170.225
161.097
158.884
131.347
116.487
104.106
98.774
77.418
77.160
76.902
55.527
52.494
48.440
46.056

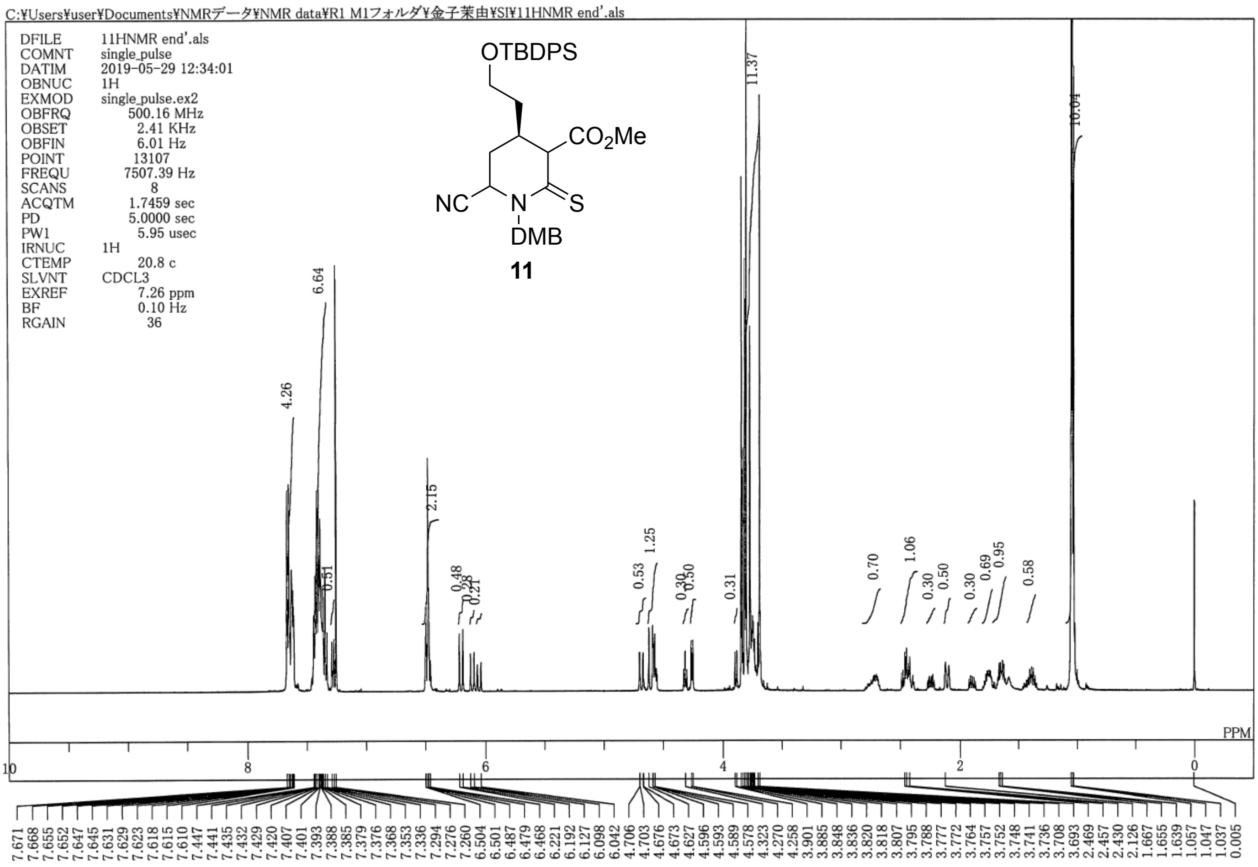
C:¥Users¥user¥Documents¥NMRデータ¥NMR data¥R1 M1フォルダ¥金子茉由¥SI¥11HNMR end'.als
DFILE 11HNMR end'.als
COMNT single_pulse
DATIM 2019-05-29 12:34:01
OBNUC 1H
EXMOD single_pulse.ex2
OBFRQ 500.16 MHz
OBSET 2.41 KHz
OBFIN 6.01 Hz
POINT 13107
FREQU 7507.39 Hz
SCANS 8
ACQTM 1.7459 sec
PD 5.0000 sec
PW1 5.95 usec
IRNUC 1H
CTEMP 20.8 c
SLVNT CDCL3
EXREF 7.26 ppm
BF 0.10 Hz
RGAIN 36
OTBDPS
CO2Me
NC
N
S
DMB
11
4.26
6.64
0.51
2.15
0.48
0.28
0.21
0.53
1.25
0.30
0.50
0.31
11.37
0.70
1.06
0.30
0.50
0.30
0.69
0.95
0.58
10.04
PPM
10
8
6
4
2
0
7.671 7.668 7.655 7.652 7.647 7.645 7.631 7.629 7.623 7.618 7.615 7.610 7.447 7.441 7.435 7.432 7.429 7.420 7.407 7.401 7.393 7.388 7.385 7.379 7.376 7.368 7.353 7.336 7.294 7.276 7.260 6.504 6.501 6.487 6.479 6.468 6.221 6.192 6.127 6.098 6.042 4.706 4.703 4.676 4.673 4.627 4.596 4.593 4.589 4.578 4.323 4.270 4.258 3.901 3.885 3.848 3.836 3.820 3.818 3.807 3.795 3.788 3.777 3.772 3.764 3.757 3.752 3.748 3.741 3.736 3.708 3.693 2.469 2.457 2.430 2.126 1.667 1.655 1.639 1.057 1.047 1.037 0.005

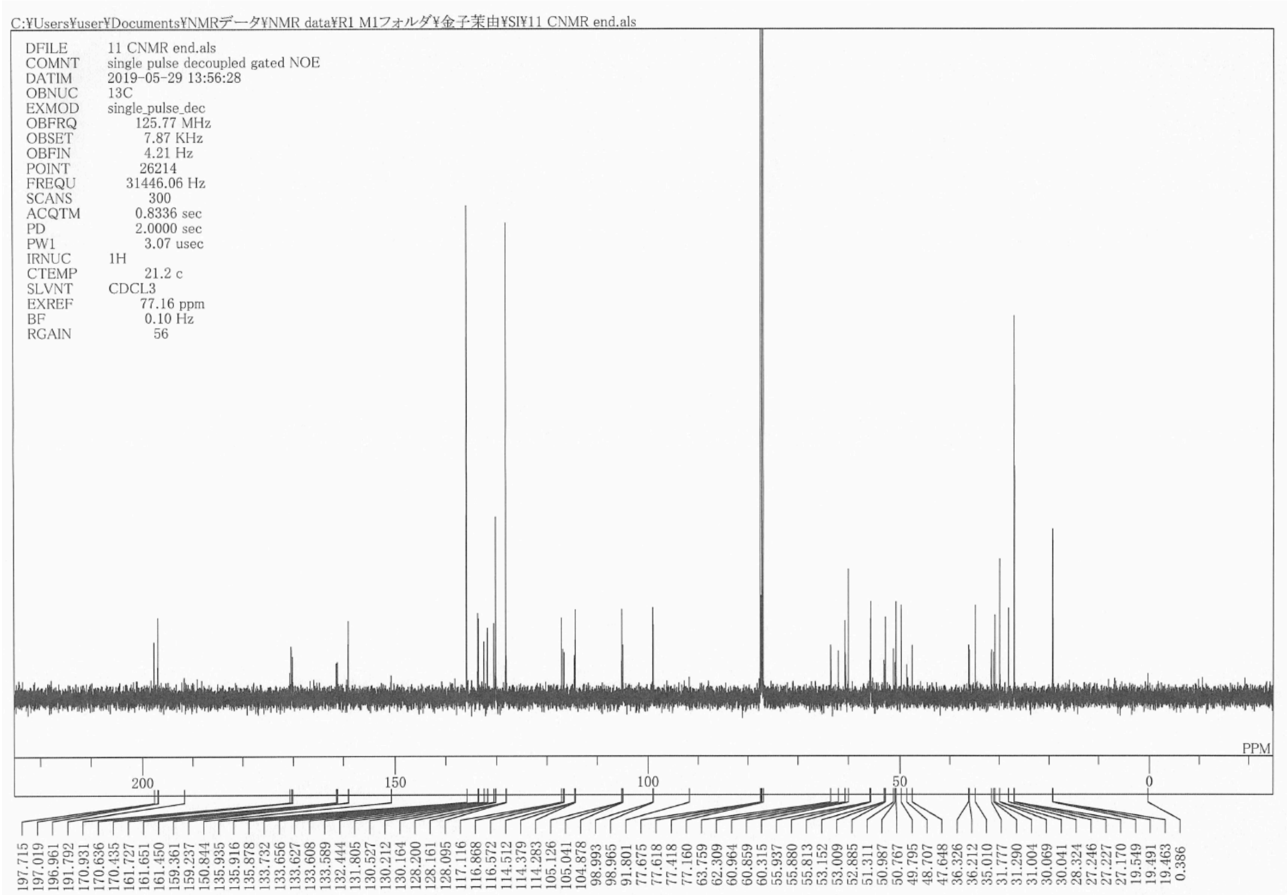
C:¥Users¥user¥Documents¥NMRデータ¥NMR data¥R1 M1フォルダ¥金子茉由¥SI¥11 CNMR end.als
DFILE 11 CNMR end.als
COMNT single pulse decoupled gated NOE
DATIM 2019-05-29 13:56:28
OBNUC 13C
EXMOD single_pulse_dec
OBFRQ 125.77 MHz
OBSET 7.87 KHz
OBFIN 4.21 Hz
POINT 26214
FREQU 31446.06 Hz
SCANS 300
ACQTM 0.8336 sec
PD 2.0000 sec
PW1 3.07 usec
IRNUC 1H
CTEMP 21.2 c
SLVNT CDCL3
EXREF 77.16 ppm
BF 0.10 Hz
RGAIN 56
PPM
200
150
100
50
0
197.715 197.019 196.961 191.792 170.931 170.636 170.435 161.727 161.651 161.450 159.361 159.237 150.844 135.935 135.916 135.878 133.732 133.656 133.627 133.608 133.589 132.444 131.805 130.527 130.212 130.164 128.200 128.161 128.095 117.116 116.868 116.572 114.512 114.379 114.283 105.126 105.041 104.878 98.993 98.965 91.801 77.675 77.618 77.418 77.160 63.759 62.309 60.964 60.859 60.315 55.937 55.880 55.813 53.152 53.009 52.885 51.311 50.987 50.767 49.795 48.707 47.648 36.326 36.212 35.010 31.777 31.290 31.004 30.069 30.041 28.324 27.246 27.227 27.170 19.549 19.491 19.463 0.386

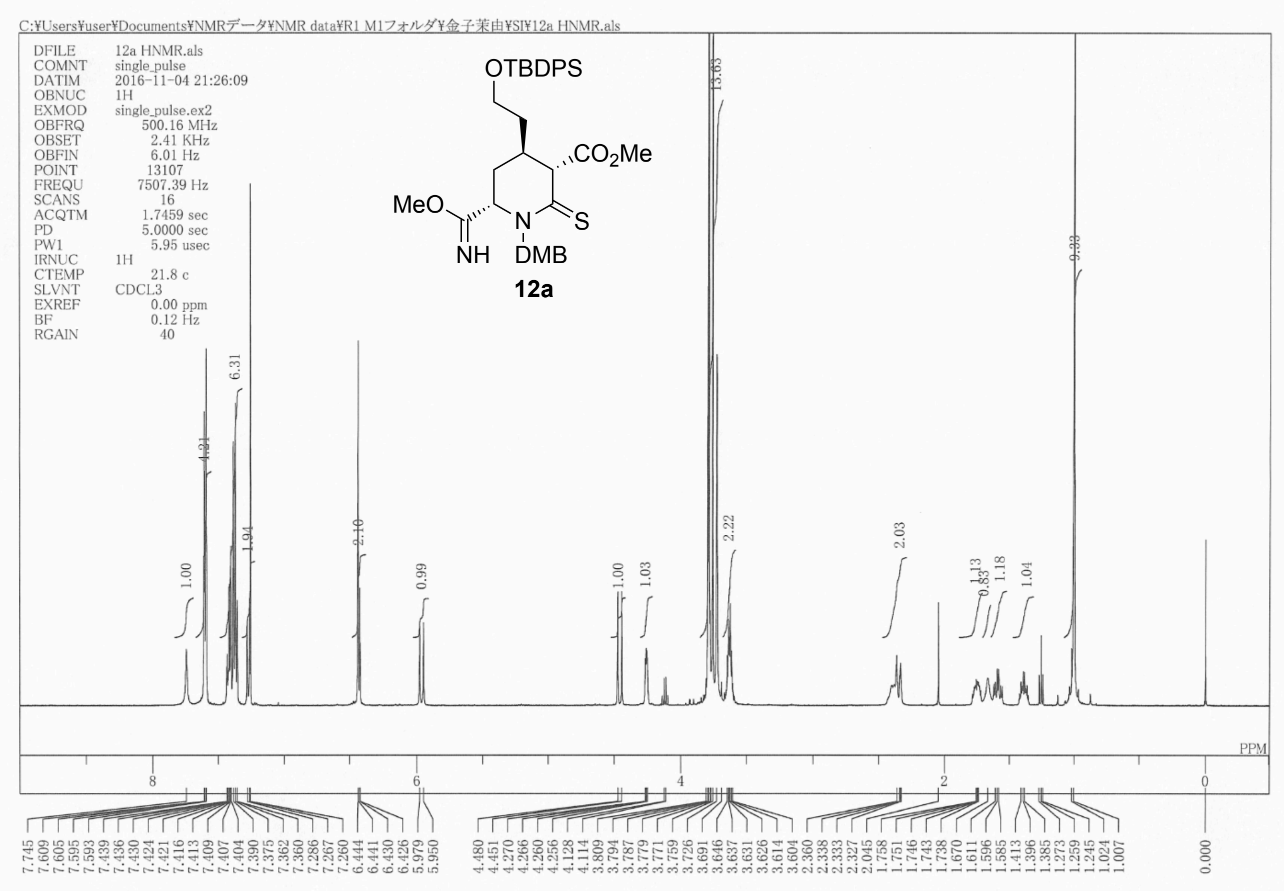
C:¥Users¥user¥Documents¥NMRデータ¥NMR data¥R1 M1フォルダ¥金子茉由¥SI¥12a HNMR.als
DFILE 12a HNMR.als
COMNT single_pulse
DATIM 2016-11-04 21:26:09
OBNUC 1H
EXMOD single_pulse.ex2
OBFRQ 500.16 MHz
OBSET 2.41 KHz
OBFIN 6.01 Hz
POINT 13107
FREQU 7507.39 Hz
SCANS 16
ACQTM 1.7459 sec
PD 5.0000 sec
PW1 5.95 usec
IRNUC 1H
CTEMP 21.8 c
SLVNT CDCL3
EXREF 0.00 ppm
BF 0.12 Hz
RGAIN 40
OTBDPS
CO2Me
MeO
N
S
NH
DMB
12a
PPM
7.745 7.609 7.605 7.595 7.593 7.439 7.436 7.430 7.424 7.421 7.416 7.413 7.409 7.407 7.404 7.390 7.375 7.362 7.360 7.286 7.267 7.260 6.444 6.441 6.430 6.426 5.979 5.950
4.480 4.451 4.270 4.266 4.260 4.256 4.128 4.114 3.809 3.794 3.787 3.779 3.771 3.759 3.726 3.691 3.646 3.637 3.631 3.626 3.614 3.604 2.360 2.338 2.333 2.327 2.045 1.758 1.751 1.746 1.743 1.738 1.670 1.611 1.596 1.585 1.413 1.396 1.385 1.273 1.259 1.245 1.024 1.007
0.000

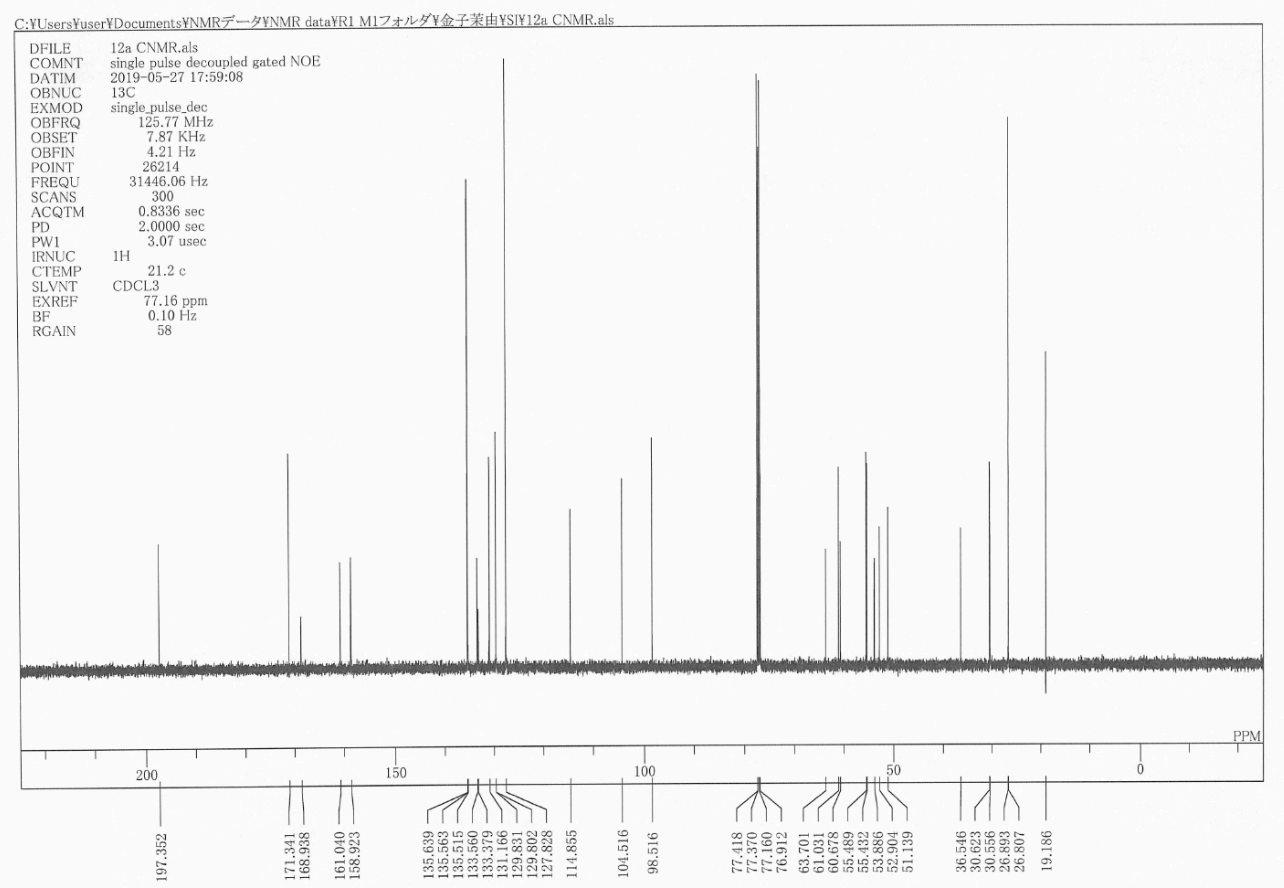
C:¥Users¥user¥Documents¥NMRデータ¥NMR data¥R1 M1フォルダ¥金子茉由¥SI¥12a CNMR.als
DFILE 12a CNMR.als
COMNT single pulse decoupled gated NOE
DATIM 2019-05-27 17:59:08
OBNUC 13C
EXMOD single_pulse_dec
OBFRQ 125.77 MHz
OBSET 7.87 KHz
OBFIN 4.21 Hz
POINT 26214
FREQU 31446.06 Hz
SCANS 300
ACQTM 0.8336 sec
PD 2.0000 sec
PW1 3.07 usec
IRNUC 1H
CTEMP 21.2 c
SLVNT CDCL3
EXREF 77.16 ppm
BF 0.10 Hz
RGAIN 58
PPM
197.352
171.341 168.938 161.040 158.923
135.639 135.563 135.515 133.560 133.379 131.166 129.831 129.802 127.828 114.855 104.516 98.516
77.418 77.370 77.160 76.912 63.701 61.031 60.678 55.489 55.432 53.886 52.904 51.139
36.546 30.623 30.556 26.893 26.807 19.186

=== Shimadzu LabSolutions Report ===

Sample Name	: TKP33 ee major 4		
Sample ID	: kaneko		
Data Filename	: TKP33 ee major 4.lcd		
Method Filename	: 10%iPrOH-Hex-flow0.5.lcm		
Batch Filename	:		
Vial#	: 1-1	Sample Type	: -¢'m
Injection Volume	: 2000 uL		
Date Acquired	: 2018/04/23 18:08:49 Acquired by		: System Administrator
Date Processed	: 2018/04/23 22:48:21 Processed by		: System Administrator

<Chromatogram>

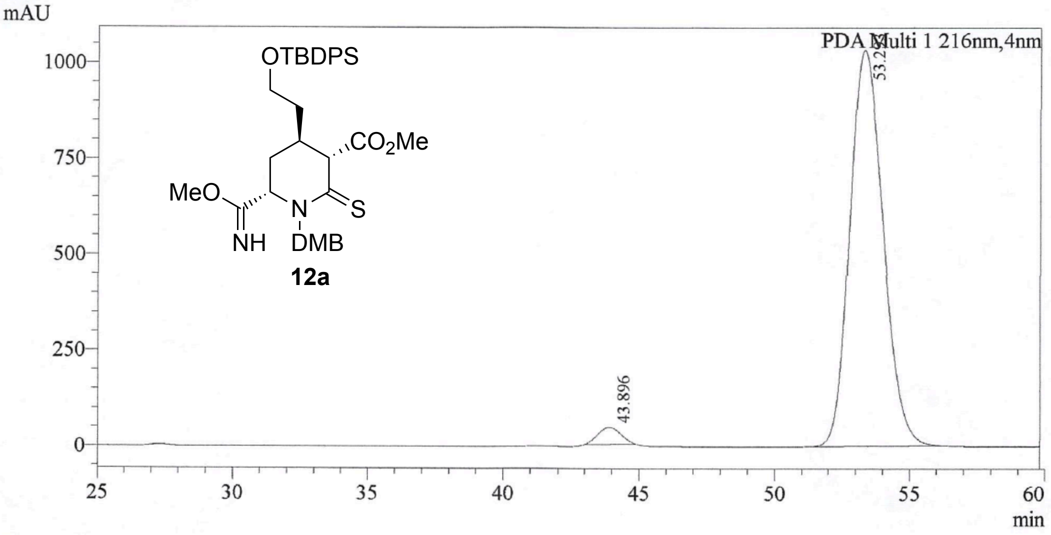

<Peak Table>

PDA Ch1 216nm

Peak#	Ret. Time	Area	Height	Conc.	Name
1	43.896	2611281	44203	2.866	
2	53.293	88506909	1032774	97.134	
‡Œv		91118190	1076977		

Racemic 12a

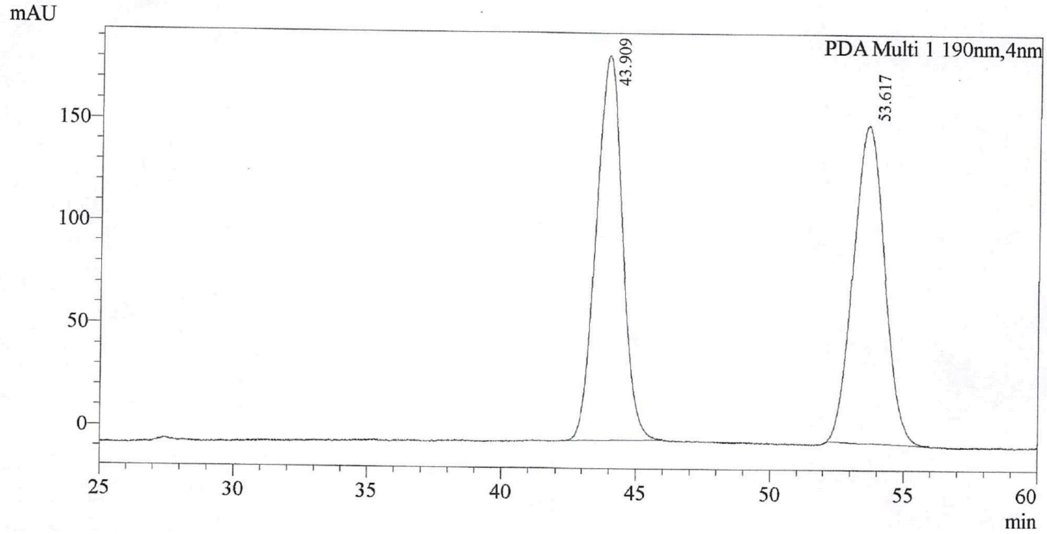

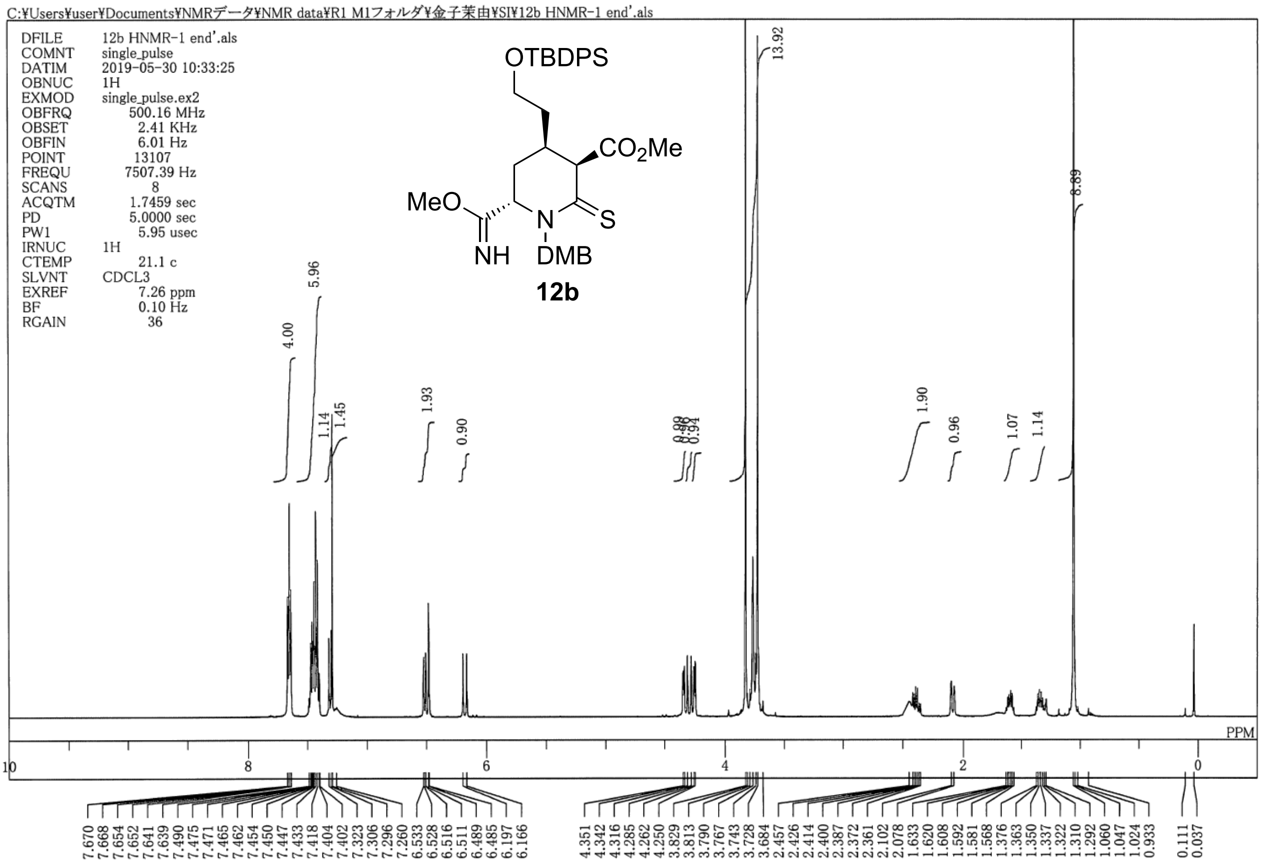
C:¥Users¥user¥Documents¥NMRデータ¥NMR data¥R1 M1フォルダ¥金子茉由¥SI¥12b HNMR-1 end'.als
DFILE 12b HNMR-1 end'.als
COMNT single_pulse
DATIM 2019-05-30 10:33:25
OBNUC 1H
EXMOD single_pulse.ex2
OBFRQ 500.16 MHz
OBSET 2.41 KHz
OBFIN 6.01 Hz
POINT 13107
FREQU 7507.39 Hz
SCANS 8
ACQTM 1.7459 sec
PD 5.0000 sec
PW1 5.95 usec
IRNUC 1H
CTEMP 21.1 c
SLVNT CDCL3
EXREF 7.26 ppm
BF 0.10 Hz
RGAIN 36
OTBDPS
CO2Me
MeO
N
S
NH
DMB
12b
4.00
5.96
1.14
1.45
1.93
0.90
13.92
1.90
0.96
1.07
1.14
8.89
PPM
10
8
6
4
2
0
7.670
7.668
7.654
7.652
7.641
7.639
7.490
7.475
7.471
7.465
7.462
7.454
7.450
7.447
7.433
7.418
7.404
7.402
7.323
7.306
7.296
7.260
6.533
6.528
6.516
6.511
6.489
6.485
6.197
6.166
4.351
4.342
4.316
4.285
4.262
4.250
3.829
3.813
3.790
3.767
3.743
3.728
3.684
2.457
2.426
2.414
2.400
2.387
2.372
2.361
2.102
2.078
1.633
1.620
1.608
1.592
1.581
1.568
1.376
1.363
1.350
1.337
1.322
1.310
1.292
1.060
1.047
1.024
0.933
0.111
0.037

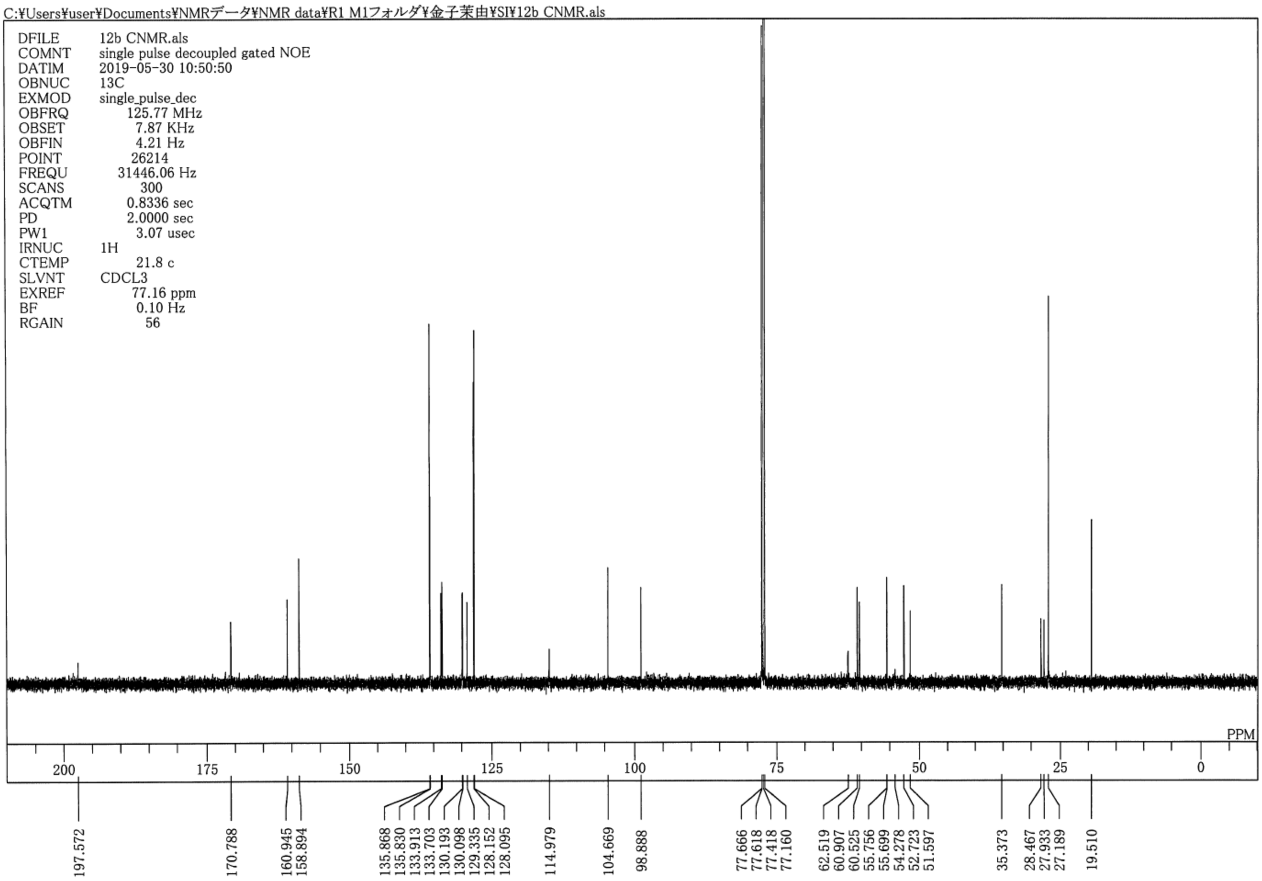
C:¥Users¥user¥Documents¥NMRデータ¥NMR data¥R1 M1フォルダ¥金子茉由¥SI¥12b CNMR.als
DFILE 12b CNMR.als
COMNT single pulse decoupled gated NOE
DATIM 2019-05-30 10:50:50
OBNUC 13C
EXMOD single_pulse_dec
OBFRQ 125.77 MHz
OBSET 7.87 KHz
OBFIN 4.21 Hz
POINT 26214
FREQU 31446.06 Hz
SCANS 300
ACQTM 0.8336 sec
PD 2.0000 sec
PW1 3.07 usec
IRNUC 1H
CTEMP 21.8 c
SLVNT CDCL3
EXREF 77.16 ppm
BF 0.10 Hz
RGAIN 56
PPM
200
175
150
125
100
75
50
25
0
197.572
170.788
160.945
158.894
135.868
135.830
133.913
133.703
130.193
130.098
129.335
128.152
128.095
114.979
104.669
98.888
77.666
77.618
77.418
77.160
62.519
60.907
60.525
55.756
55.699
54.278
52.723
51.597
35.373
28.467
27.933
27.189
19.510

=== Shimadzu LabSolutions Report ===

Sample Name	: compound 12b chiral IC		
Sample ID	: kaneko		
Data Filename	: compound 12b chiral IC.lcd		
Method Filename	: 20%iPrOH-Hex-flow0.75.lcm		
Batch Filename	:		
Vial#	: 1-1	Sample Type	: −¢'m
Injection Volume	: 20 uL		
Date Acquired	: 2019/07/12 16:34:38	Acquired by	: System Administrator
Date Processed	: 2019/07/12 17:17:06	Processed by	: System Administrator

<Chromatogram>

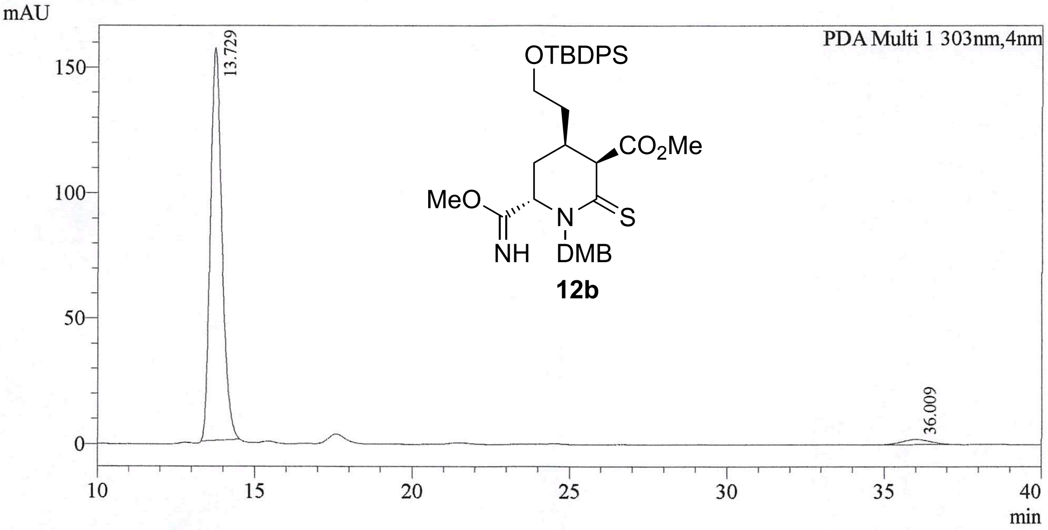

<Peak Table>

PDA Ch1 303nm

Peak#	Ret. Time	Area	Height	Conc.	Name
1	13.729	3983503	156496	96.861	
2	36.009	129109	2000	3.139	
‡Œv		4112611	158497		

Racemic 12b

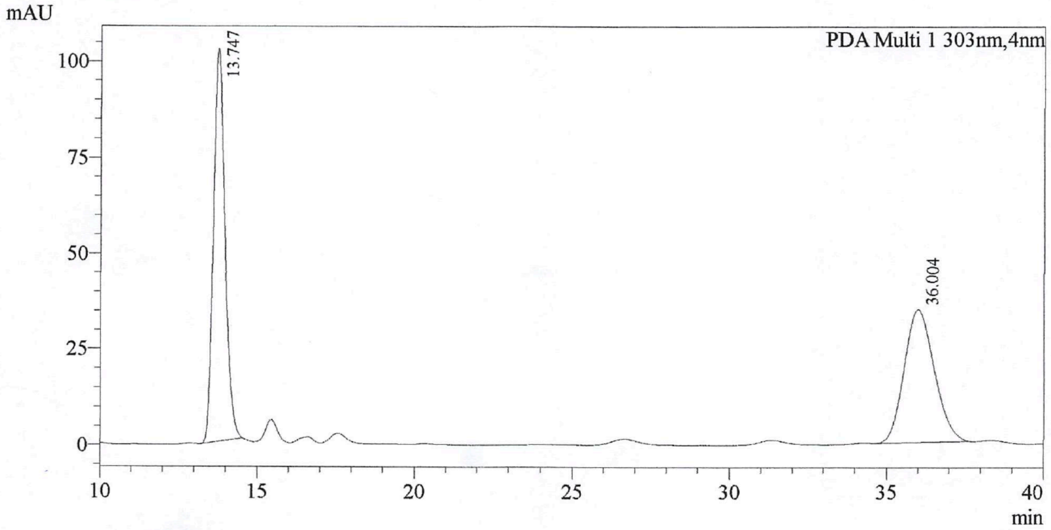

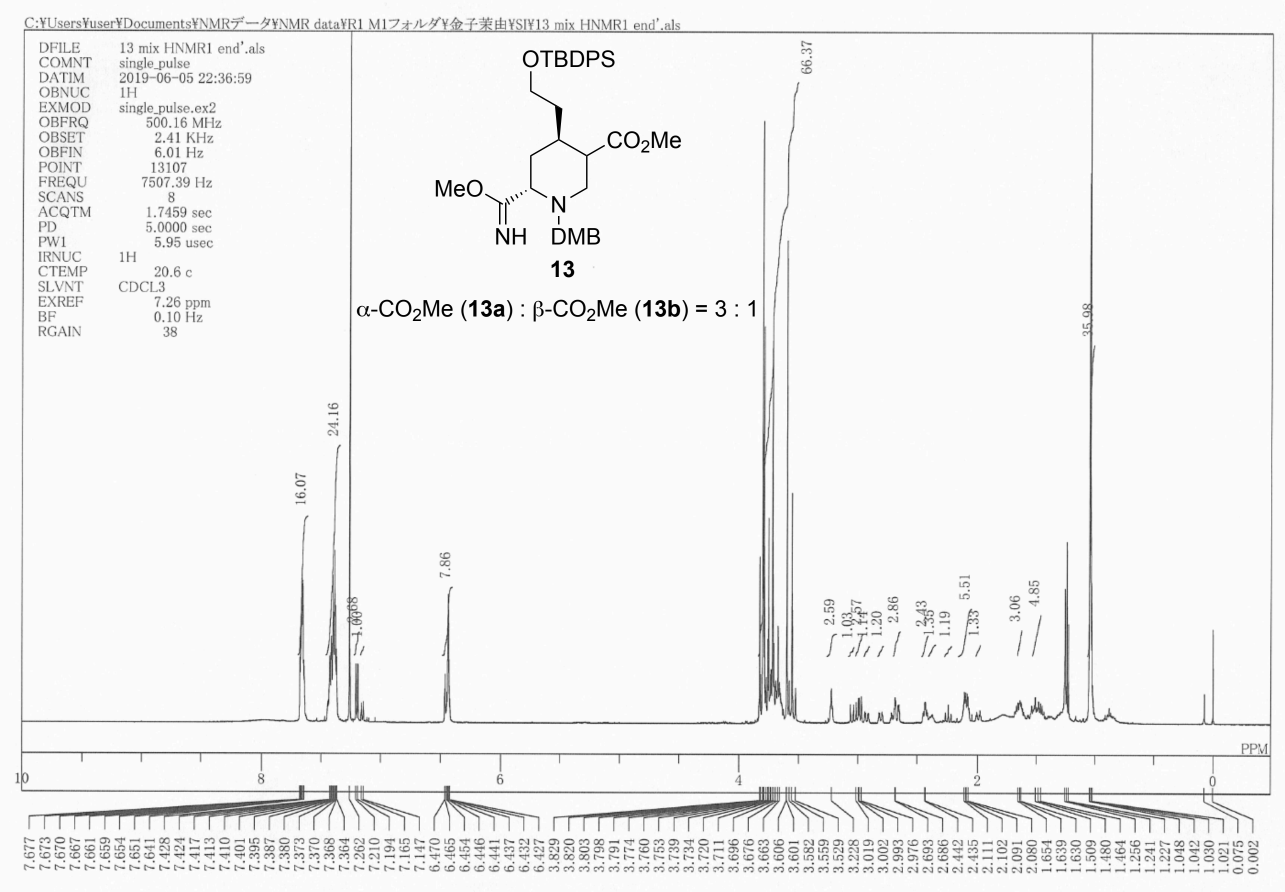
C:¥Users¥user¥Documents¥NMRデータ¥NMR data¥R1 M1フォルダ¥金子茉由¥SI¥13 mix HNMR1 end'.als
DFILE 13 mix HNMR1 end'.als
COMNT single_pulse
DATIM 2019-06-05 22:36:59
OBNUC 1H
EXMOD single_pulse.ex2
OBFRQ 500.16 MHz
OBSET 2.41 KHz
OBFIN 6.01 Hz
POINT 13107
FREQU 7507.39 Hz
SCANS 8
ACQTM 1.7459 sec
PD 5.0000 sec
PW1 5.95 usec
IRNUC 1H
CTEMP 20.6 c
SLVNT CDCL3
EXREF 7.26 ppm
BF 0.10 Hz
RGAIN 38
OTBDPS
CO_2Me
MeO
NH
N
DMB
13
α-CO_2Me (13a) : β-CO_2Me (13b) = 3 : 1
66.37
35.98
24.16
16.07
7.86
2.59
2.86
2.43
5.51
3.06
4.85
1.19
1.33
1.20
PPM
10
8
6
4
2
0
7.677
7.673
7.670
7.667
7.661
7.659
7.654
7.651
7.641
7.428
7.424
7.417
7.413
7.410
7.401
7.395
7.387
7.380
7.373
7.370
7.368
7.364
7.262
7.210
7.194
7.165
7.147
6.470
6.465
6.454
6.446
6.441
6.437
6.432
6.427
3.829
3.820
3.803
3.798
3.791
3.774
3.760
3.753
3.739
3.734
3.720
3.711
3.696
3.676
3.663
3.606
3.601
3.582
3.559
3.529
3.228
3.019
3.002
2.993
2.976
2.693
2.686
2.442
2.435
2.111
2.102
2.091
2.080
1.654
1.639
1.630
1.509
1.480
1.464
1.256
1.241
1.227
1.048
1.042
1.030
1.021
0.075
0.002

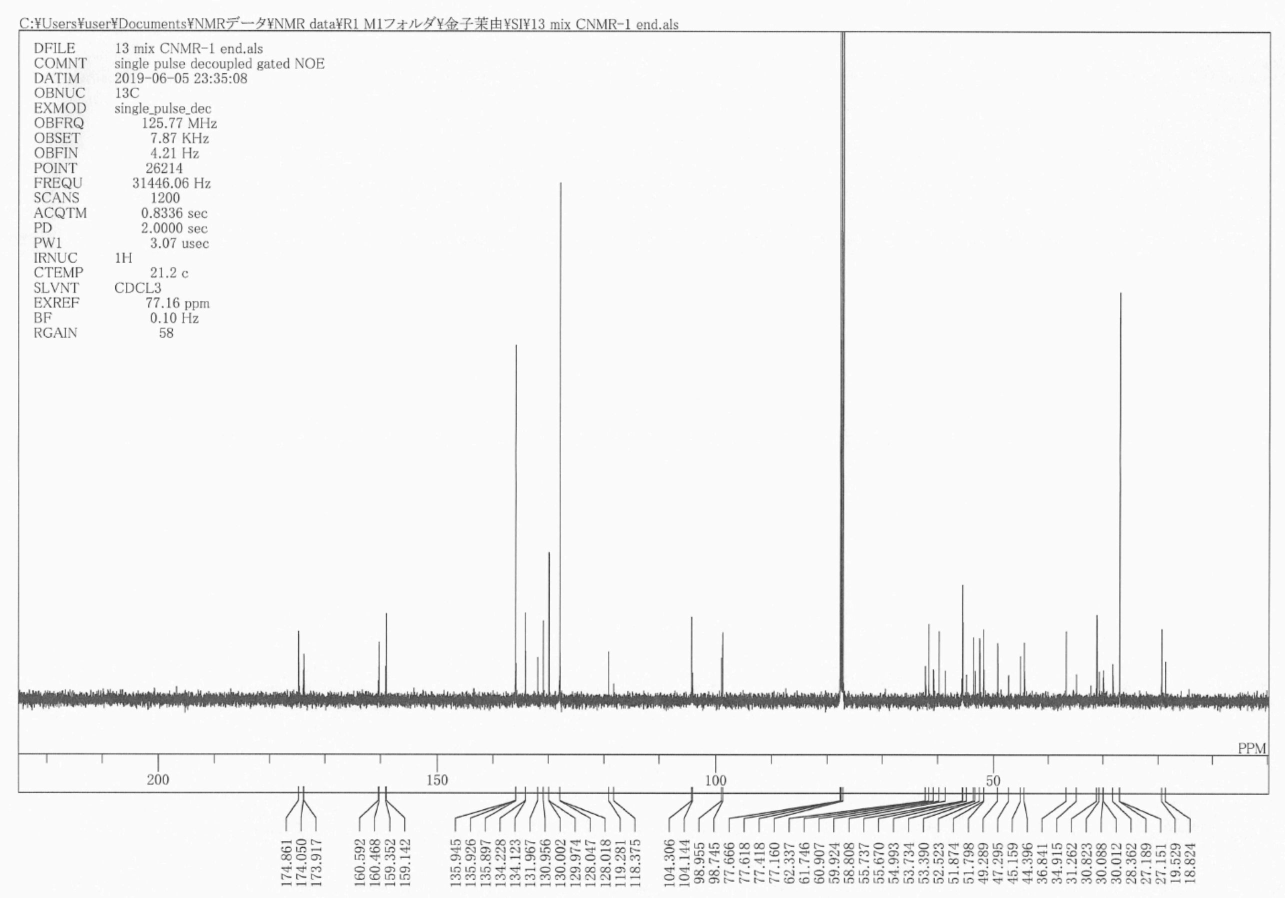
C:¥Users¥user¥Documents¥NMRデータ¥NMR data¥R1 M1フォルダ¥金子茉由¥SI¥13 mix CNMR-1 end.als
DFILE 13 mix CNMR-1 end.als
COMNT single pulse decoupled gated NOE
DATIM 2019-06-05 23:35:08
OBNUC 13C
EXMOD single_pulse_dec
OBFRQ 125.77 MHz
OBSET 7.87 KHz
OBFIN 4.21 Hz
POINT 26214
FREQU 31446.06 Hz
SCANS 1200
ACQTM 0.8336 sec
PD 2.0000 sec
PW1 3.07 usec
IRNUC 1H
CTEMP 21.2 c
SLVNT CDCL3
EXREF 77.16 ppm
BF 0.10 Hz
RGAIN 58
PPM
200
150
100
50
174.861
174.050
173.917
160.592
160.468
159.352
159.142
135.945
135.926
135.897
134.228
134.123
131.967
130.956
130.002
129.974
129.047
128.018
119.281
118.375
104.306
104.144
98.955
98.745
77.666
77.618
77.418
77.160
62.337
61.746
60.907
59.924
58.808
55.737
55.670
54.993
53.734
53.390
52.523
51.874
51.798
49.289
47.295
45.159
44.396
36.841
34.915
31.262
30.823
30.088
30.012
28.362
27.189
27.151
19.529
18.824

C:¥Users¥user¥Documents¥NMRデータ¥NMR data¥R1 M1フォルダ¥金子茉由¥SI¥13a HNMR.als

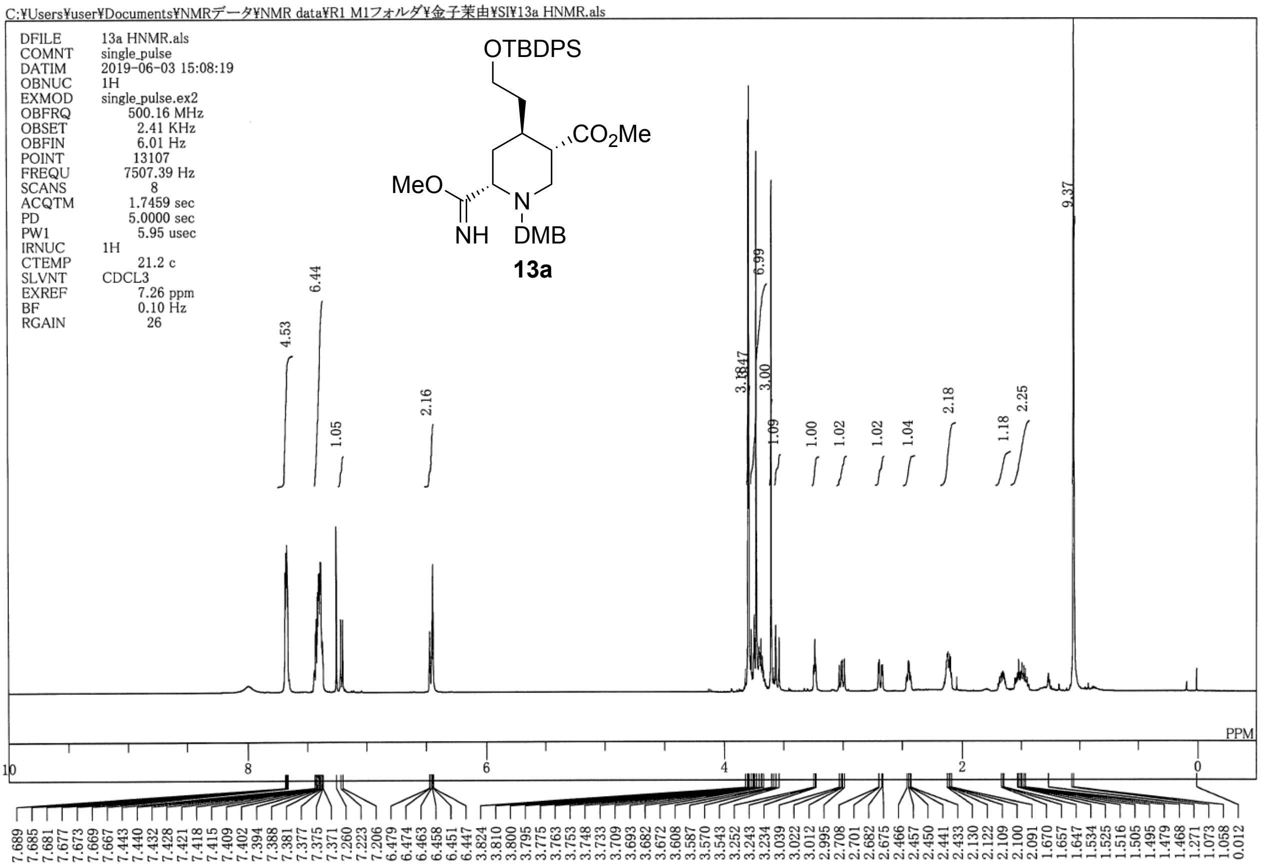

C:¥Users¥user¥Documents¥NMRデータ¥NMR data¥R1 M1フォルダ¥金子茉由¥SI¥13a CNMR.als

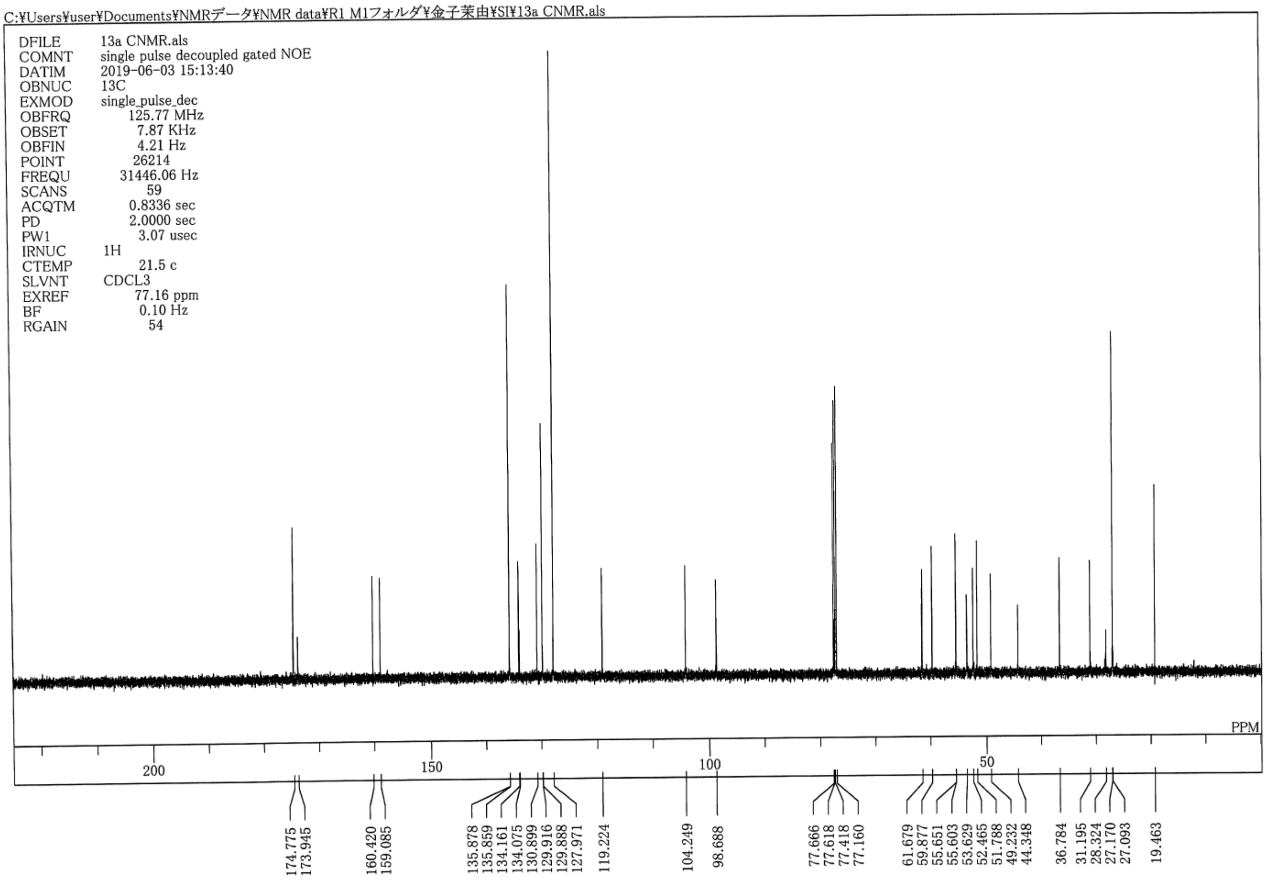

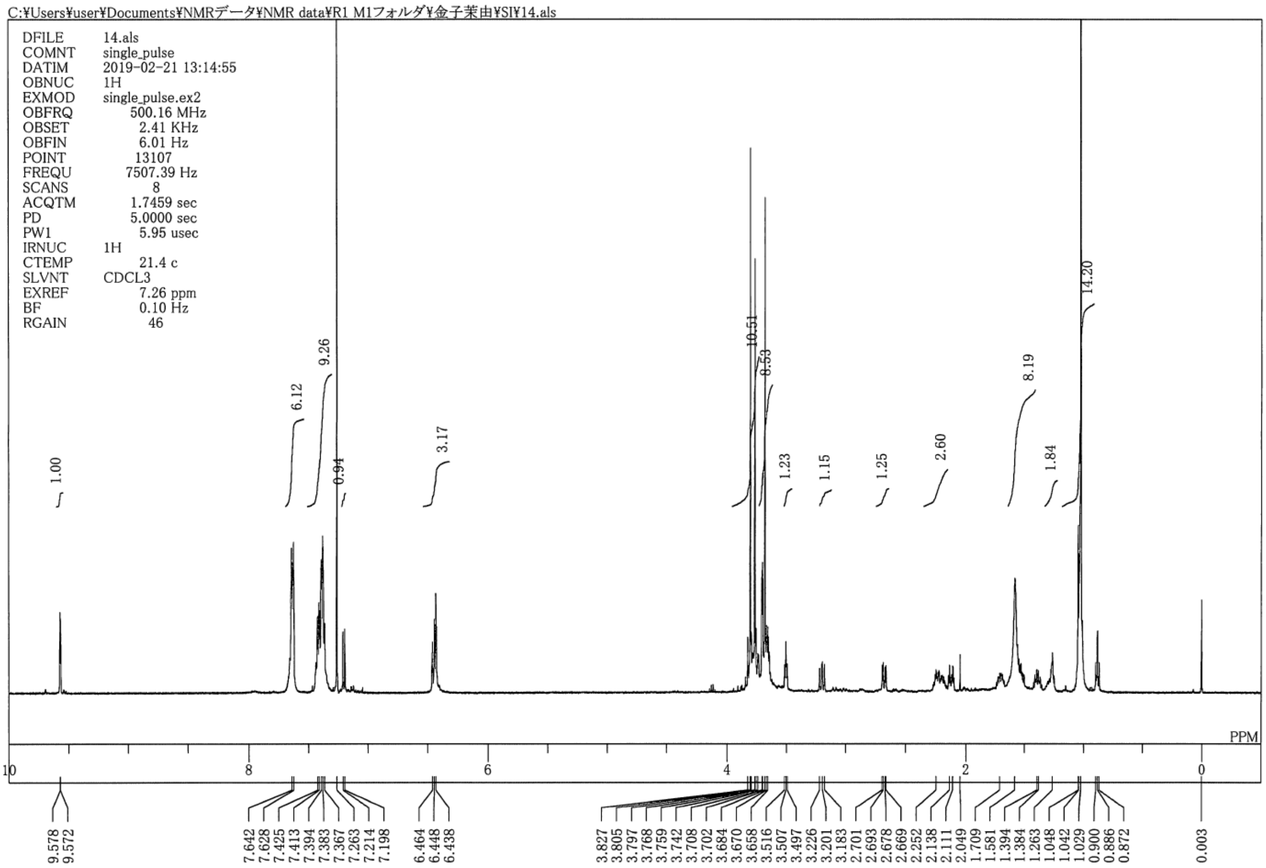

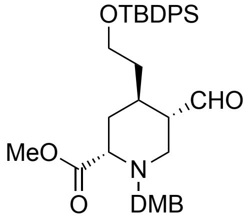

14

Single isomer determined by crude ^{1}H-NMR

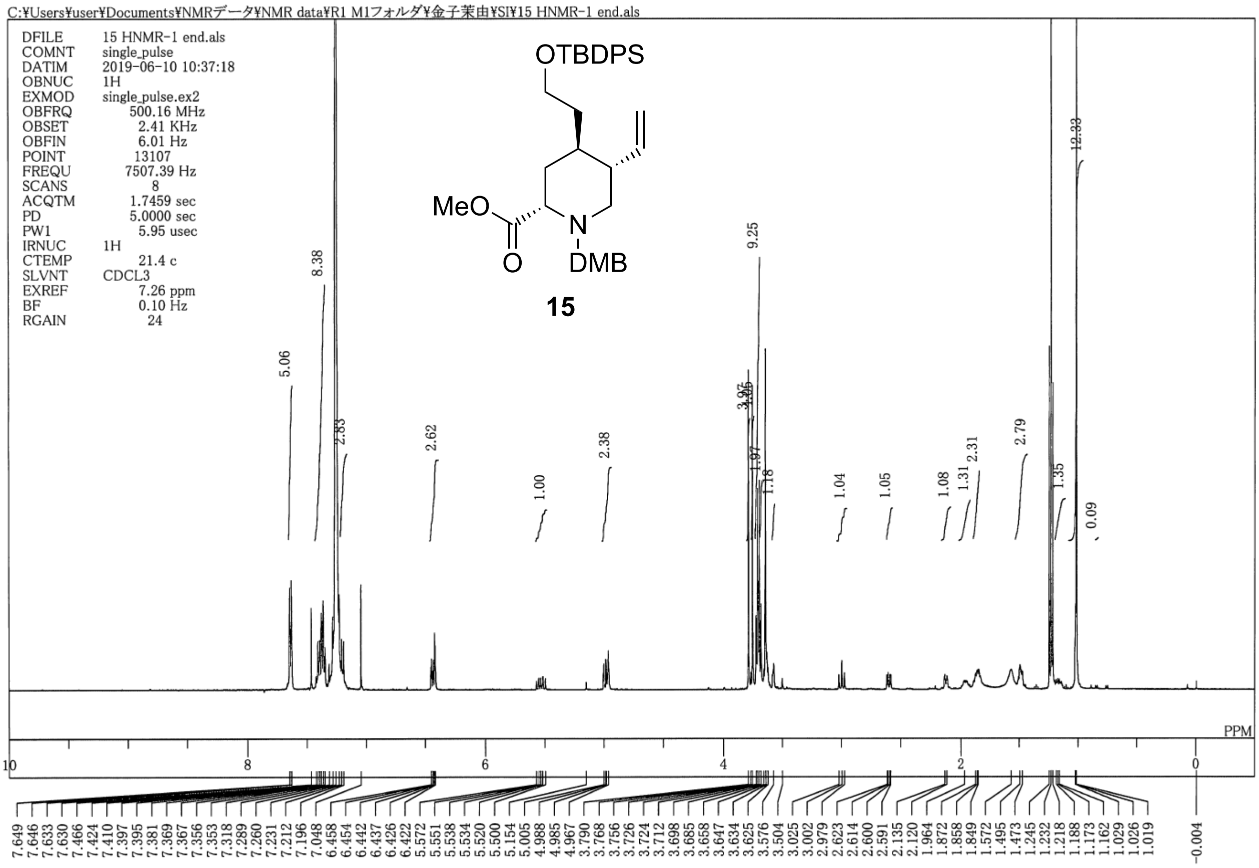
C:¥Users¥user¥Documents¥NMRデータ¥NMR data¥R1 M1フォルダ¥金子茉由¥SI¥15 HNMR-1 end.als
DFILE 15 HNMR-1 end.als
COMNT single_pulse
DATIM 2019-06-10 10:37:18
OBNUC 1H
EXMOD single_pulse.ex2
OBFRQ 500.16 MHz
OBSET 2.41 KHz
OBFIN 6.01 Hz
POINT 13107
FREQU 7507.39 Hz
SCANS 8
ACQTM 1.7459 sec
PD 5.0000 sec
PW1 5.95 usec
IRNUC 1H
CTEMP 21.4 c
SLVNT CDCL3
EXREF 7.26 ppm
BF 0.10 Hz
RGAIN 24
OTBDPS
MeO
N
O
DMB
15
PPM
7.649 7.646 7.633 7.630 7.466 7.424 7.410 7.397 7.395 7.381 7.369 7.367 7.356 7.353 7.318 7.289 7.260 7.231 7.212 7.196 7.048 6.458 6.454 6.442 6.437 6.426 6.422 5.572 5.551 5.538 5.534 5.520 5.500 5.154 5.005 4.988 4.985 4.967 3.790 3.768 3.756 3.726 3.724 3.712 3.698 3.685 3.658 3.647 3.634 3.625 3.576 3.504 3.025 3.002 2.979 2.623 2.614 2.600 2.591 2.135 2.120 1.964 1.872 1.858 1.849 1.572 1.495 1.473 1.245 1.232 1.218 1.188 1.173 1.162 1.029 1.026 1.019 -0.004

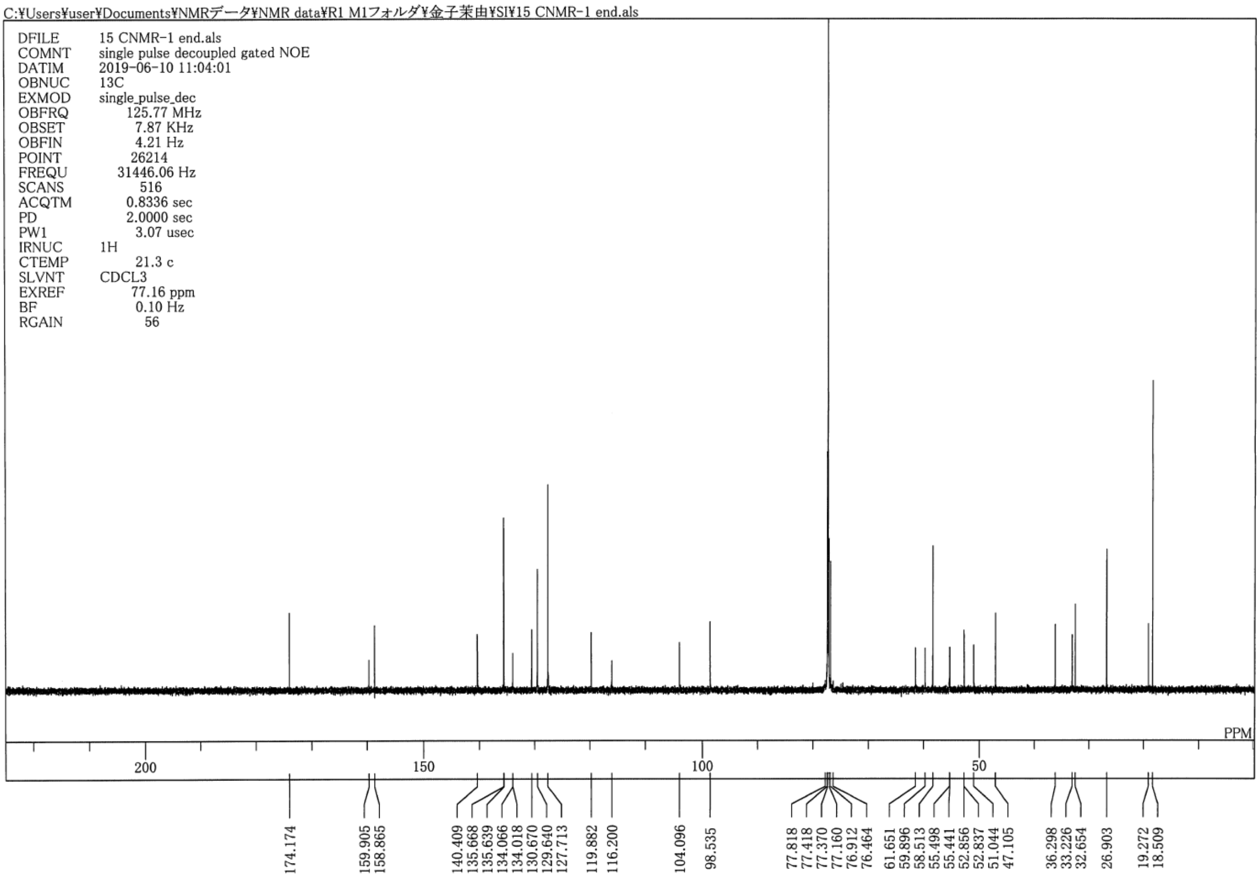
C:¥Users¥user¥Documents¥NMRデータ¥NMR data¥R1 M1フォルダ¥金子茉由¥SI¥15 CNMR-1 end.als
DFILE 15 CNMR-1 end.als
COMNT single pulse decoupled gated NOE
DATIM 2019-06-10 11:04:01
OBNUC 13C
EXMOD single_pulse_dec
OBFRQ 125.77 MHz
OBSET 7.87 KHz
OBFIN 4.21 Hz
POINT 26214
FREQU 31446.06 Hz
SCANS 516
ACQTM 0.8336 sec
PD 2.0000 sec
PW1 3.07 usec
IRNUC 1H
CTEMP 21.3 c
SLVNT CDCL3
EXREF 77.16 ppm
BF 0.10 Hz
RGAIN 56
PPM
174.174 159.905 158.865 140.409 135.668 135.639 134.066 134.018 130.670 129.640 127.713 119.882 116.200 104.096 98.535 77.818 77.418 77.370 77.160 76.912 76.464 61.651 59.896 58.513 55.498 55.441 52.856 52.837 51.044 47.105 36.298 33.226 32.654 26.903 19.272 18.509

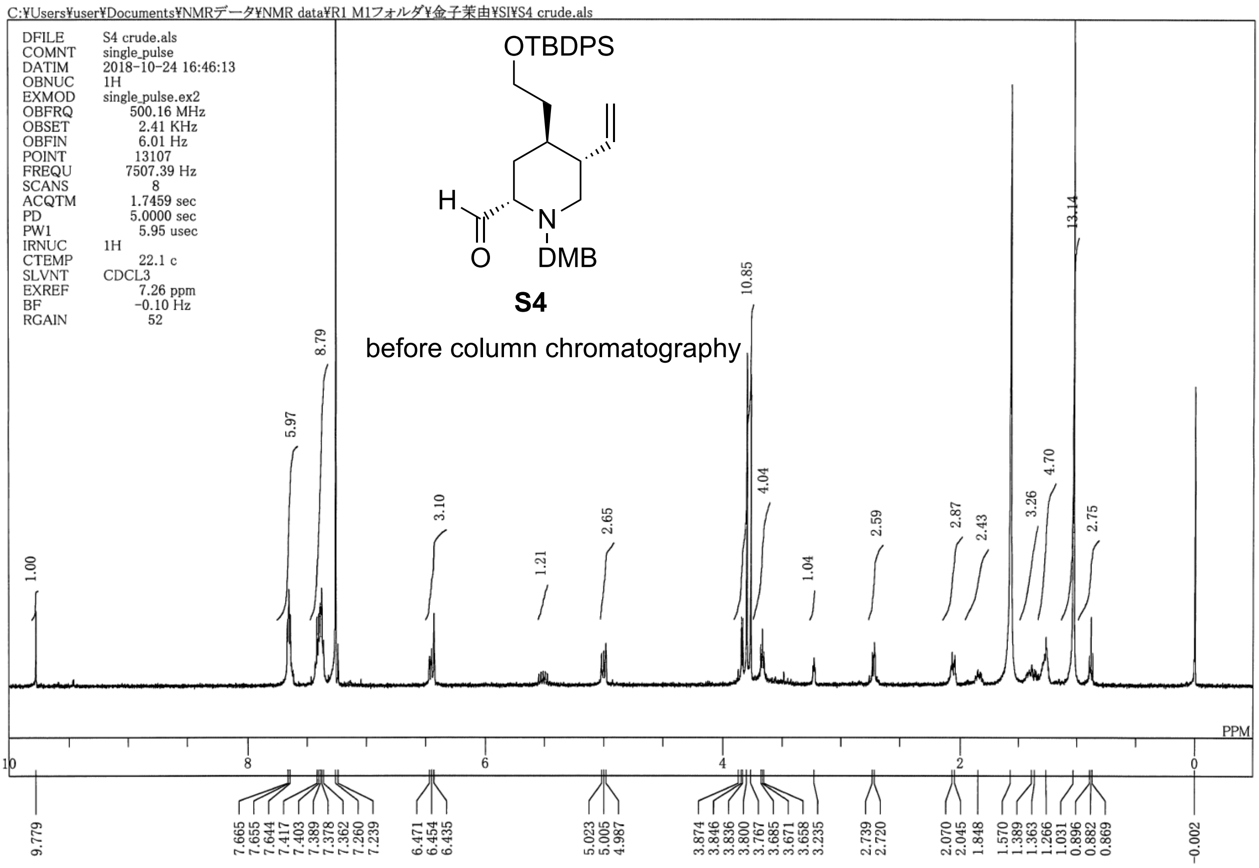
C:¥Users¥user¥Documents¥NMRデータ¥NMR data¥R1 M1フォルダ¥金子茉由¥SI¥S4 crude.als
DFILE S4 crude.als
COMNT single_pulse
DATIM 2018-10-24 16:46:13
OBNUC 1H
EXMOD single_pulse.ex2
OBFRQ 500.16 MHz
OBSET 2.41 KHz
OBFIN 6.01 Hz
POINT 13107
FREQU 7507.39 Hz
SCANS 8
ACQTM 1.7459 sec
PD 5.0000 sec
PW1 5.95 usec
IRNUC 1H
CTEMP 22.1 c
SLVNT CDCL3
EXREF 7.26 ppm
BF -0.10 Hz
RGAIN 52
OTBDPS
H
N
O
DMB
S4
before column chromatography
PPM

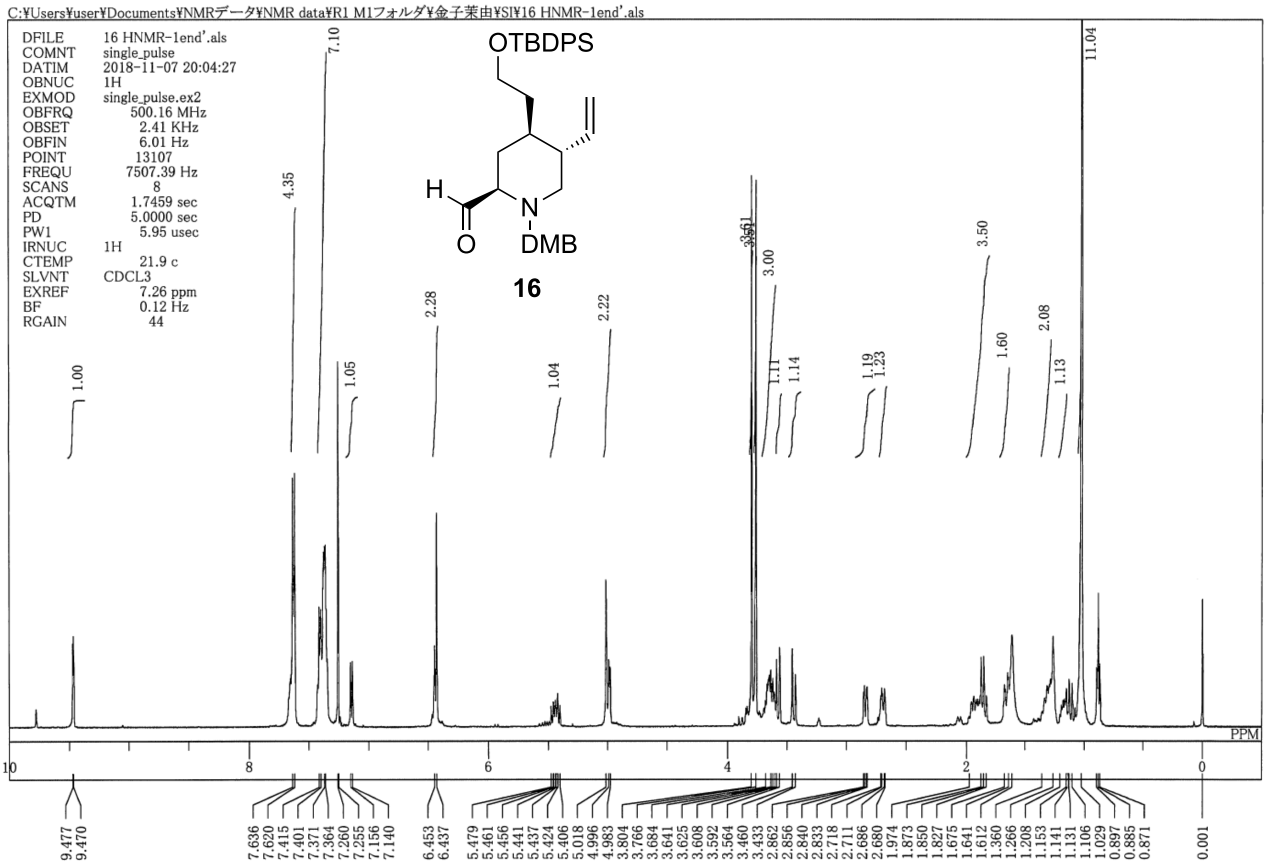
C:¥Users¥user¥Documents¥NMRデータ¥NMR data¥R1 M1フォルダ¥金子茉由¥SI¥16 HNMR-1end'.als
DFILE 16 HNMR-1end'.als
COMNT single_pulse
DATIM 2018-11-07 20:04:27
OBNUC 1H
EXMOD single_pulse.ex2
OBFRQ 500.16 MHz
OBSET 2.41 KHz
OBFIN 6.01 Hz
POINT 13107
FREQU 7507.39 Hz
SCANS 8
ACQTM 1.7459 sec
PD 5.0000 sec
PW1 5.95 usec
IRNUC 1H
CTEMP 21.9 c
SLVNT CDCL3
EXREF 7.26 ppm
BF 0.12 Hz
RGAIN 44
OTBDPS
H
O
N
DMB
16
PPM

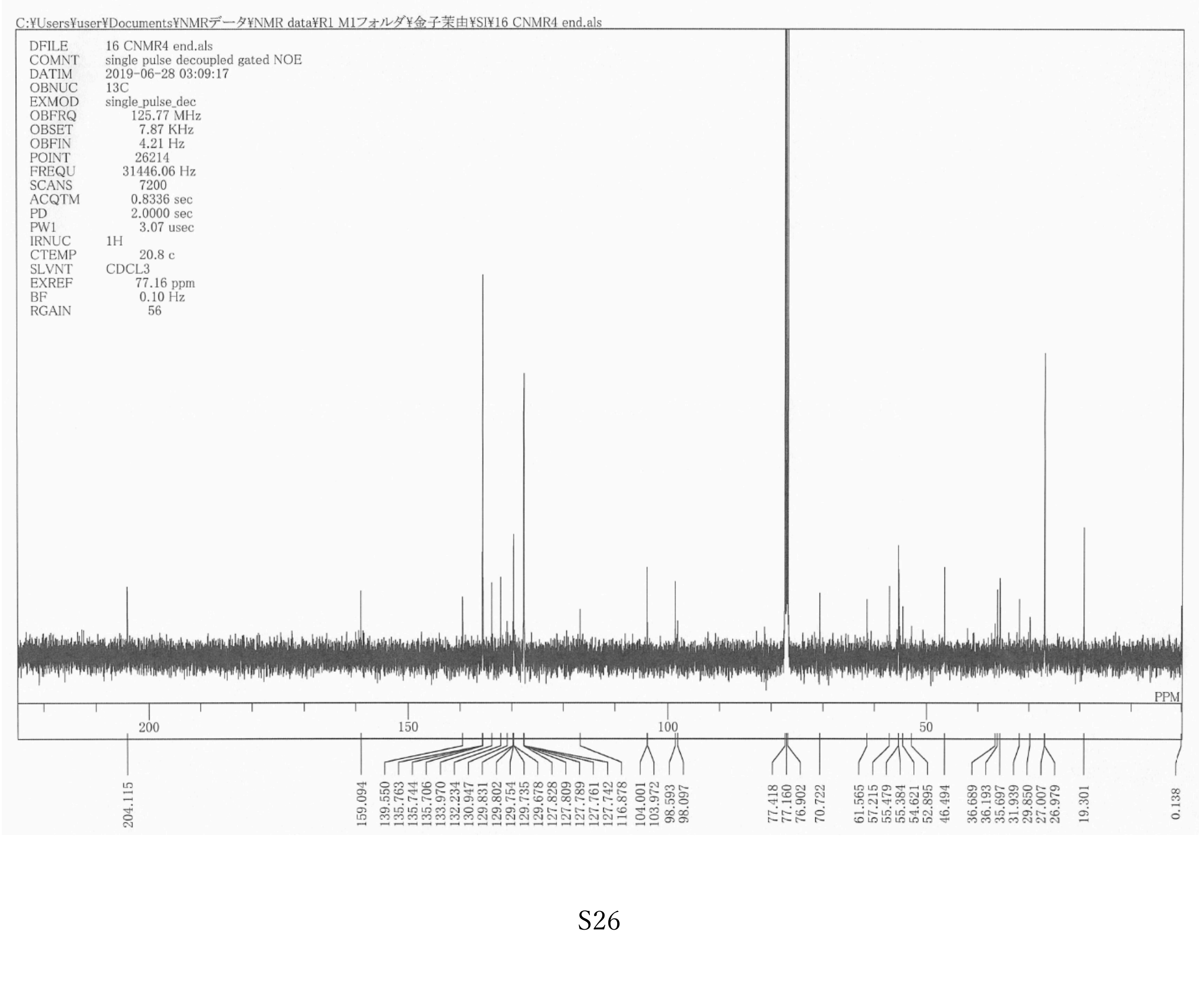
C:¥Users¥user¥Documents¥NMRデータ¥NMR data¥R1 M1フォルダ¥金子茉由¥SI¥16 CNMR4 end.als
DFILE 16 CNMR4 end.als
COMNT single pulse decoupled gated NOE
DATIM 2019-06-28 03:09:17
OBNUC 13C
EXMOD single_pulse_dec
OBFRQ 125.77 MHz
OBSET 7.87 KHz
OBFIN 4.21 Hz
POINT 26214
FREQU 31446.06 Hz
SCANS 7200
ACQTM 0.8336 sec
PD 2.0000 sec
PW1 3.07 usec
IRNUC 1H
CTEMP 20.8 c
SLVNT CDCL3
EXREF 77.16 ppm
BF 0.10 Hz
RGAIN 56
PPM

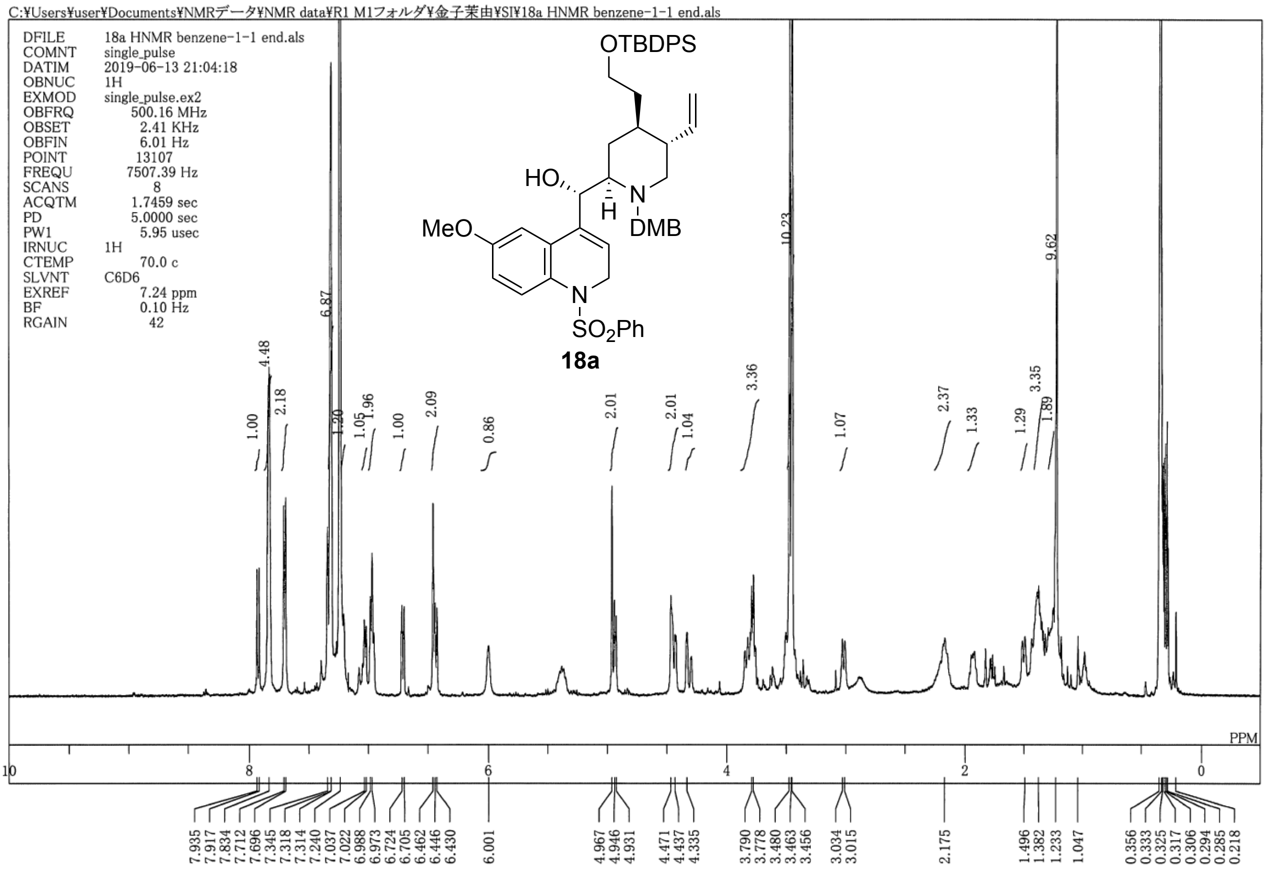
C:¥Users¥user¥Documents¥NMRデータ¥NMR data¥R1 M1フォルダ¥金子茉由¥SI¥18a HNMR benzene-1-1 end.als
DFILE 18a HNMR benzene-1-1 end.als
COMNT single_pulse
DATIM 2019-06-13 21:04:18
OBNUC 1H
EXMOD single_pulse.ex2
OBFRQ 500.16 MHz
OBSET 2.41 KHz
OBFIN 6.01 Hz
POINT 13107
FREQU 7507.39 Hz
SCANS 8
ACQTM 1.7459 sec
PD 5.0000 sec
PW1 5.95 usec
IRNUC 1H
CTEMP 70.0 c
SLVNT C6D6
EXREF 7.24 ppm
BF 0.10 Hz
RGAIN 42
OTBDPS
HO
H
N
DMB
MeO
N
SO2Ph
18a
1.00 4.48 2.18 6.87 1.20 1.05 1.96 1.00 2.09 0.86 2.01 2.01 1.04 3.36 10.23 1.07 2.37 1.33 1.29 3.35 9.62 1.89
PPM
10 8 6 4 2 0
7.935 7.917 7.834 7.712 7.696 7.345 7.318 7.314 7.240 7.037 7.022 6.988 6.973 6.724 6.705 6.462 6.446 6.430 6.001 4.967 4.946 4.931 4.471 4.437 4.335 3.790 3.778 3.480 3.463 3.456 3.034 3.015 2.175 1.496 1.382 1.233 1.047 0.356 0.333 0.325 0.317 0.306 0.294 0.285 0.218

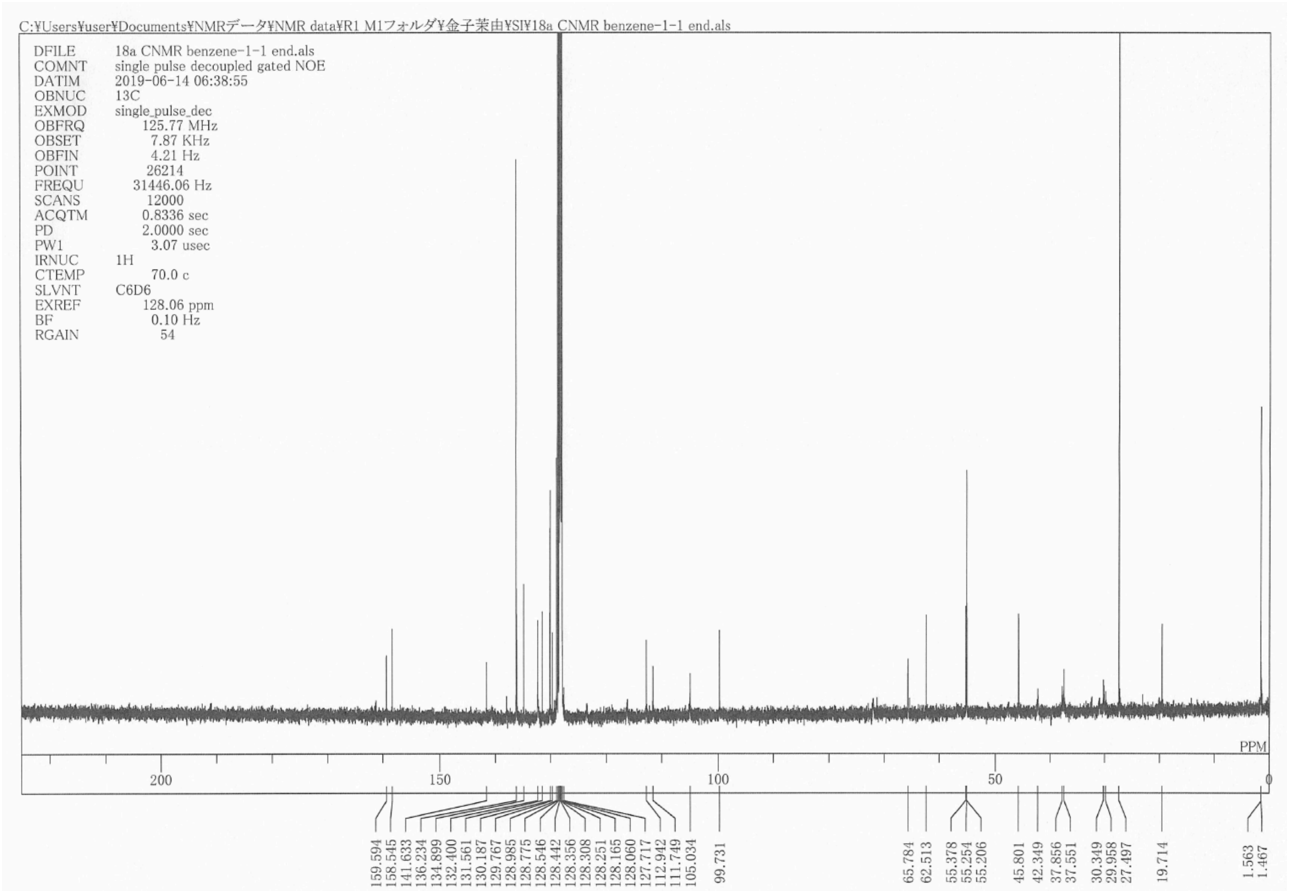
C:¥Users¥user¥Documents¥NMRデータ¥NMR data¥R1 M1フォルダ¥金子茉由¥SI¥18a CNMR benzene-1-1 end.als
DFILE 18a CNMR benzene-1-1 end.als
COMNT single pulse decoupled gated NOE
DATIM 2019-06-14 06:38:55
OBNUC 13C
EXMOD single_pulse_dec
OBFRQ 125.77 MHz
OBSET 7.87 KHz
OBFIN 4.21 Hz
POINT 26214
FREQU 31446.06 Hz
SCANS 12000
ACQTM 0.8336 sec
PD 2.0000 sec
PW1 3.07 usec
IRNUC 1H
CTEMP 70.0 c
SLVNT C6D6
EXREF 128.06 ppm
BF 0.10 Hz
RGAIN 54
PPM
200 150 100 50 0
159.594 158.545 141.633 136.234 134.899 132.400 131.561 130.187 129.767 128.985 128.775 128.546 128.442 128.356 128.308 128.251 128.165 128.060 127.717 112.942 111.749 105.034 99.731 65.784 62.513 55.378 55.254 55.206 45.801 42.349 37.856 37.551 30.349 29.958 27.497 19.714 1.563 1.467

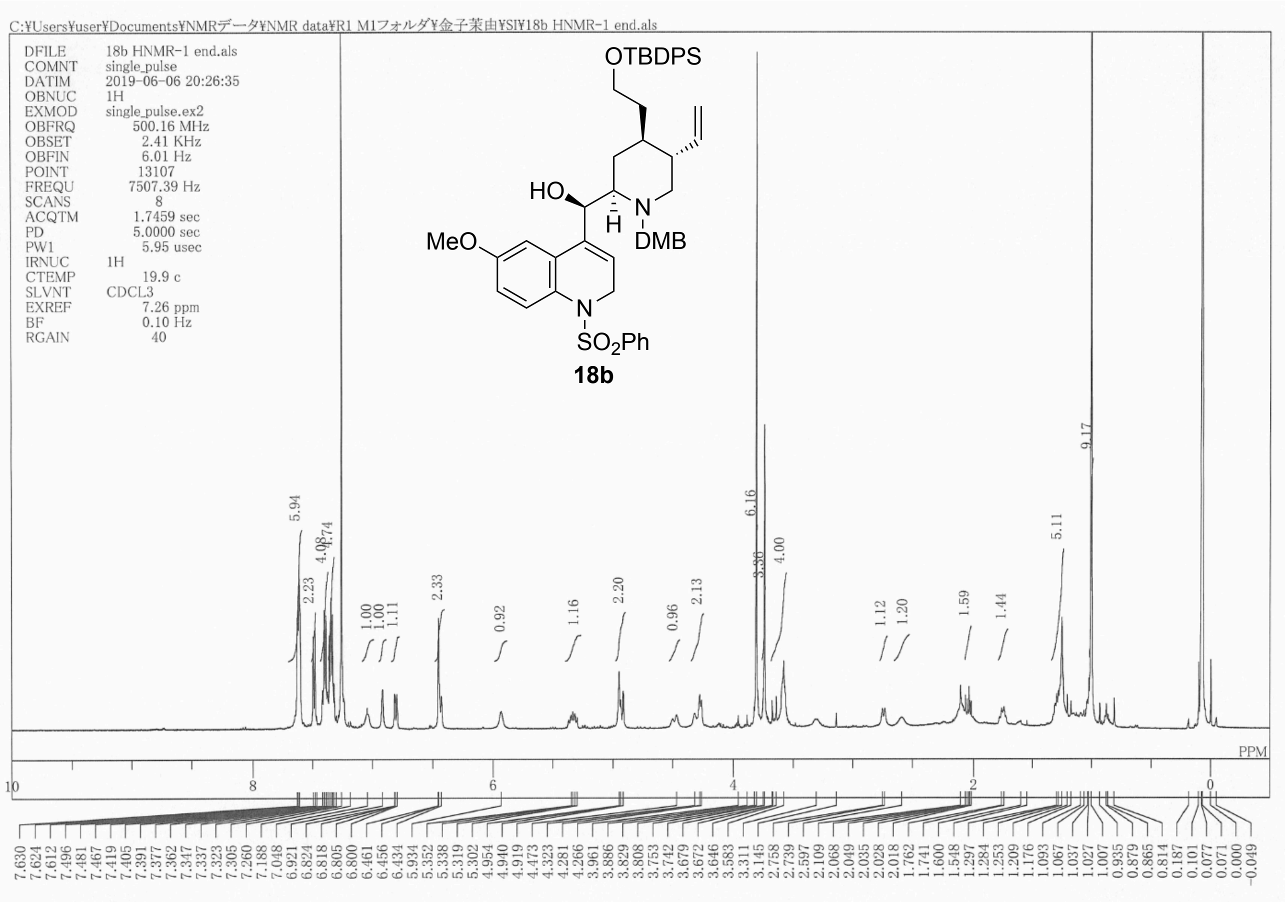
C:¥Users¥user¥Documents¥NMRデータ¥NMR data¥R1 M1フォルダ¥金子茉由¥SI¥18b HNMR-1 end.als
DFILE 18b HNMR-1 end.als
COMNT single_pulse
DATIM 2019-06-06 20:26:35
OBNUC 1H
EXMOD single_pulse.ex2
OBFRQ 500.16 MHz
OBSET 2.41 KHz
OBFIN 6.01 Hz
POINT 13107
FREQU 7507.39 Hz
SCANS 8
ACQTM 1.7459 sec
PD 5.0000 sec
PW1 5.95 usec
IRNUC 1H
CTEMP 19.9 c
SLVNT CDCL3
EXREF 7.26 ppm
BF 0.10 Hz
RGAIN 40
OTBDPS
HO
H
N
DMB
MeO
N
SO2Ph
18b
PPM
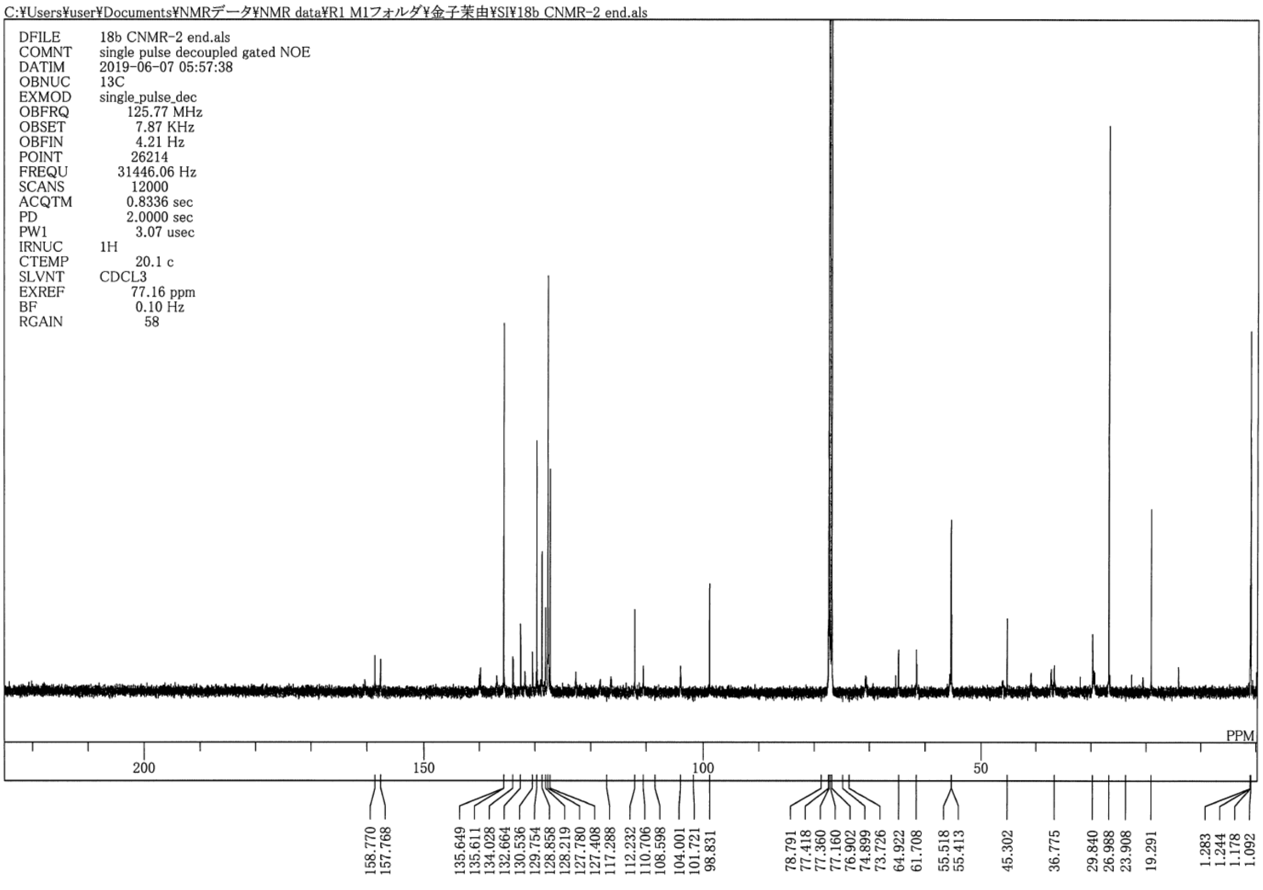
C:¥Users¥user¥Documents¥NMRデータ¥NMR data¥R1 M1フォルダ¥金子茉由¥SI¥18b CNMR-2 end.als
DFILE 18b CNMR-2 end.als
COMNT single pulse decoupled gated NOE
DATIM 2019-06-07 05:57:38
OBNUC 13C
EXMOD single_pulse_dec
OBFRQ 125.77 MHz
OBSET 7.87 KHz
OBFIN 4.21 Hz
POINT 26214
FREQU 31446.06 Hz
SCANS 12000
ACQTM 0.8336 sec
PD 2.0000 sec
PW1 3.07 usec
IRNUC 1H
CTEMP 20.1 c
SLVNT CDCL3
EXREF 77.16 ppm
BF 0.10 Hz
RGAIN 58
PPM
200
150
100
50
158.770
157.768
135.649
135.611
134.028
132.664
130.536
129.754
128.858
128.219
127.780
127.408
117.288
112.232
110.706
108.598
104.001
101.721
98.831
78.791
77.418
77.360
77.160
76.902
74.899
73.726
64.922
61.708
55.518
55.413
45.302
36.775
29.840
26.988
23.908
19.291
1.283
1.244
1.178
1.092

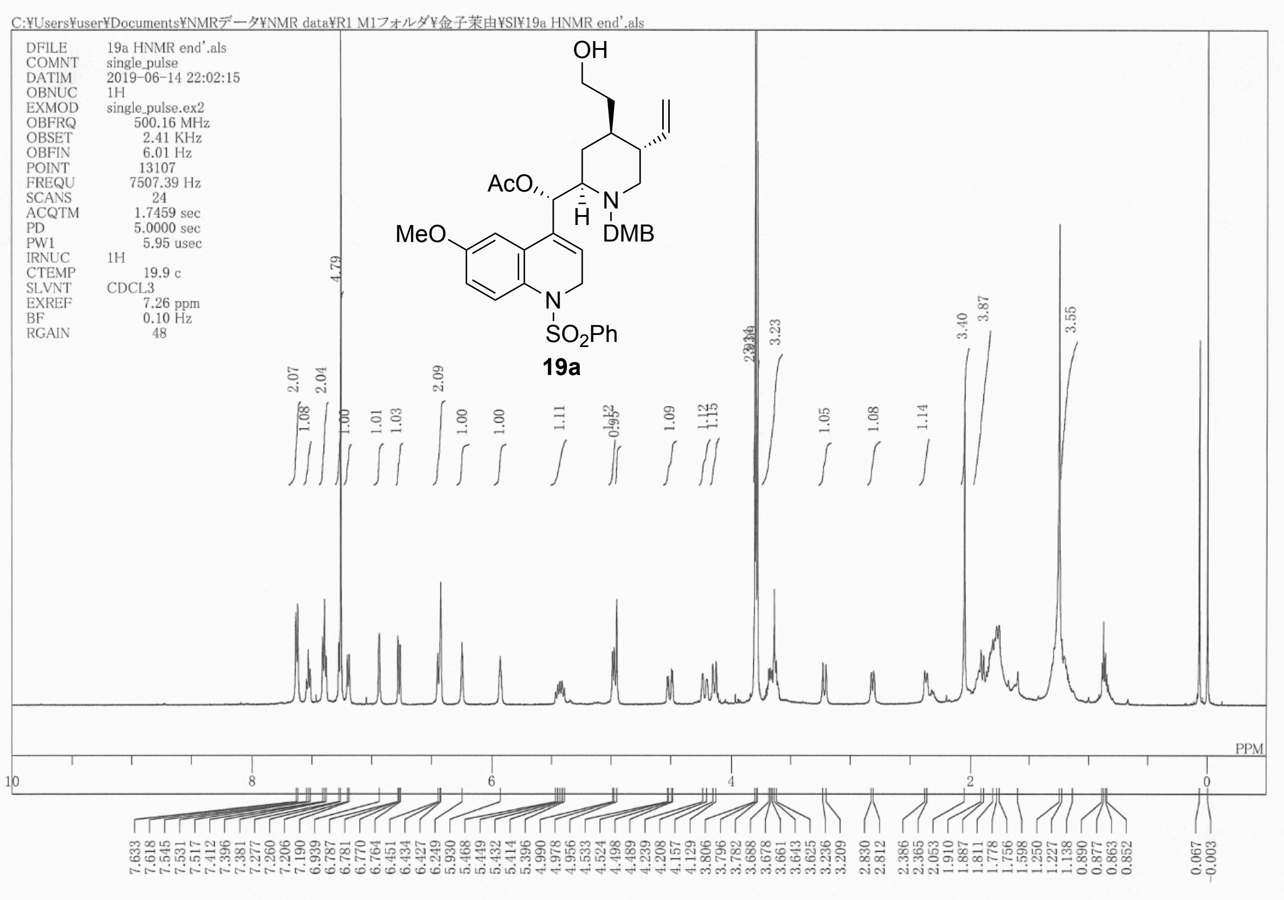
C:¥Users¥user¥Documents¥NMRデータ¥NMR data¥R1 M1フォルダ¥金子茉由¥SI¥19a HNMR end'.als
DFILE 19a HNMR end'.als
COMNT single_pulse
DATIM 2019-06-14 22:02:15
OBNUC 1H
EXMOD single_pulse.ex2
OBFRQ 500.16 MHz
OBSET 2.41 KHz
OBFIN 6.01 Hz
POINT 13107
FREQU 7507.39 Hz
SCANS 24
ACQTM 1.7459 sec
PD 5.0000 sec
PW1 5.95 usec
IRNUC 1H
CTEMP 19.9 c
SLVNT CDCL3
EXREF 7.26 ppm
BF 0.10 Hz
RGAIN 48
OH
AcO
H
N
DMB
MeO
N
SO2Ph
19a
PPM
7.633 7.618 7.545 7.531 7.517 7.412 7.396 7.381 7.277 7.260 7.206 7.190 6.939 6.787 6.781 6.770 6.764 6.451 6.434 6.427 6.249 5.930 5.468 5.449 5.432 5.414 5.396 4.990 4.978 4.956 4.533 4.524 4.498 4.489 4.239 4.208 4.157 4.129 3.806 3.796 3.782 3.688 3.678 3.661 3.643 3.625 3.236 3.209 2.830 2.812 2.386 2.365 2.053 1.910 1.887 1.811 1.778 1.756 1.598 1.250 1.227 1.138 0.890 0.877 0.863 0.852 0.067 -0.003

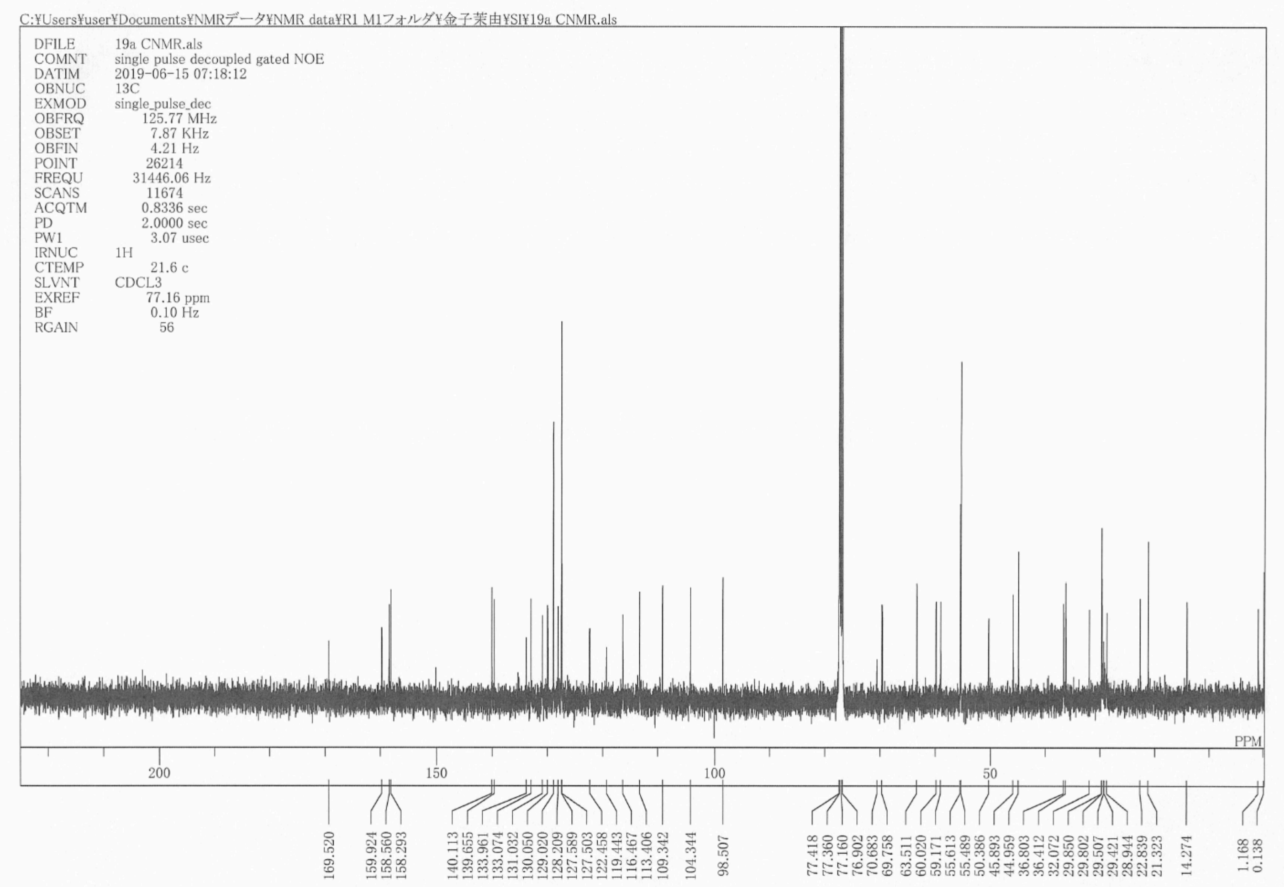
C:¥Users¥user¥Documents¥NMRデータ¥NMR data¥R1 M1フォルダ¥金子茉由¥SI¥19a CNMR.als
DFILE 19a CNMR.als
COMNT single pulse decoupled gated NOE
DATIM 2019-06-15 07:18:12
OBNUC 13C
EXMOD single_pulse_dec
OBFRQ 125.77 MHz
OBSET 7.87 KHz
OBFIN 4.21 Hz
POINT 26214
FREQU 31446.06 Hz
SCANS 11674
ACQTM 0.8336 sec
PD 2.0000 sec
PW1 3.07 usec
IRNUC 1H
CTEMP 21.6 c
SLVNT CDCL3
EXREF 77.16 ppm
BF 0.10 Hz
RGAIN 56
PPM
169.520 159.924 158.560 158.293 140.113 139.655 133.961 133.074 131.032 130.050 129.020 128.209 127.589 127.503 122.458 119.443 116.467 113.406 109.342 104.344 98.507 77.418 77.360 77.160 76.902 70.683 69.758 63.511 60.020 59.171 55.613 55.489 50.386 45.893 44.959 36.803 36.412 32.072 29.850 29.802 29.507 29.421 28.944 22.839 21.323 14.274 1.168 0.138

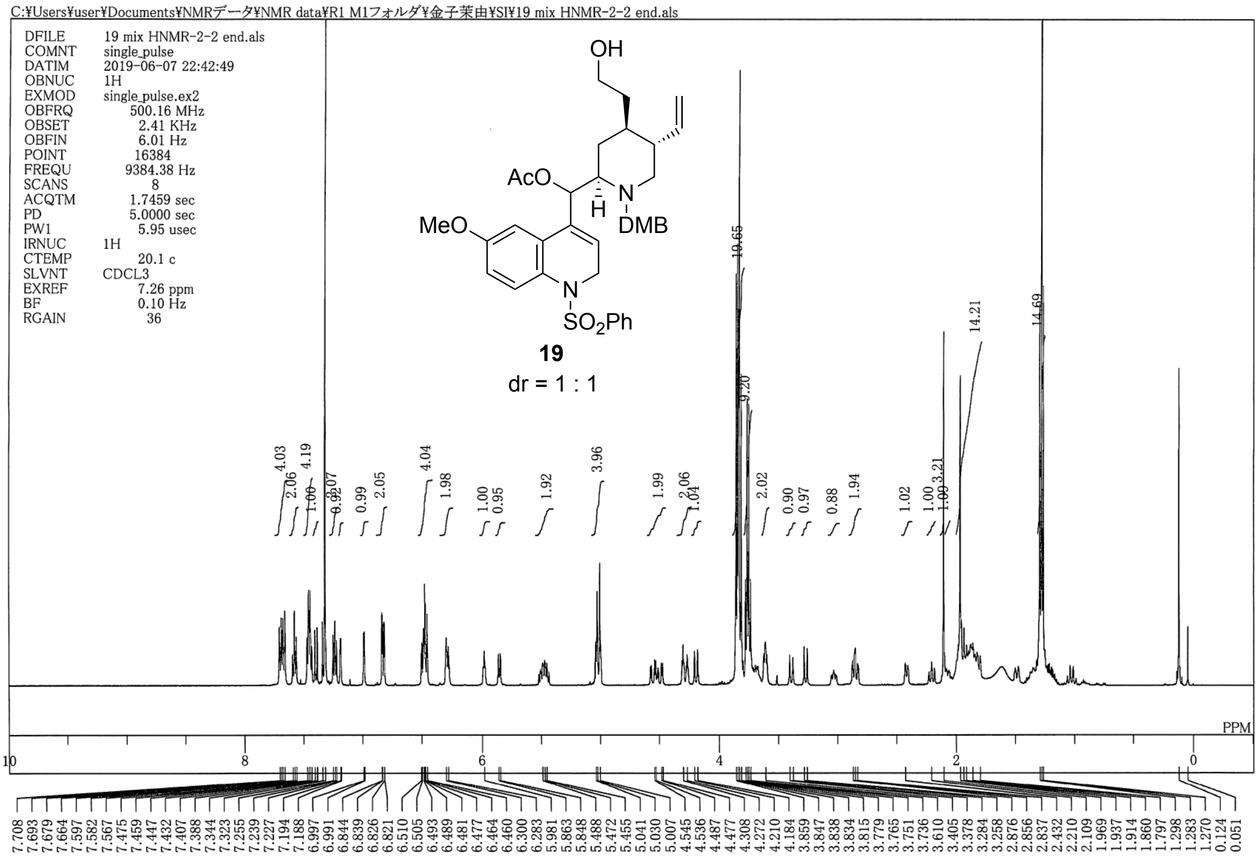
C:¥Users¥user¥Documents¥NMRデータ¥NMR data¥R1 M1フォルダ¥金子茉由¥SI¥19 mix HNMR-2-2 end.als
DFILE 19 mix HNMR-2-2 end.als
COMNT single_pulse
DATIM 2019-06-07 22:42:49
OBNUC 1H
EXMOD single_pulse.ex2
OBFRQ 500.16 MHz
OBSET 2.41 KHz
OBFIN 6.01 Hz
POINT 16384
FREQU 9384.38 Hz
SCANS 8
ACQTM 1.7459 sec
PD 5.0000 sec
PW1 5.95 usec
IRNUC 1H
CTEMP 20.1 c
SLVNT CDCL3
EXREF 7.26 ppm
BF 0.10 Hz
RGAIN 36
OH
AcO
H
N
DMB
MeO
N
SO2Ph
19
dr = 1 : 1
4.03 2.06 4.19 1.00 2.07 0.92 0.99 2.05 4.04 1.98 1.00 0.95 1.92 3.96 1.99 2.06 1.04 19.65 9.20 2.02 0.90 0.97 0.88 1.94 1.02 1.00 1.09 3.21 14.21 14.69
PPM
10 8 6 4 2 0
7.708 7.693 7.679 7.664 7.597 7.582 7.567 7.475 7.459 7.447 7.432 7.407 7.388 7.344 7.323 7.255 7.239 7.227 7.194 7.188 6.997 6.991 6.844 6.839 6.826 6.821 6.510 6.505 6.493 6.489 6.481 6.477 6.464 6.460 6.300 6.283 5.981 5.863 5.848 5.488 5.472 5.455 5.041 5.030 5.007 4.545 4.536 4.487 4.477 4.308 4.272 4.210 4.184 3.859 3.847 3.838 3.834 3.815 3.779 3.765 3.751 3.736 3.610 3.405 3.378 3.284 3.258 2.876 2.856 2.837 2.432 2.210 2.109 1.969 1.937 1.914 1.860 1.797 1.298 1.283 1.270 0.124 0.051

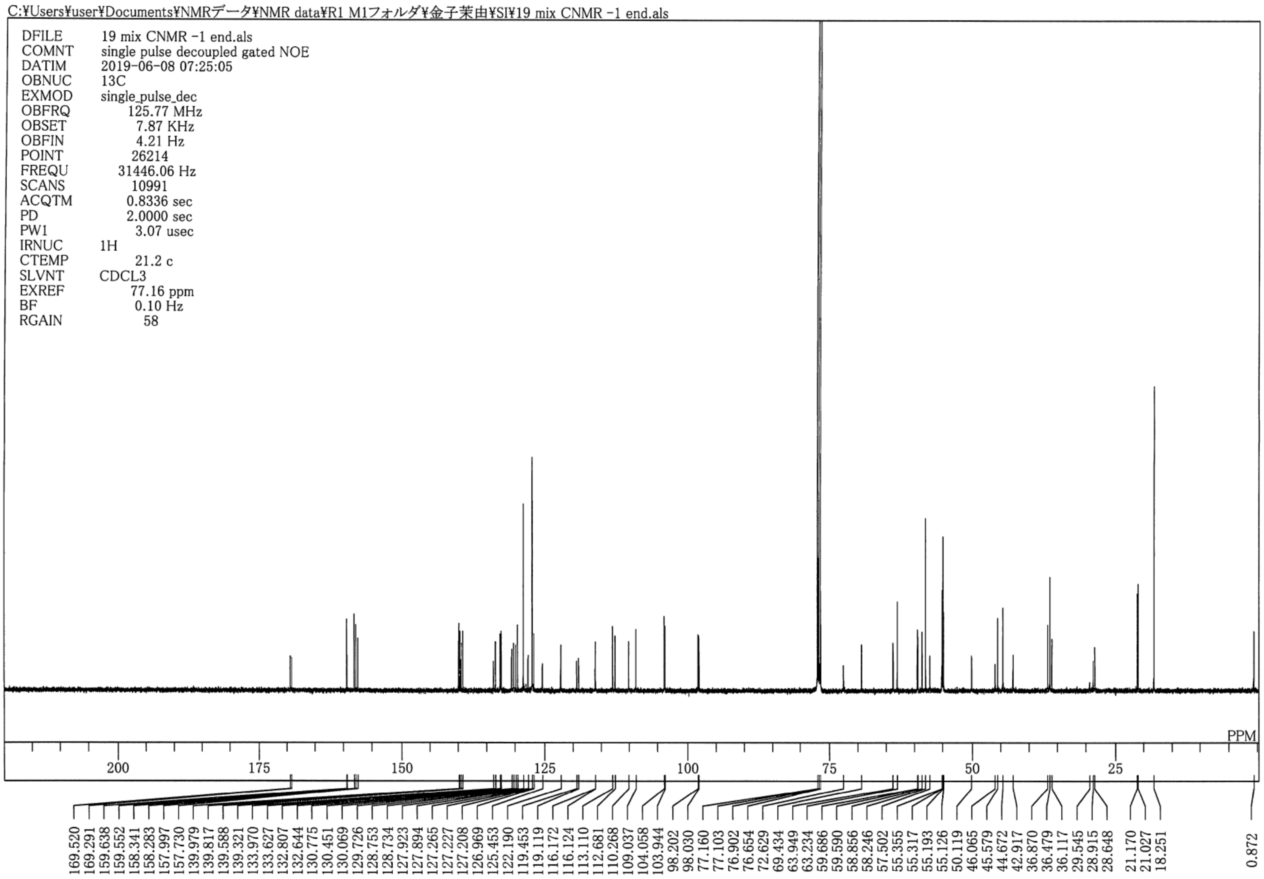
C:¥Users¥user¥Documents¥NMRデータ¥NMR data¥R1 M1フォルダ¥金子茉由¥SI¥19 mix CNMR -1 end.als
DFILE 19 mix CNMR -1 end.als
COMNT single pulse decoupled gated NOE
DATIM 2019-06-08 07:25:05
OBNUC 13C
EXMOD single_pulse_dec
OBFRQ 125.77 MHz
OBSET 7.87 KHz
OBFIN 4.21 Hz
POINT 26214
FREQU 31446.06 Hz
SCANS 10991
ACQTM 0.8336 sec
PD 2.0000 sec
PW1 3.07 usec
IRNUC 1H
CTEMP 21.2 c
SLVNT CDCL3
EXREF 77.16 ppm
BF 0.10 Hz
RGAIN 58
PPM
200 175 150 125 100 75 50 25
169.520 169.291 159.638 159.552 158.341 158.283 157.997 157.730 139.979 139.817 139.588 139.321 133.970 133.627 132.807 132.644 130.775 130.451 130.069 129.726 128.753 128.734 127.923 127.894 127.265 127.227 127.208 126.969 125.453 122.190 119.453 119.119 116.172 116.124 113.110 112.681 110.268 109.037 104.058 103.944 98.202 98.030 77.160 77.103 76.902 76.654 72.629 69.434 63.949 63.234 59.686 59.590 58.856 58.246 57.502 55.355 55.317 55.193 55.126 50.119 46.065 45.579 44.672 42.917 36.870 36.479 36.117 29.545 28.915 28.648 21.170 21.027 18.251 0.872

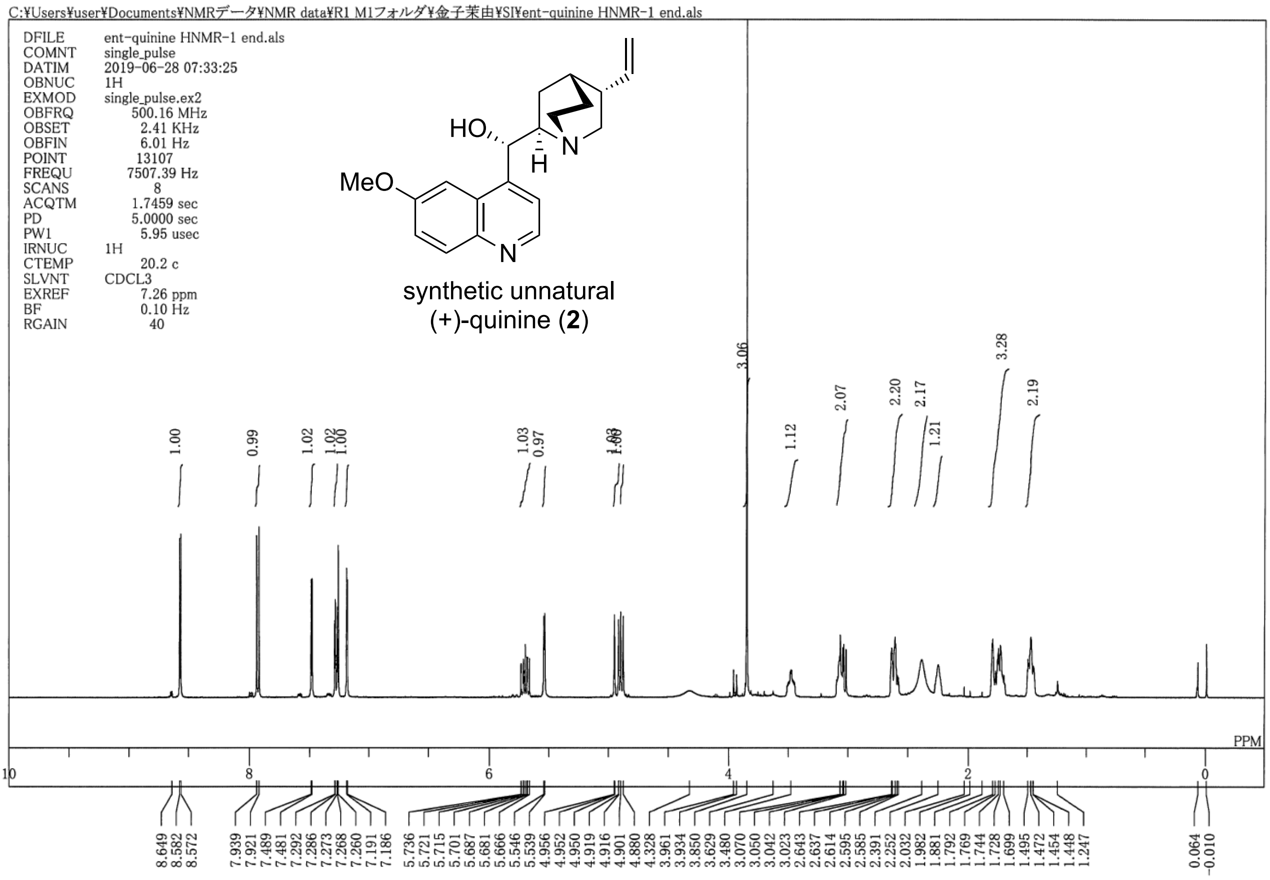
C:¥Users¥user¥Documents¥NMRデータ¥NMR data¥R1 M1フォルダ¥金子茉由¥SI¥ent-quinine HNMR-1 end.als
DFILE ent-quinine HNMR-1 end.als
COMNT single_pulse
DATIM 2019-06-28 07:33:25
OBNUC 1H
EXMOD single_pulse.ex2
OBFRQ 500.16 MHz
OBSET 2.41 KHz
OBFIN 6.01 Hz
POINT 13107
FREQU 7507.39 Hz
SCANS 8
ACQTM 1.7459 sec
PD 5.0000 sec
PW1 5.95 usec
IRNUC 1H
CTEMP 20.2 c
SLVNT CDCL3
EXREF 7.26 ppm
BF 0.10 Hz
RGAIN 40
HO
H
N
MeO
N
synthetic unnatural
(+)-quinine (2)
1.00 0.99 1.02 1.02 1.00 1.03 0.97 3.06 1.12 2.07 2.20 2.17 1.21 3.28 2.19
PPM
10 8 6 4 2 0
8.649 8.582 8.572 7.939 7.921 7.489 7.481 7.292 7.286 7.273 7.268 7.260 7.191 7.186 5.736 5.721 5.715 5.701 5.687 5.681 5.666 5.546 5.539 4.956 4.952 4.950 4.919 4.916 4.901 4.880 4.328 3.961 3.934 3.850 3.629 3.480 3.070 3.050 3.042 3.023 2.643 2.637 2.614 2.595 2.585 2.391 2.252 2.032 1.982 1.881 1.792 1.769 1.744 1.728 1.699 1.495 1.472 1.454 1.448 1.247 0.064 -0.010

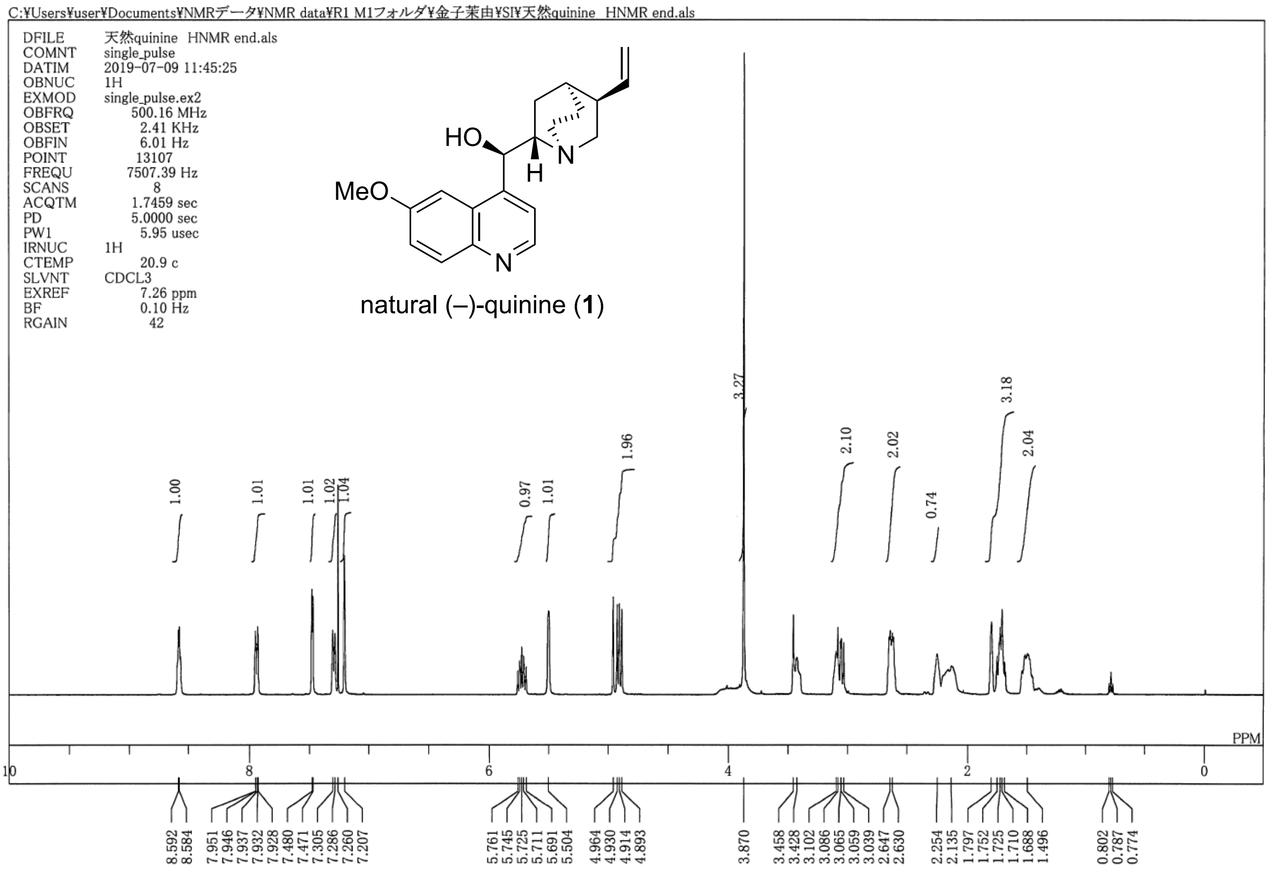
C:¥Users¥user¥Documents¥NMRデータ¥NMR data¥R1 M1フォルダ¥金子茉由¥SI¥天然quinine HNMR end.als
DFILE 天然quinine HNMR end.als
COMNT single_pulse
DATIM 2019-07-09 11:45:25
OBNUC 1H
EXMOD single_pulse.ex2
OBFRQ 500.16 MHz
OBSET 2.41 KHz
OBFIN 6.01 Hz
POINT 13107
FREQU 7507.39 Hz
SCANS 8
ACQTM 1.7459 sec
PD 5.0000 sec
PW1 5.95 usec
IRNUC 1H
CTEMP 20.9 c
SLVNT CDCL3
EXREF 7.26 ppm
BF 0.10 Hz
RGAIN 42
HO
H
N
MeO
N
natural (–)-quinine (1)
1.00 1.01 1.01 1.02 1.04 0.97 1.01 1.96 3.27 2.10 2.02 0.74 3.18 2.04
PPM
10 8 6 4 2 0
8.592 8.584 7.951 7.946 7.937 7.932 7.928 7.480 7.471 7.305 7.286 7.260 7.207 5.761 5.745 5.725 5.711 5.691 5.504 4.964 4.930 4.914 4.893 3.870 3.458 3.428 3.102 3.086 3.065 3.059 3.039 2.647 2.630 2.254 2.135 1.797 1.752 1.725 1.710 1.688 1.496 0.802 0.787 0.774

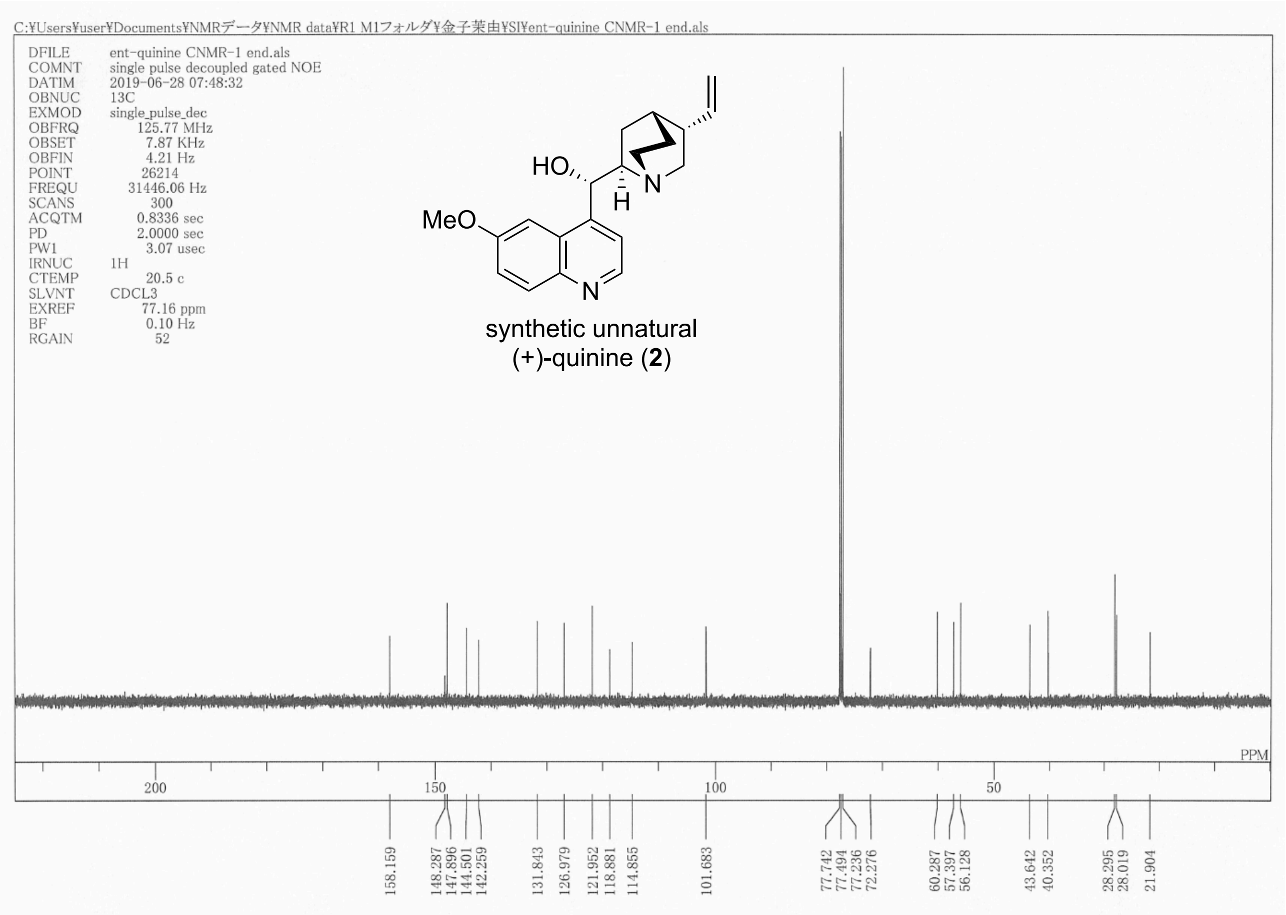
C:¥Users¥user¥Documents¥NMRデータ¥NMR data¥R1 M1フォルダ¥金子茉由¥SI¥ent-quinine CNMR-1 end.als
DFILE ent-quinine CNMR-1 end.als
COMNT single pulse decoupled gated NOE
DATIM 2019-06-28 07:48:32
OBNUC 13C
EXMOD single_pulse_dec
OBFRQ 125.77 MHz
OBSET 7.87 KHz
OBFIN 4.21 Hz
POINT 26214
FREQU 31446.06 Hz
SCANS 300
ACQTM 0.8336 sec
PD 2.0000 sec
PW1 3.07 usec
IRNUC 1H
CTEMP 20.5 c
SLVNT CDCL3
EXREF 77.16 ppm
BF 0.10 Hz
RGAIN 52
HO
H
N
MeO
N
synthetic unnatural
(+)-quinine (2)
PPM
200
150
100
50
158.159
148.287
147.896
144.501
142.259
131.843
126.979
121.952
118.881
114.855
101.683
77.742
77.494
77.236
72.276
60.287
57.397
56.128
43.642
40.352
28.295
28.019
21.904

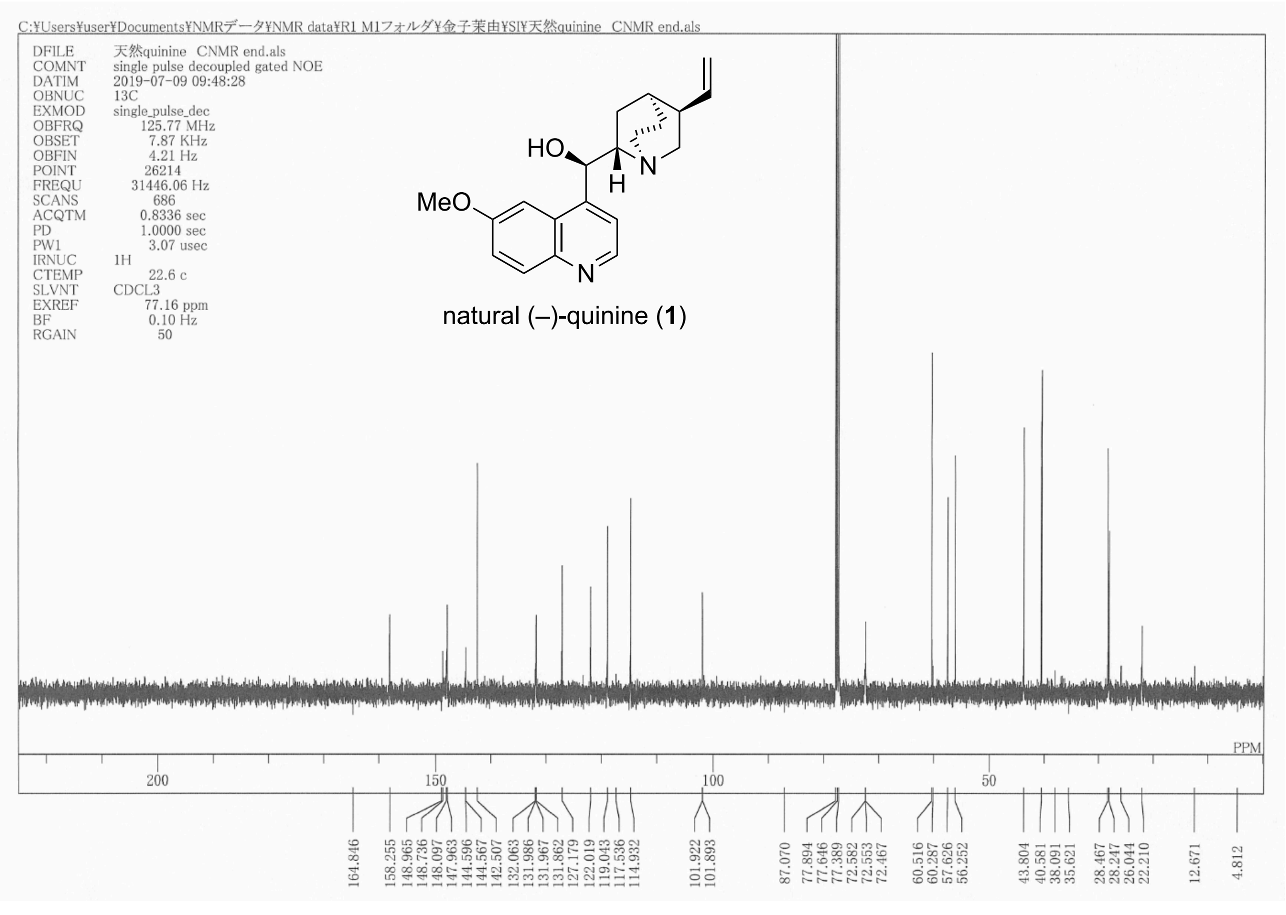
C:¥Users¥user¥Documents¥NMRデータ¥NMR data¥R1 M1フォルダ¥金子茉由¥SI¥天然quinine CNMR end.als
DFILE 天然quinine CNMR end.als
COMNT single pulse decoupled gated NOE
DATIM 2019-07-09 09:48:28
OBNUC 13C
EXMOD single_pulse_dec
OBFRQ 125.77 MHz
OBSET 7.87 KHz
OBFIN 4.21 Hz
POINT 26214
FREQU 31446.06 Hz
SCANS 686
ACQTM 0.8336 sec
PD 1.0000 sec
PW1 3.07 usec
IRNUC 1H
CTEMP 22.6 c
SLVNT CDCL3
EXREF 77.16 ppm
BF 0.10 Hz
RGAIN 50
HO
H
N
MeO
N
natural (–)-quinine (1)
PPM
200
150
100
50
164.846
158.255
148.965
148.736
148.097
147.963
144.596
144.567
142.507
132.063
131.986
131.967
131.862
127.179
122.019
119.043
117.536
114.932
101.922
101.893
87.070
77.894
77.646
77.389
72.582
72.553
72.467
60.516
60.287
57.626
56.252
43.804
40.581
38.091
35.621
28.467
28.247
26.044
22.210
12.671
4.812

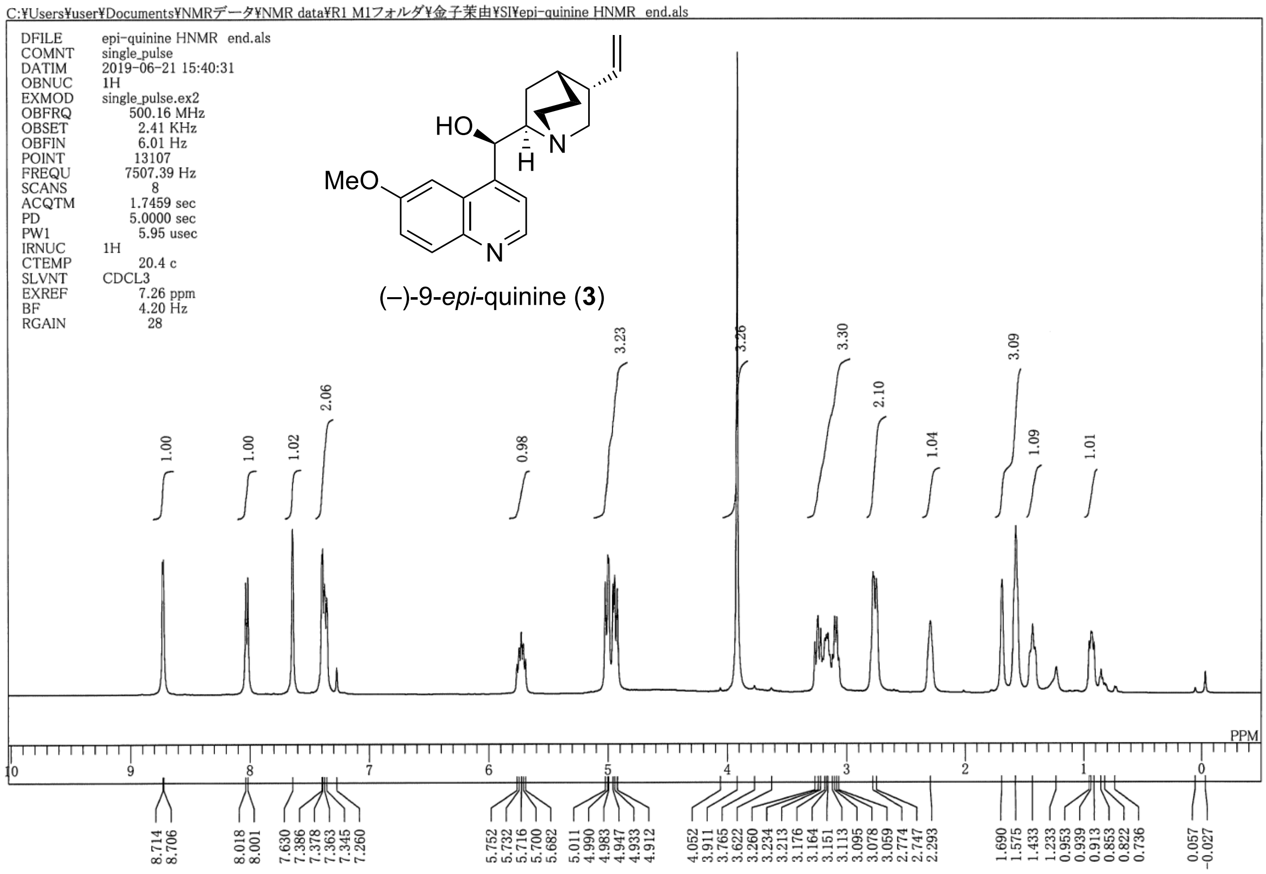

C:¥Users¥user¥Documents¥NMRデータ¥NMR data¥R1 M1フォルダ¥金子茉由¥SI¥epi-quinine HNMR end.als
DFILE epi-quinine HNMR end.als
COMNT single_pulse
DATIM 2019-06-21 15:40:31
OBNUC 1H
EXMOD single_pulse.ex2
OBFRQ 500.16 MHz
OBSET 2.41 KHz
OBFIN 6.01 Hz
POINT 13107
FREQU 7507.39 Hz
SCANS 8
ACQTM 1.7459 sec
PD 5.0000 sec
PW1 5.95 usec
IRNUC 1H
CTEMP 20.4 c
SLVNT CDCL3
EXREF 7.26 ppm
BF 4.20 Hz
RGAIN 28
HO
MeO
H
N
N
(–)-9-epi-quinine (3)
1.00 1.00 1.02 2.06 0.98 3.23 3.26 3.30 2.10 1.04 3.09 1.09 1.01
PPM
10 9 8 7 6 5 4 3 2 1 0
8.714 8.706 8.018 8.001 7.630 7.386 7.378 7.363 7.345 7.260 5.752 5.732 5.716 5.700 5.682 5.011 4.990 4.983 4.947 4.933 4.912 4.052 3.911 3.765 3.622 3.260 3.234 3.213 3.176 3.164 3.151 3.113 3.095 3.078 3.059 2.774 2.747 2.293 1.690 1.575 1.433 1.233 0.953 0.939 0.913 0.853 0.822 0.736 0.057 -0.027

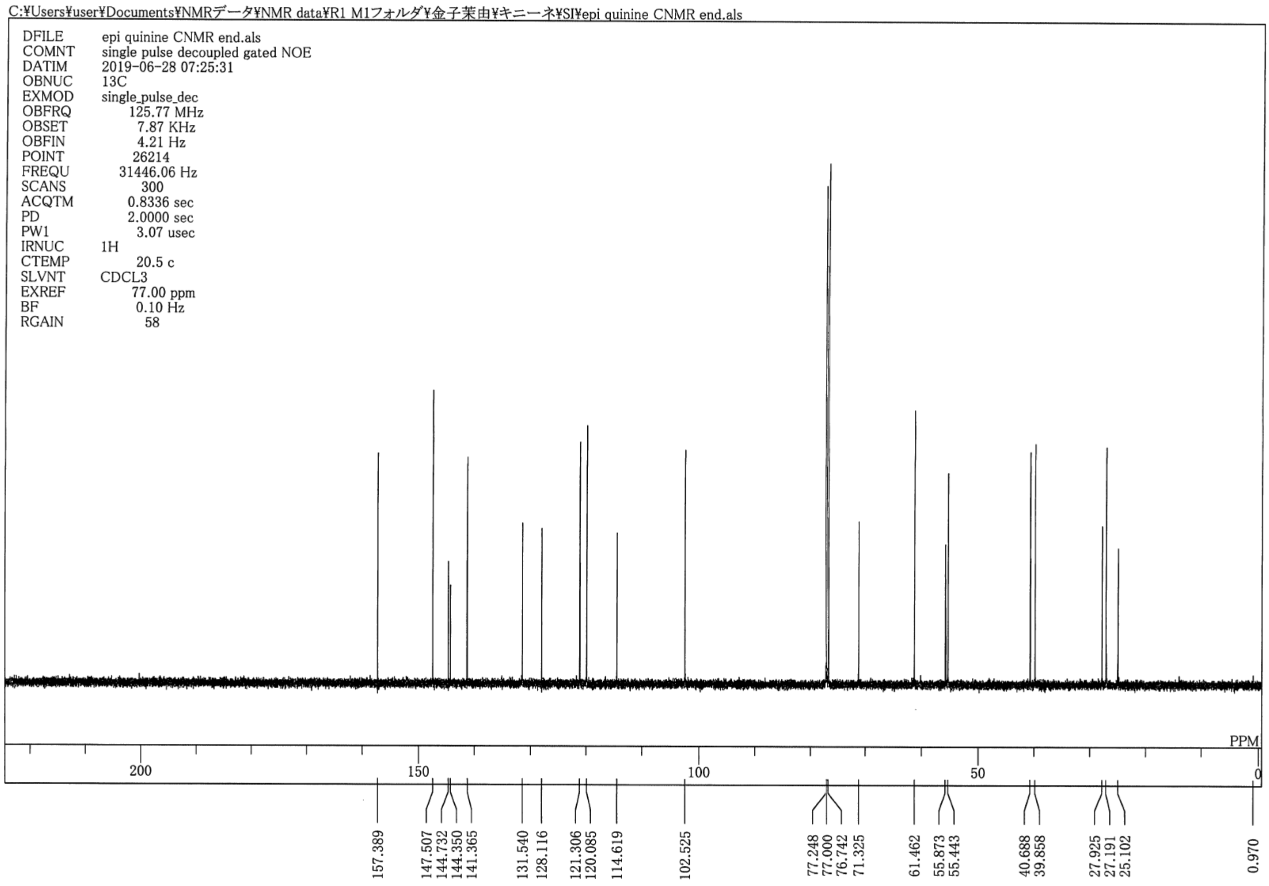

C:¥Users¥user¥Documents¥NMRデータ¥NMR data¥R1 M1フォルダ¥金子茉由¥キニーネ¥SI¥epi quinine CNMR end.als
DFILE epi quinine CNMR end.als
COMNT single pulse decoupled gated NOE
DATIM 2019-06-28 07:25:31
OBNUC 13C
EXMOD single_pulse_dec
OBFRQ 125.77 MHz
OBSET 7.87 KHz
OBFIN 4.21 Hz
POINT 26214
FREQU 31446.06 Hz
SCANS 300
ACQTM 0.8336 sec
PD 2.0000 sec
PW1 3.07 usec
IRNUC 1H
CTEMP 20.5 c
SLVNT CDCL3
EXREF 77.00 ppm
BF 0.10 Hz
RGAIN 58
PPM
200 150 100 50 0
157.389 147.507 144.732 144.350 141.365 131.540 128.116 121.306 120.085 114.619 102.525 77.248 77.000 76.742 71.325 61.462 55.873 55.443 40.688 39.858 27.925 27.191 25.102 0.970

=== Shimadzu LabSolutions Report ===

Sample Name	: kini unnat 10 ih		
Sample ID	: kaneko		
Data Filename	: kini unnat 10 ih.lcd		
Method Filename	: 10%iPrOH-Hex-floe0.25.lcm		
Batch Filename	:		
Vial#	: 1-1	Sample Type	: -¢'m
Injection Volume	: 20 uL		
Date Acquired	: 2019/07/16 19:41:38 Acquired by	: System Administrator	
Date Processed	: 2019/07/16 21:02:24 Processed by	: System Administrator	

<Chromatogram>

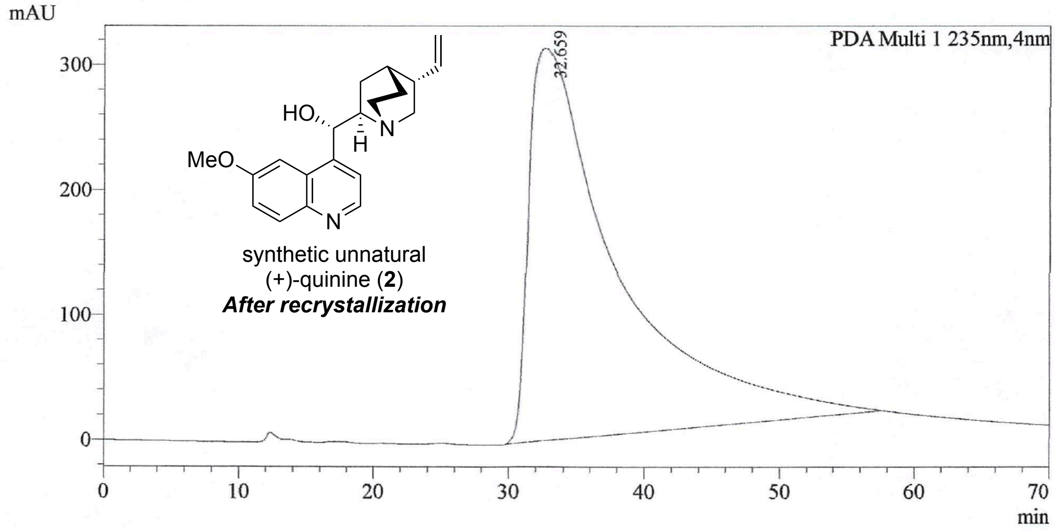

<Peak Table>

PDA Ch1 235nm

Peak#	Ret. Time	Area	Height	Conc.	Name
1	32.659	144074762	313968	100.000	
‡Œv		144074762	313968		

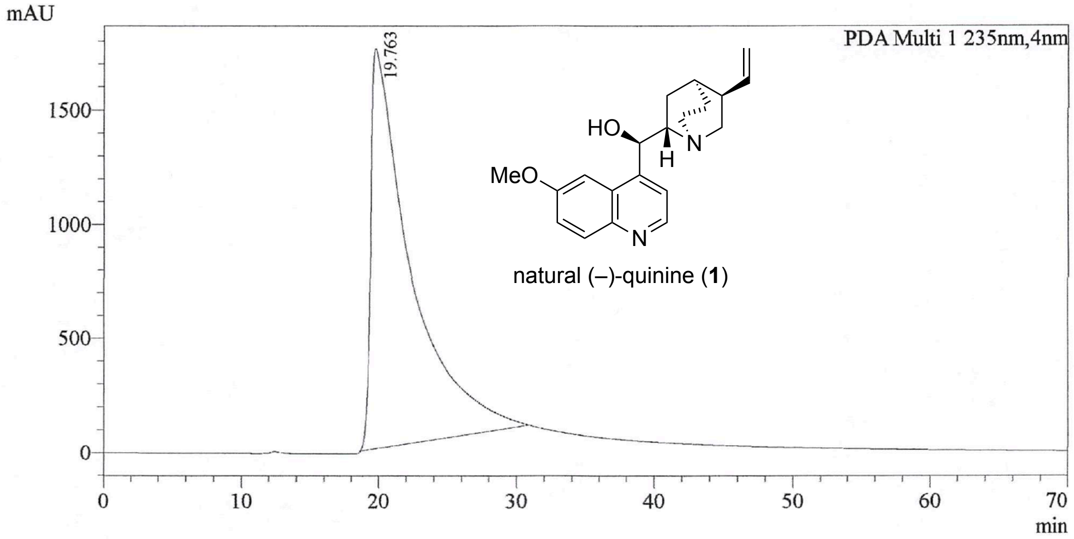

<Peak Table>

PDA Ch1 235nm

Peak#	Ret. Time	Area	Height	Conc.	Name
1	19.763	351504692	1750259	100.000	
‡Œv		351504692	1750259		
